# Author Correction: N4-acetylcytidine (ac4C) promotes mRNA localization to stress granules

**DOI:** 10.1038/s44319-026-00884-4

**Published:** 2026-07-29

**Authors:** Pavel Kudrin, Ankita Singh, David Meierhofer, Anna Kuśnierczyk, Ulf Andersson Vang Ørom

**Affiliations:** 1 Institute of Molecular Biology and Genetics, Aarhus University, 8000 Aarhus, Denmark; 2 Institute of Biomedicine and Translational Medicine, University of Tartu, 50411 Tartu, Estonia; 3 Institute of Biomedicine, Aarhus University, 8000 Aarhus, Denmark; 4 Max Planck Institute for Molecular Genetics, 14195 Berlin, Germany; 5 Proteomics and Modomics Experimental Core (PROMEC), Department of Clinical and Molecular Medicine, Norwegian University of Science and Technology and the Central Norway Regional Health Authority, 7030 Trondheim, Norway

## Abstract

**Figure:**
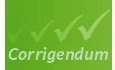

**Correction to:** *EMBO Reports* (2024) 25:1814–1834. https://doi.org/10.1038/s44319-024-00098-6 | Published online 27 February 2024

The authors contacted the journal after identifying an error in the Methods section of the published article.

**The Materials and Methods section is corrected**.

In the ‘Tissue culture and purification of SG’ section, the amount of anti-G3BP antibody (#61559, CellSignal) used for immunoprecipitation is incorrectly stated.

The error is corrected from:

*…incubated at 4°C for at least 3 h, rotating with 5 mg anti-G3BP antibody (#61559, CellSignal) prebound to 60 μl Protein G Dynabeads (Invitrogen)*.

To: (Changes in bold.)

*…incubated at 4°C for at least 3 h, rotating with anti-G3BP antibody (#61559, CellSignal) at a* ***1:50 dilution (20 µl in a 1 ml reaction)**** prebound to 60 μl Protein G Dynabeads (Invitrogen)*.

**Author Statement:**

We have identified a typo in the methods section that we hereby wish to correct. The error does not affect the results or conclusions of the paper.

