# Supplementary figures and images for: N4-acetylcytidine (ac4C) promotes mRNA localization to stress granules
Source: EMBO Rep. 2024 Feb 27;25(4):1814–34. doi: 10.1038/s44319-024-00098-6 (PMC11014937; doi:10.1038/s44319-024-00098-6)

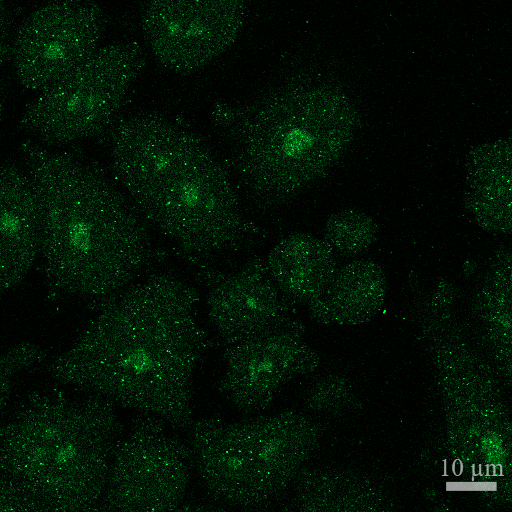

Supplement: Supplementary file 4 — Source Data Fig. 1 [file 44319_2024_98_MOESM4_ESM.zip › Figure 1/1A/wt_ctrl_100x oil_AF488-ac4C.tif]

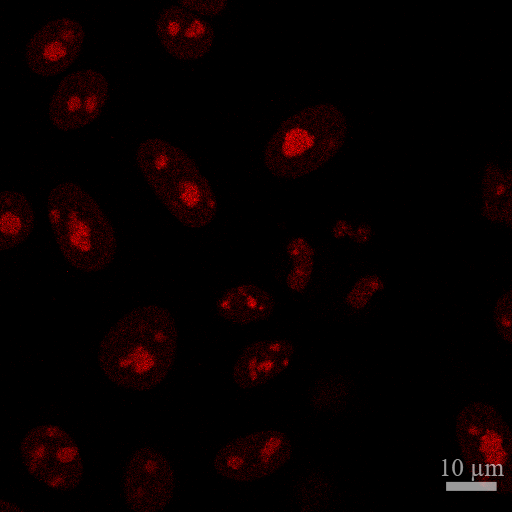

Supplement: Supplementary file 4 — Source Data Fig. 1 [file 44319_2024_98_MOESM4_ESM.zip › Figure 1/1A/wt_ctrl_100x oil_AF594-NCL.tif]

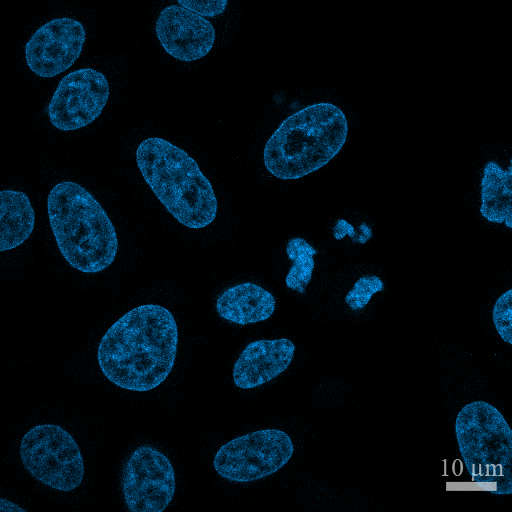

Supplement: Supplementary file 4 — Source Data Fig. 1 [file 44319_2024_98_MOESM4_ESM.zip › Figure 1/1A/wt_ctrl_100x oil_DAPI.tif]

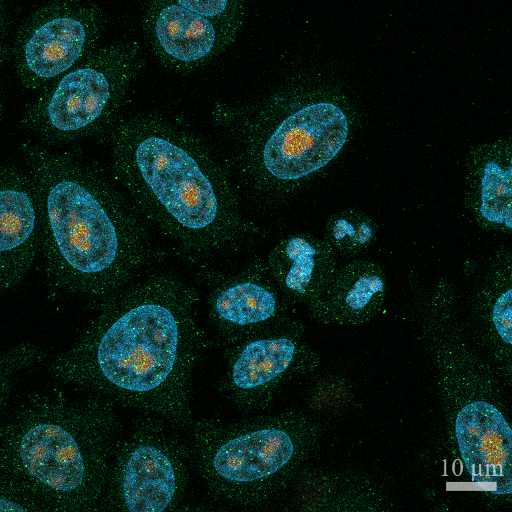

Supplement: Supplementary file 4 — Source Data Fig. 1 [file 44319_2024_98_MOESM4_ESM.zip › Figure 1/1A/wt_ctrl_100x oil_merged.tif]

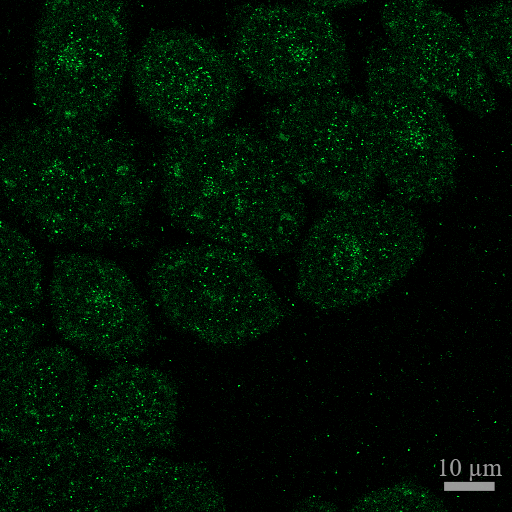

Supplement: Supplementary file 4 — Source Data Fig. 1 [file 44319_2024_98_MOESM4_ESM.zip › Figure 1/1B/wt_arsenite_60 min_100x oil_AF488-ac4C.tif]

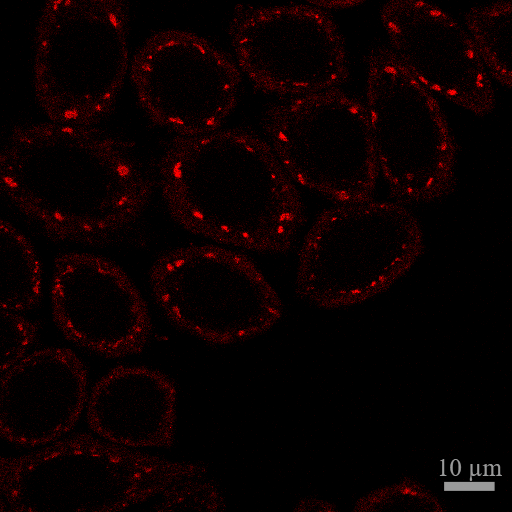

Supplement: Supplementary file 4 — Source Data Fig. 1 [file 44319_2024_98_MOESM4_ESM.zip › Figure 1/1B/wt_arsenite_60 min_100x oil_AF594-G3BP.tif]

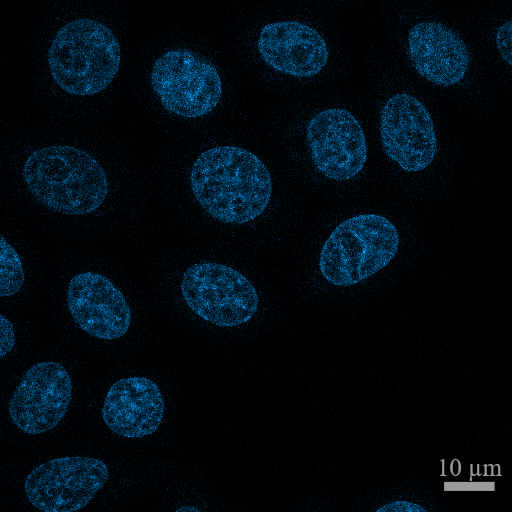

Supplement: Supplementary file 4 — Source Data Fig. 1 [file 44319_2024_98_MOESM4_ESM.zip › Figure 1/1B/wt_arsenite_60 min_100x oil_DAPI.tif]

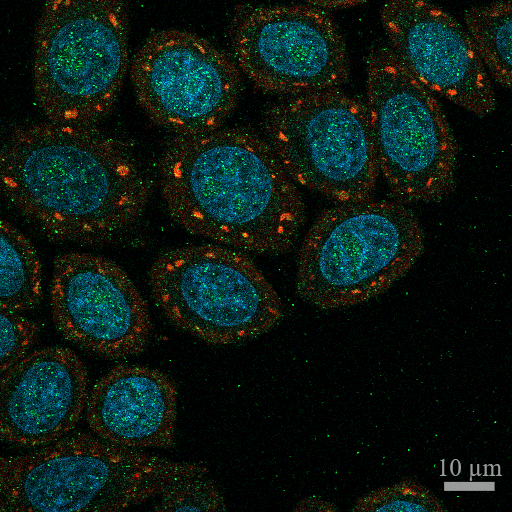

Supplement: Supplementary file 4 — Source Data Fig. 1 [file 44319_2024_98_MOESM4_ESM.zip › Figure 1/1B/wt_arsenite_60 min_100x oil_merged.tif]

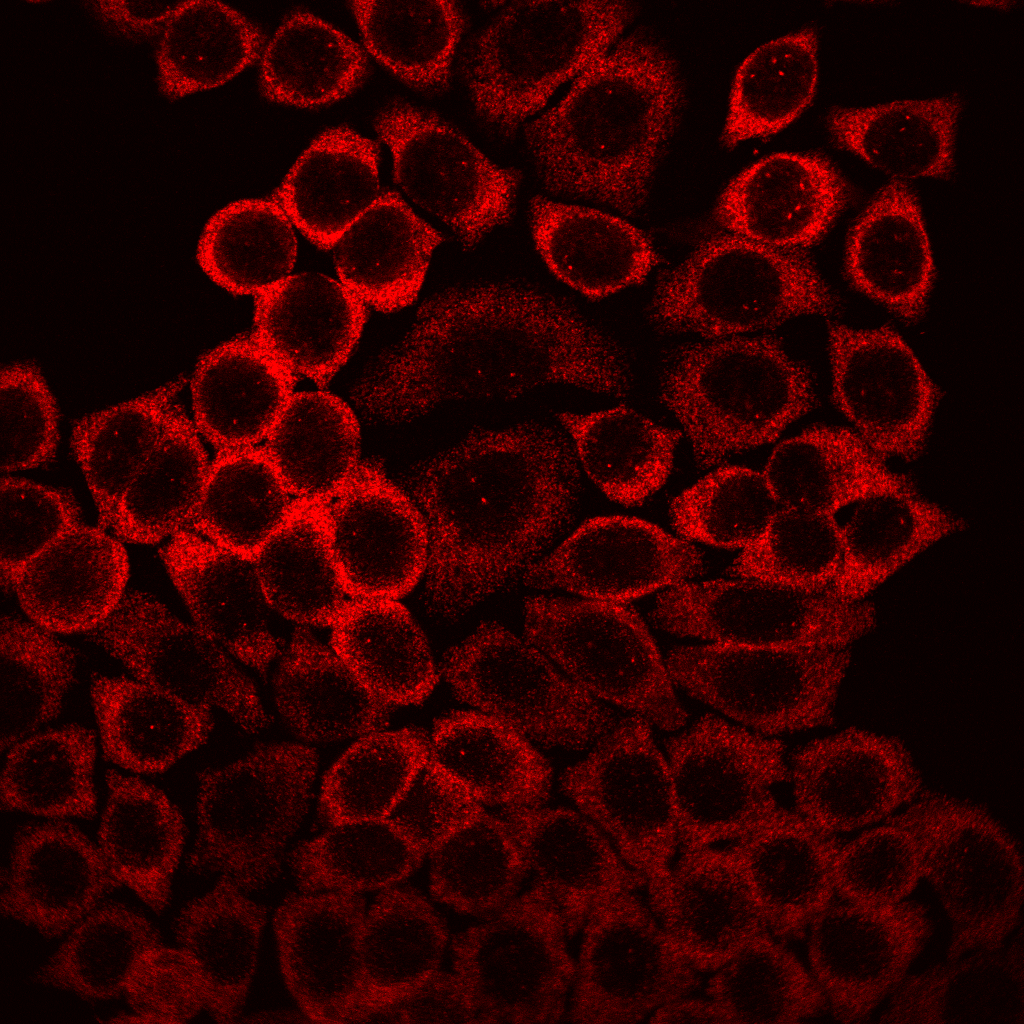

Supplement: Supplementary file 5 — Source Data Fig. 5 [file 44319_2024_98_MOESM5_ESM.zip › 5A/WT_0/wt_ctrl_60x oil 1_gapdh.tif]

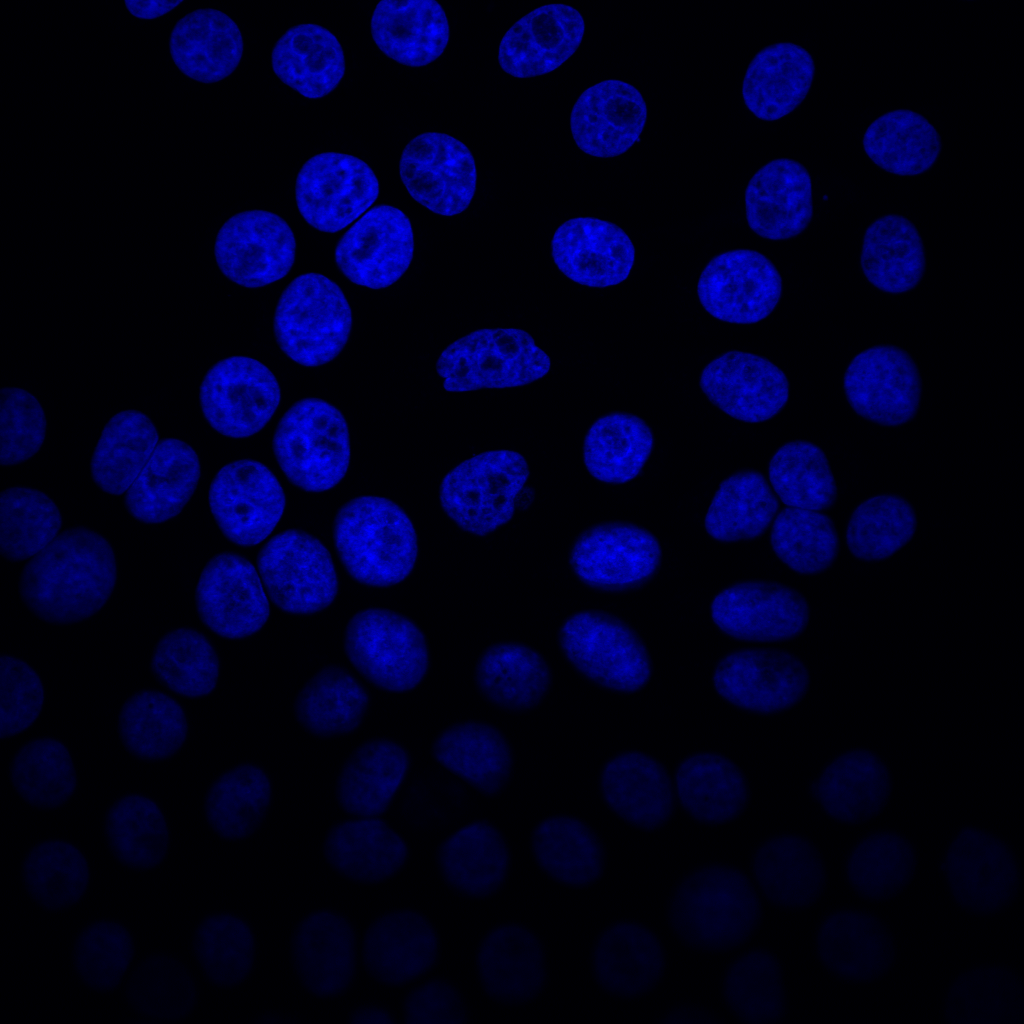

Supplement: Supplementary file 5 — Source Data Fig. 5 [file 44319_2024_98_MOESM5_ESM.zip › 5A/WT_0/wt_ctrl_60x oil 1_dapi.tif]

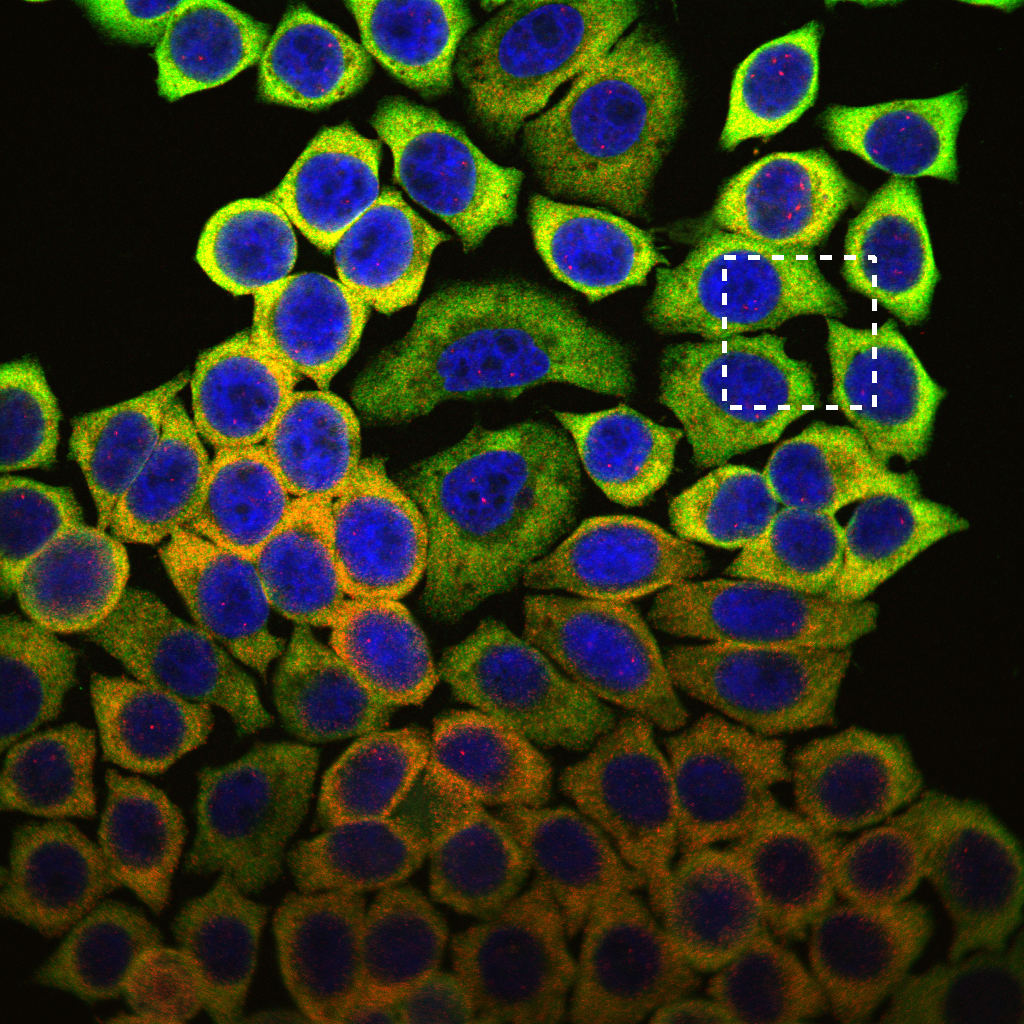

Supplement: Supplementary file 5 — Source Data Fig. 5 [file 44319_2024_98_MOESM5_ESM.zip › 5A/WT_0/wt_ctrl_60x oil 1_merged_dashed cropped area.tif]

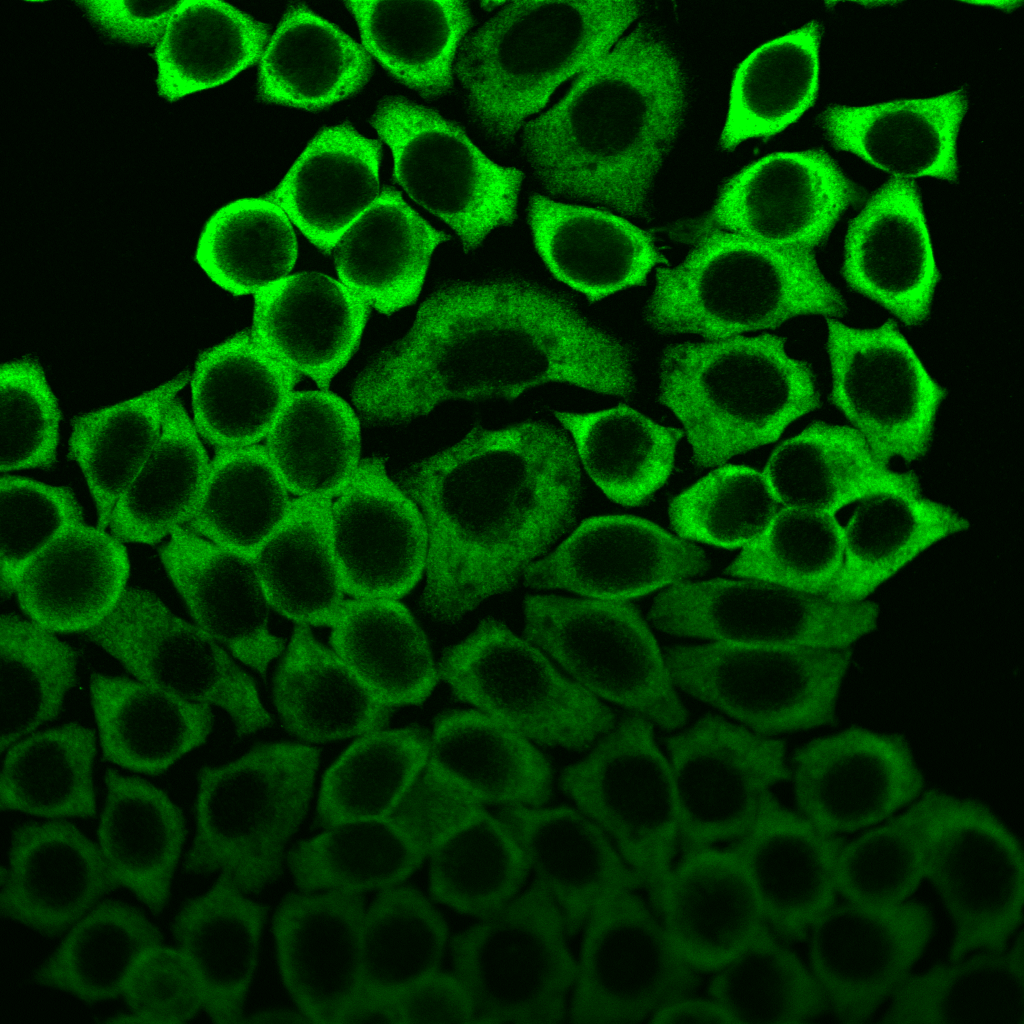

Supplement: Supplementary file 5 — Source Data Fig. 5 [file 44319_2024_98_MOESM5_ESM.zip › 5A/WT_0/wt_ctrl_60x oil 1_g3bp.tif]

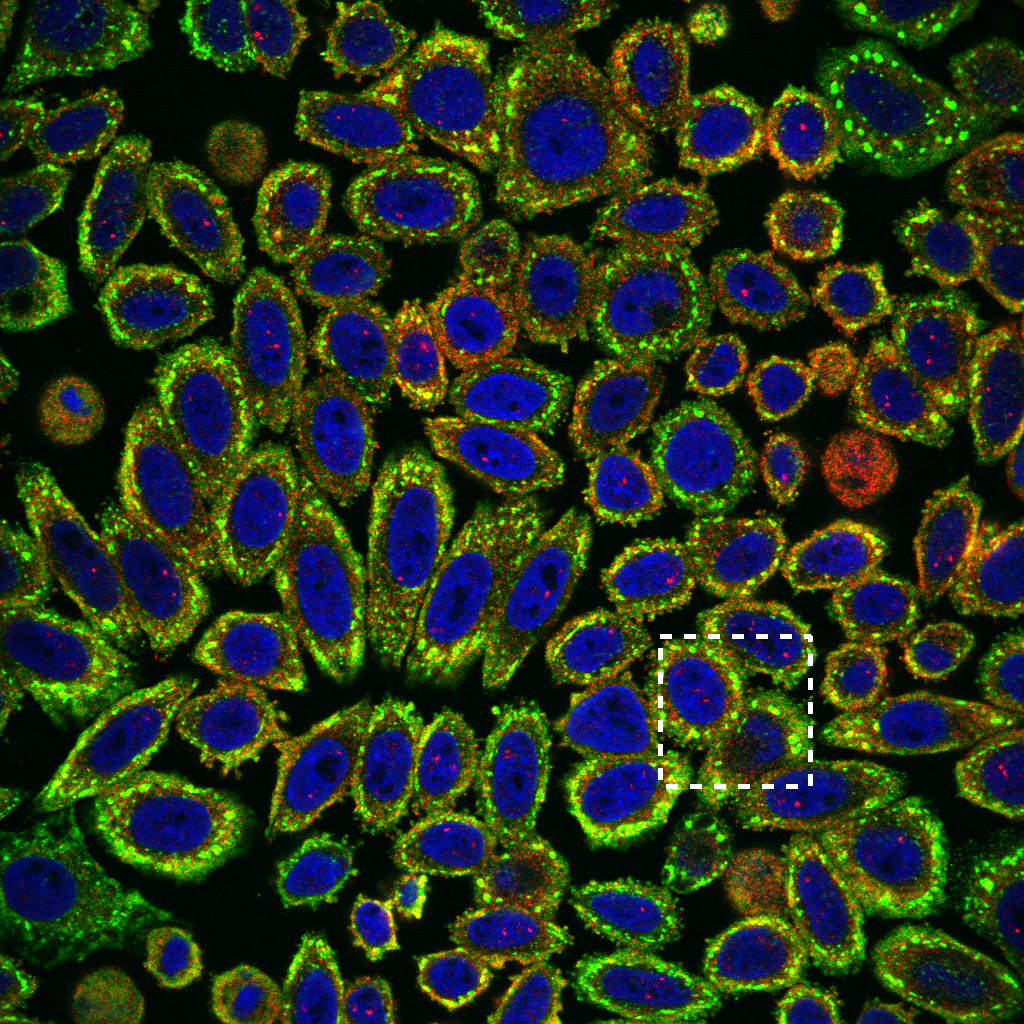

Supplement: Supplementary file 5 — Source Data Fig. 5 [file 44319_2024_98_MOESM5_ESM.zip › 5A/WT_30/wt_30min_ctrl_60x oil 2_merged_dashed cropped area.tif]

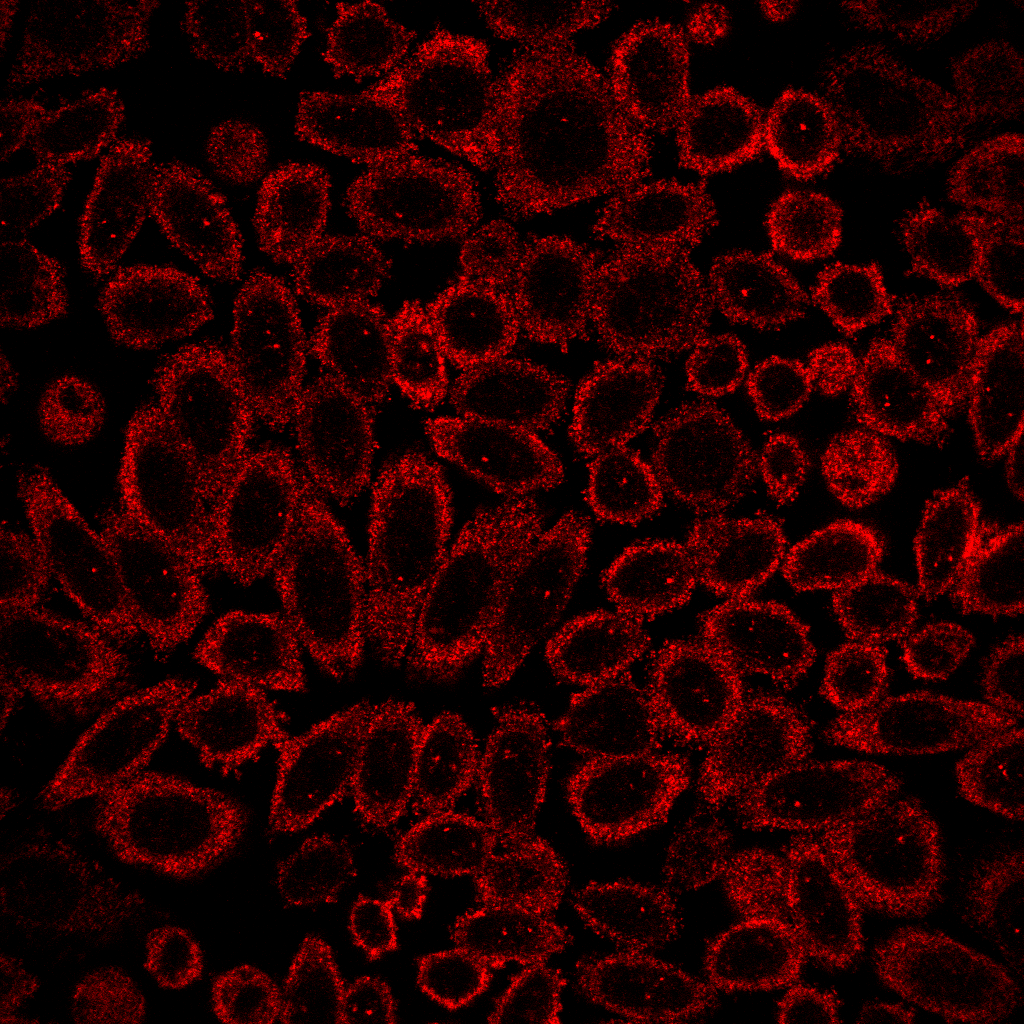

Supplement: Supplementary file 5 — Source Data Fig. 5 [file 44319_2024_98_MOESM5_ESM.zip › 5A/WT_30/wt_30min_ctrl_60x oil 2_gapdh.tif]

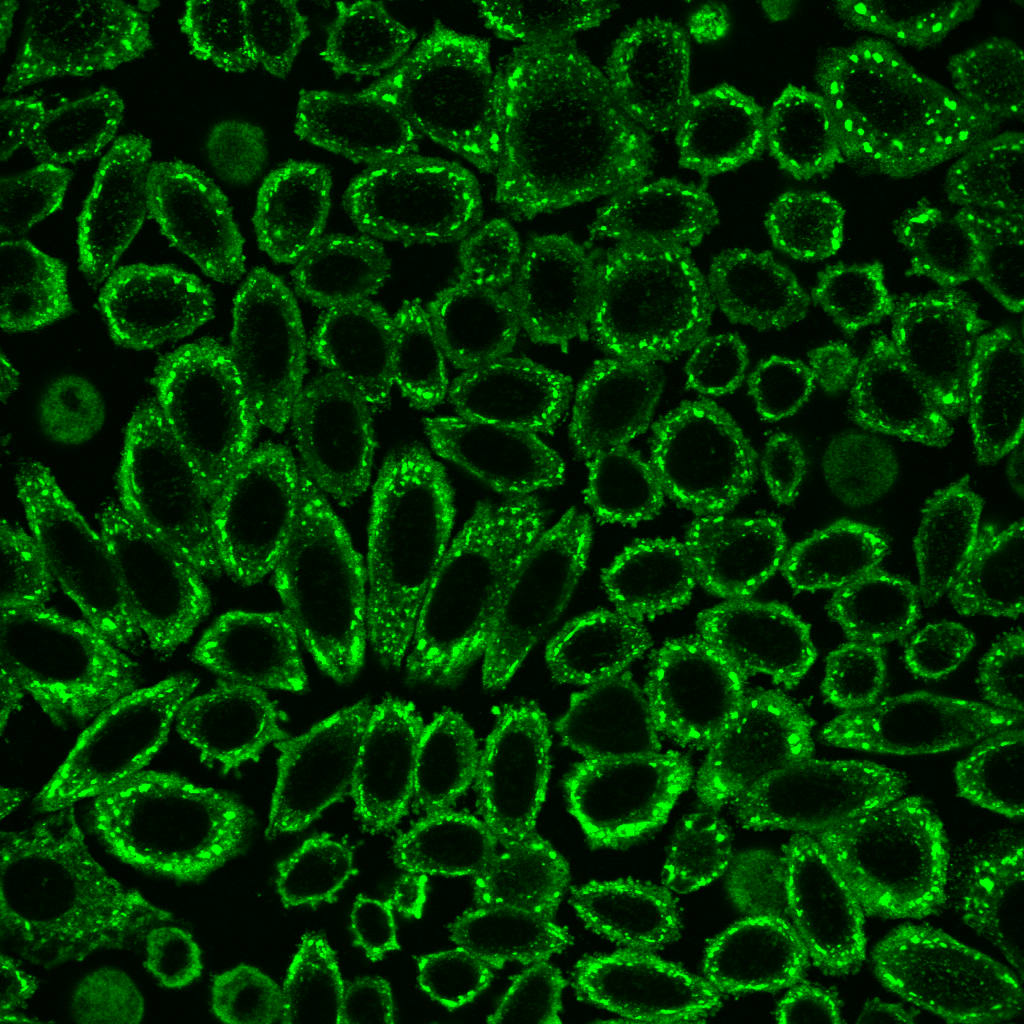

Supplement: Supplementary file 5 — Source Data Fig. 5 [file 44319_2024_98_MOESM5_ESM.zip › 5A/WT_30/wt_30min_ctrl_60x oil 2_g3bp.tif]

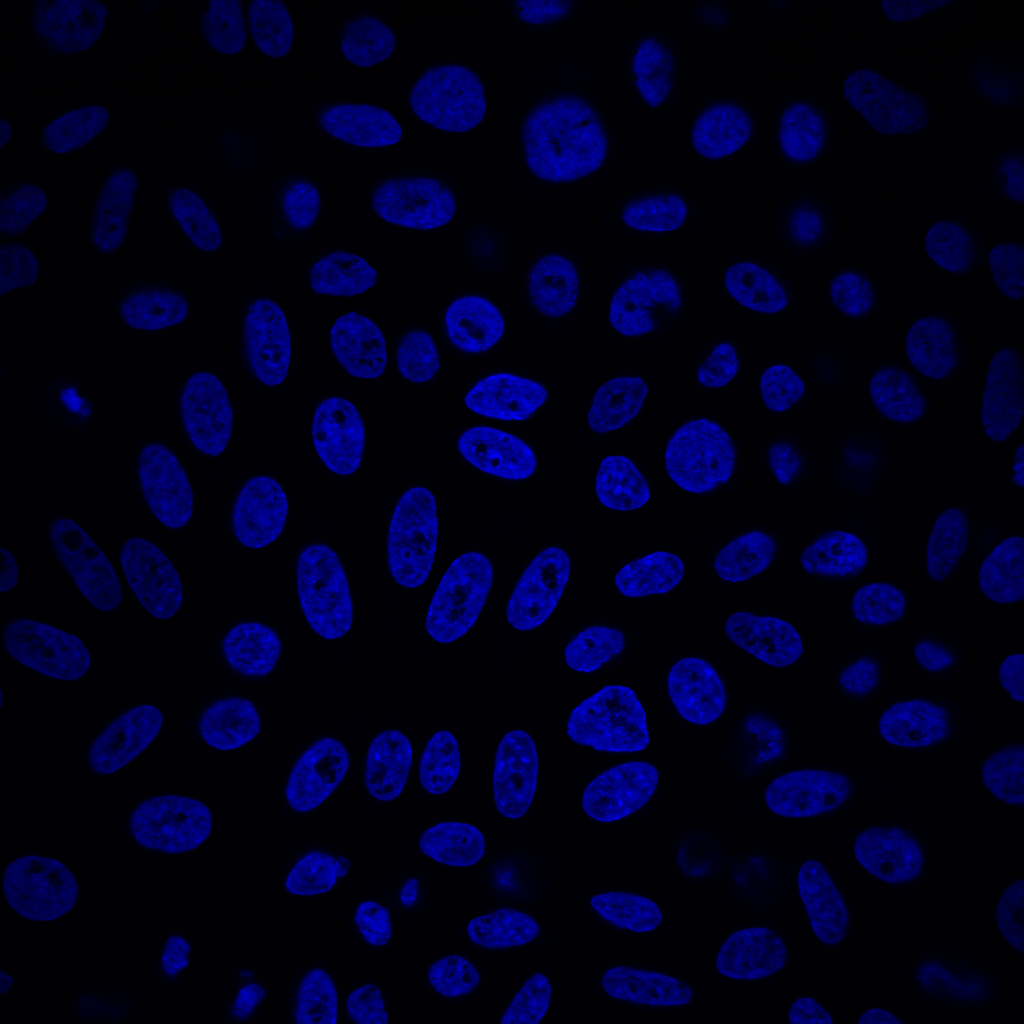

Supplement: Supplementary file 5 — Source Data Fig. 5 [file 44319_2024_98_MOESM5_ESM.zip › 5A/WT_30/wt_30min_ctrl_60x oil 2_dapi.tif]

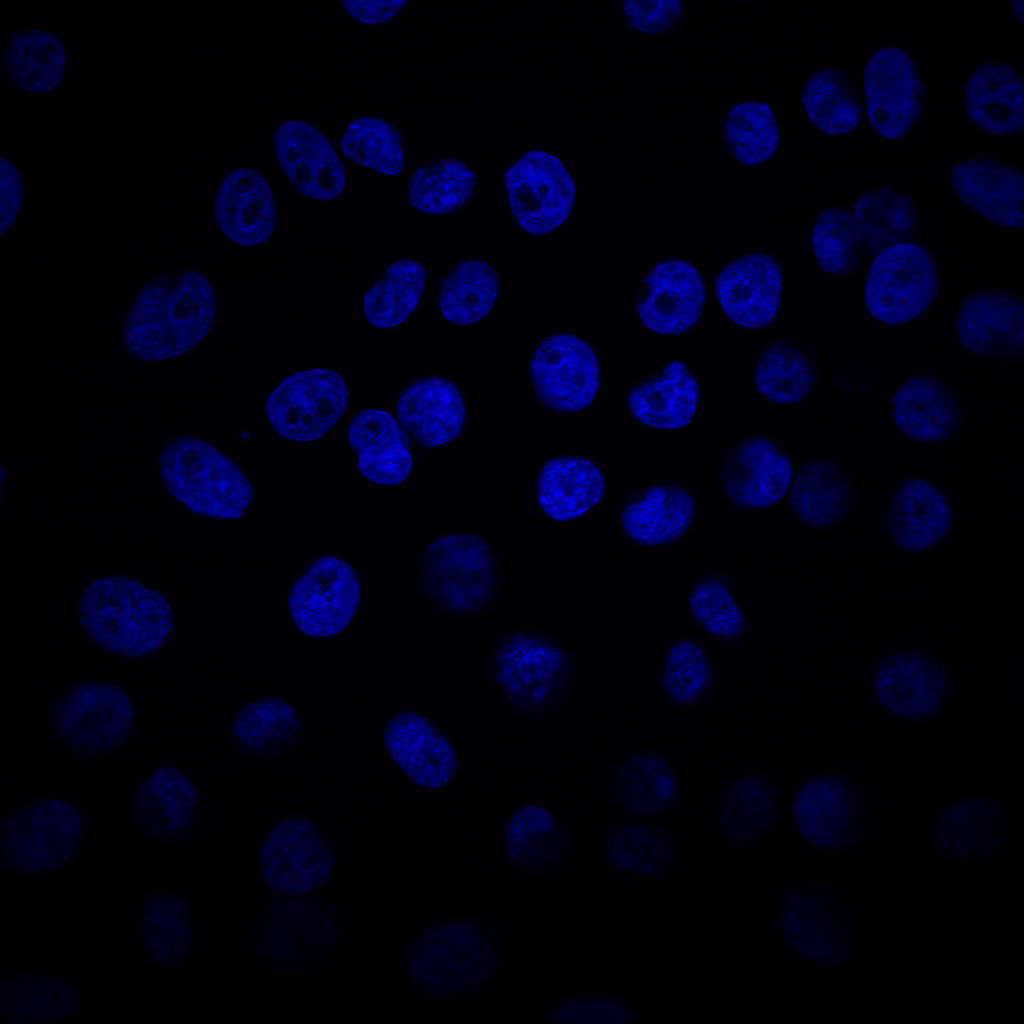

Supplement: Supplementary file 5 — Source Data Fig. 5 [file 44319_2024_98_MOESM5_ESM.zip › 5A/NAT10 KO_60/nat_60min_ctrl_60x oil 1_dapi.tif]

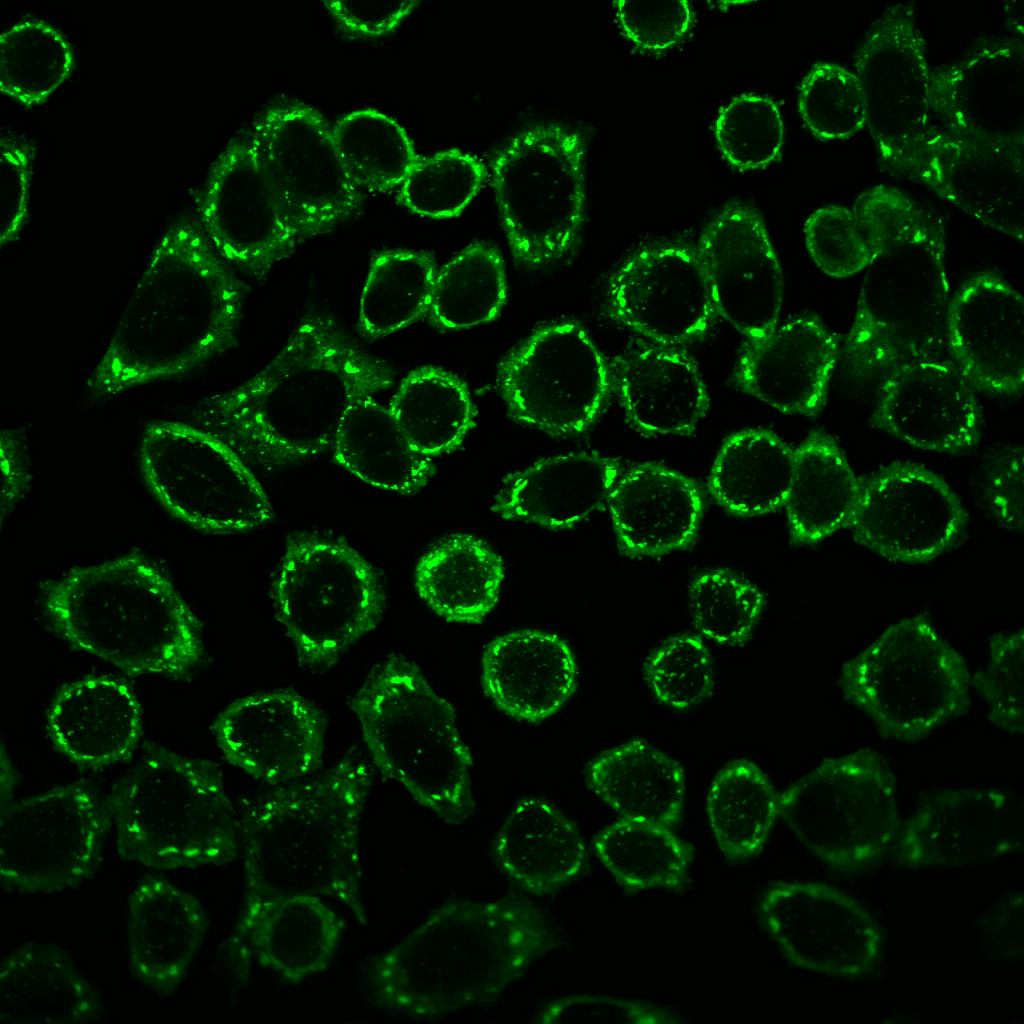

Supplement: Supplementary file 5 — Source Data Fig. 5 [file 44319_2024_98_MOESM5_ESM.zip › 5A/NAT10 KO_60/nat_60min_ctrl_60x oil 1_g3bp.tif]

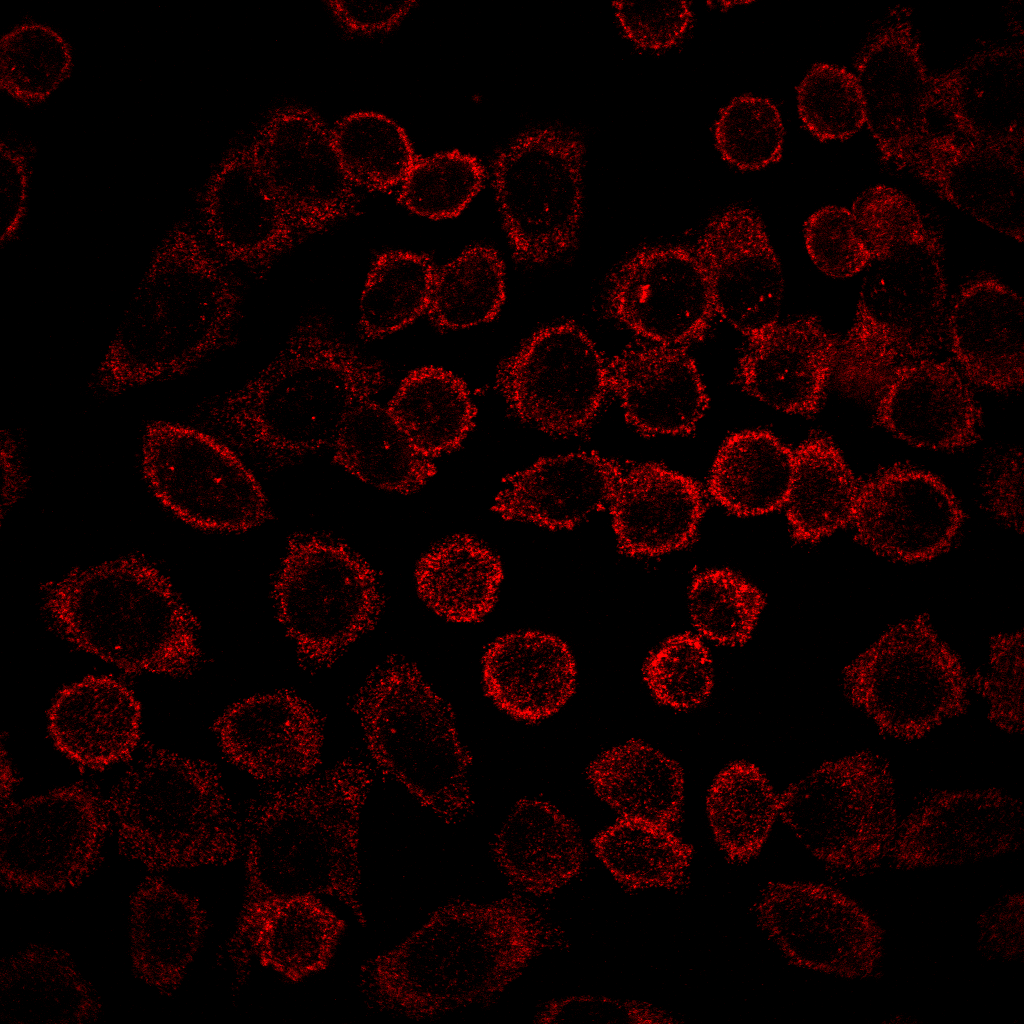

Supplement: Supplementary file 5 — Source Data Fig. 5 [file 44319_2024_98_MOESM5_ESM.zip › 5A/NAT10 KO_60/nat_60min_ctrl_60x oil 1_gapdh.tif]

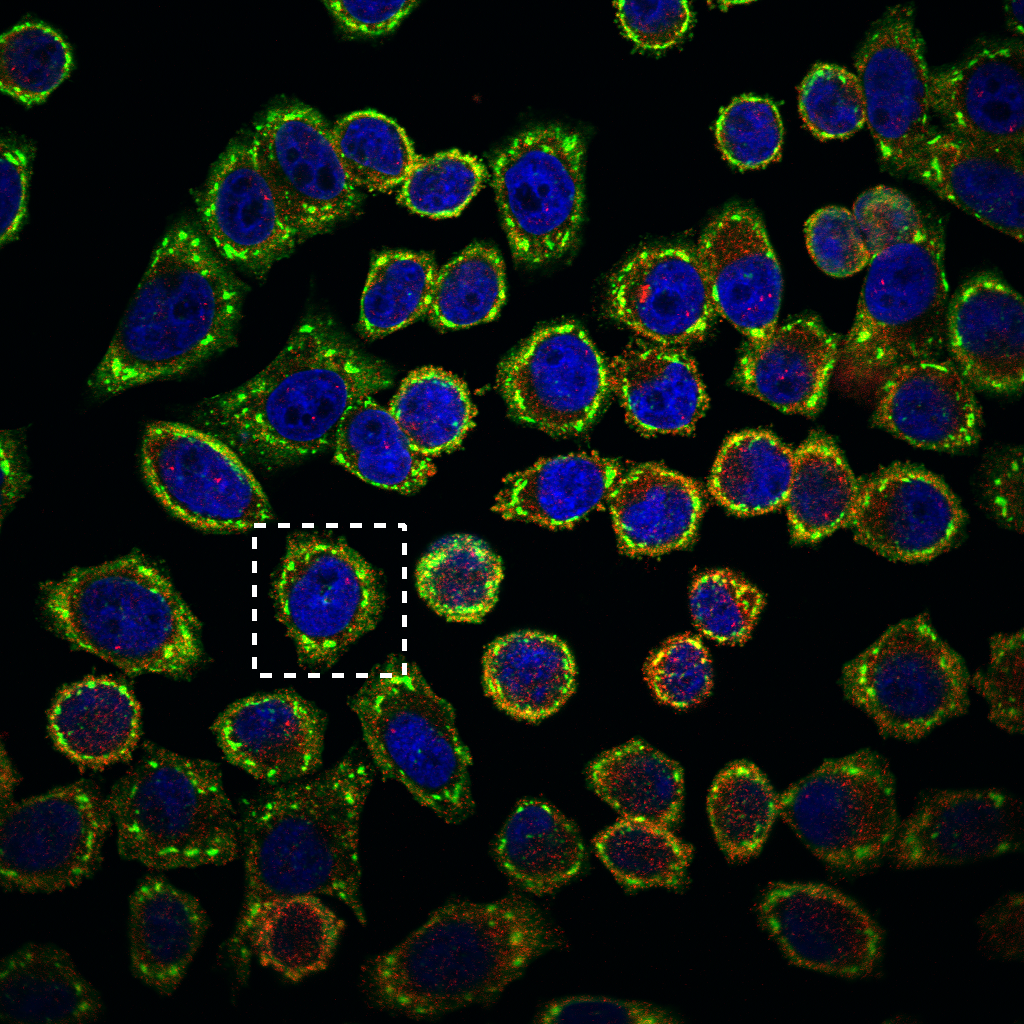

Supplement: Supplementary file 5 — Source Data Fig. 5 [file 44319_2024_98_MOESM5_ESM.zip › 5A/NAT10 KO_60/nat_60min_ctrl_60x oil 1_merged_dashed cropped area.tif]

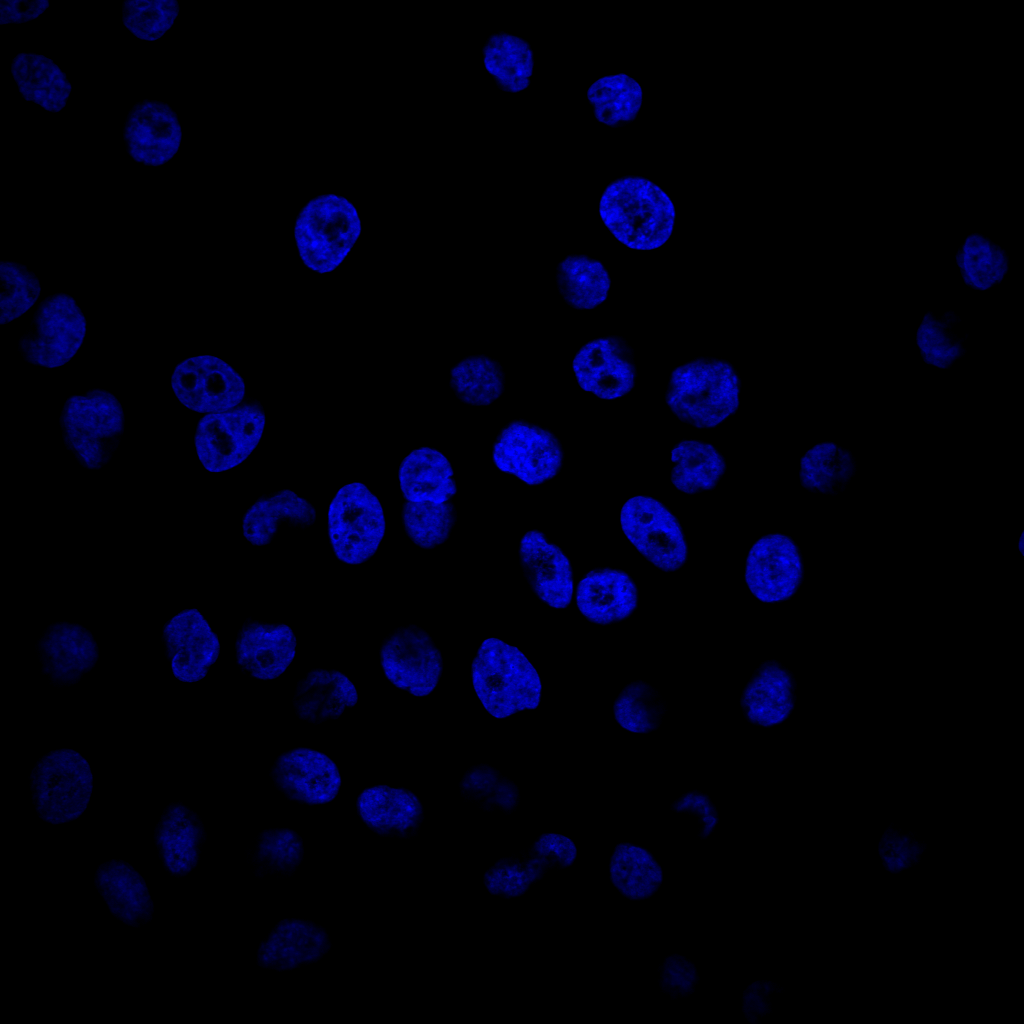

Supplement: Supplementary file 5 — Source Data Fig. 5 [file 44319_2024_98_MOESM5_ESM.zip › 5A/NAT10 KO_0/nat_ctrl_60x oil 2_dapi.tif]

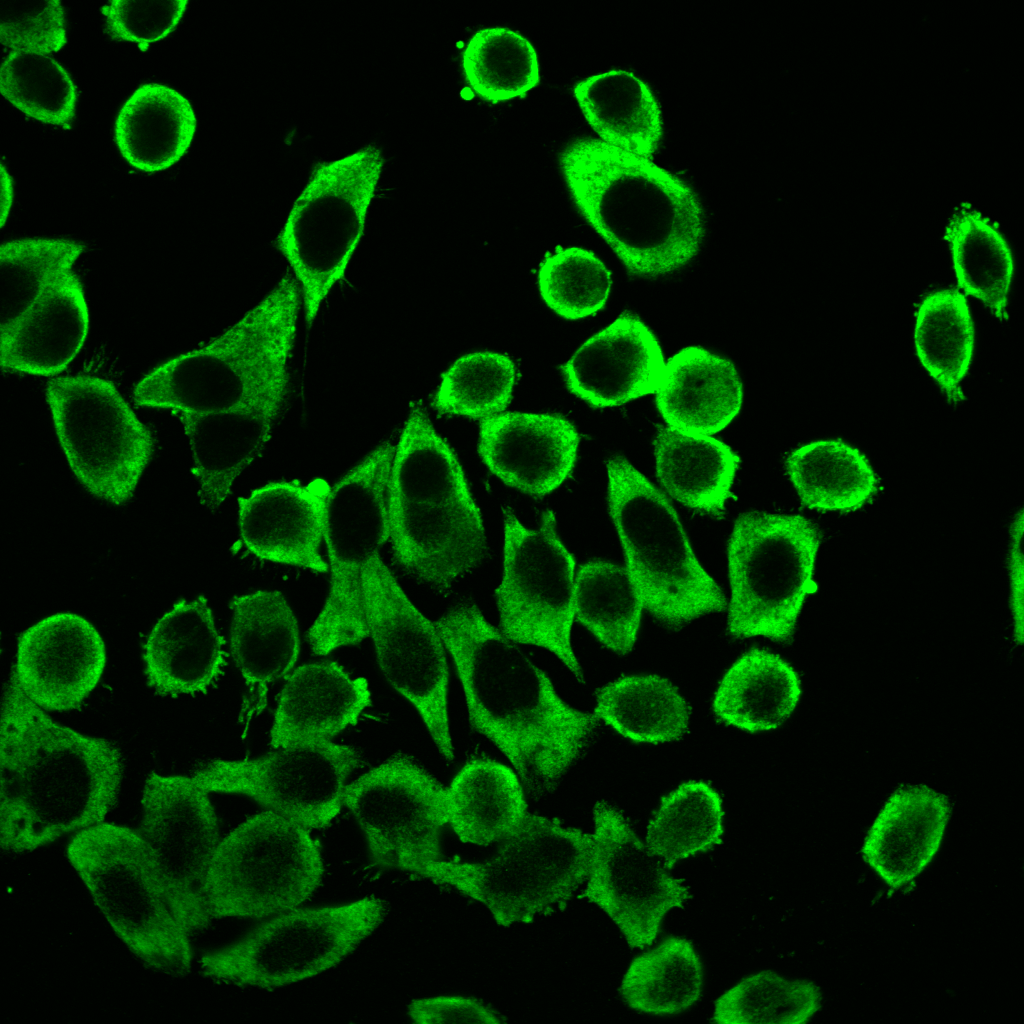

Supplement: Supplementary file 5 — Source Data Fig. 5 [file 44319_2024_98_MOESM5_ESM.zip › 5A/NAT10 KO_0/nat_ctrl_60x oil 2_g3bp.tif]

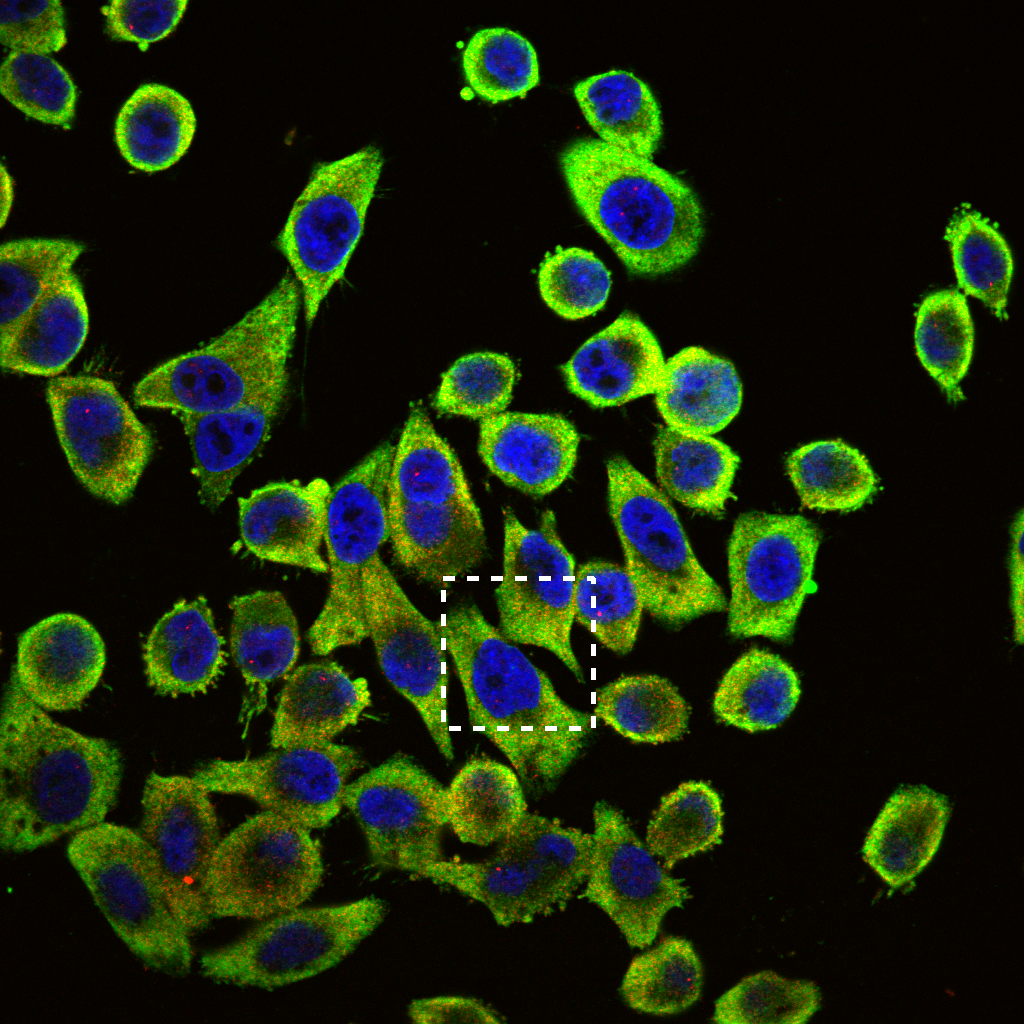

Supplement: Supplementary file 5 — Source Data Fig. 5 [file 44319_2024_98_MOESM5_ESM.zip › 5A/NAT10 KO_0/nat_ctrl_60x oil 2_merged_dashed cropped area.tif]

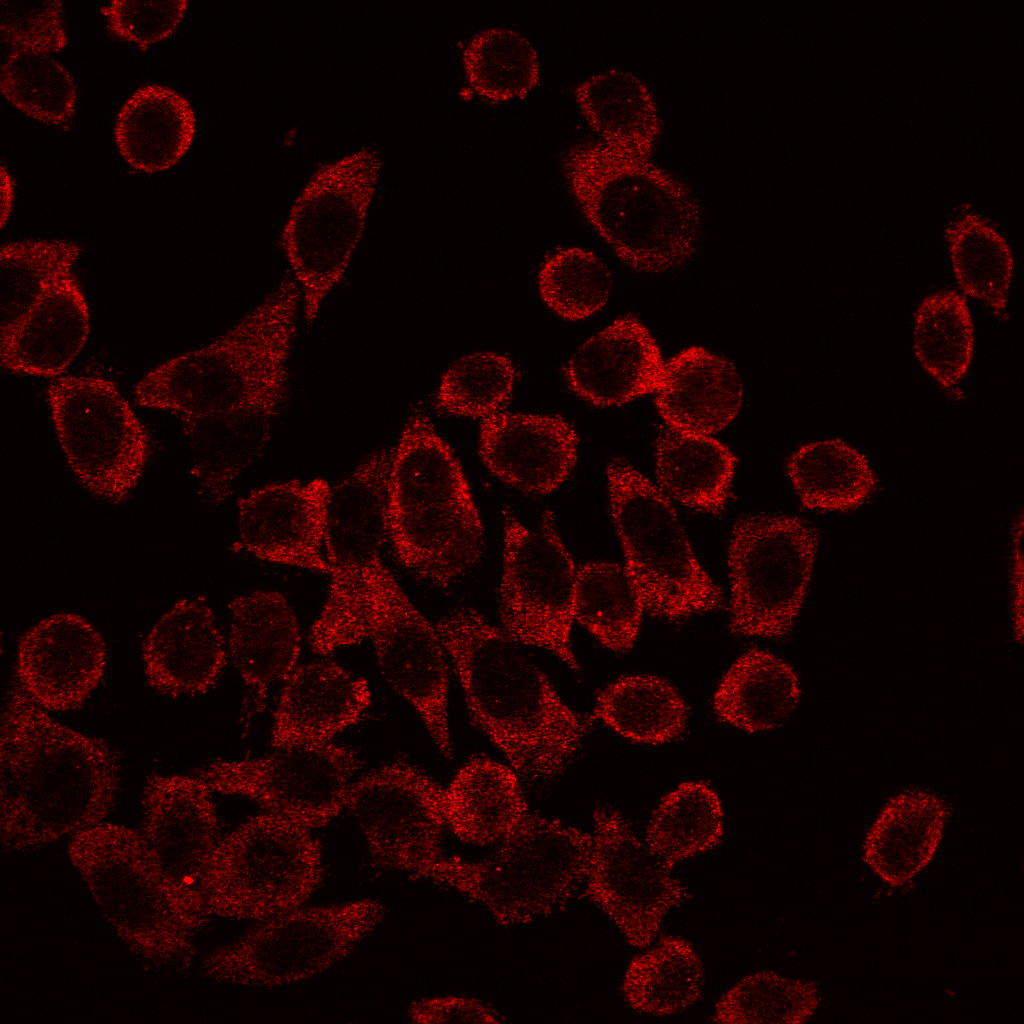

Supplement: Supplementary file 5 — Source Data Fig. 5 [file 44319_2024_98_MOESM5_ESM.zip › 5A/NAT10 KO_0/nat_ctrl_60x oil 2_gapdh.tif]

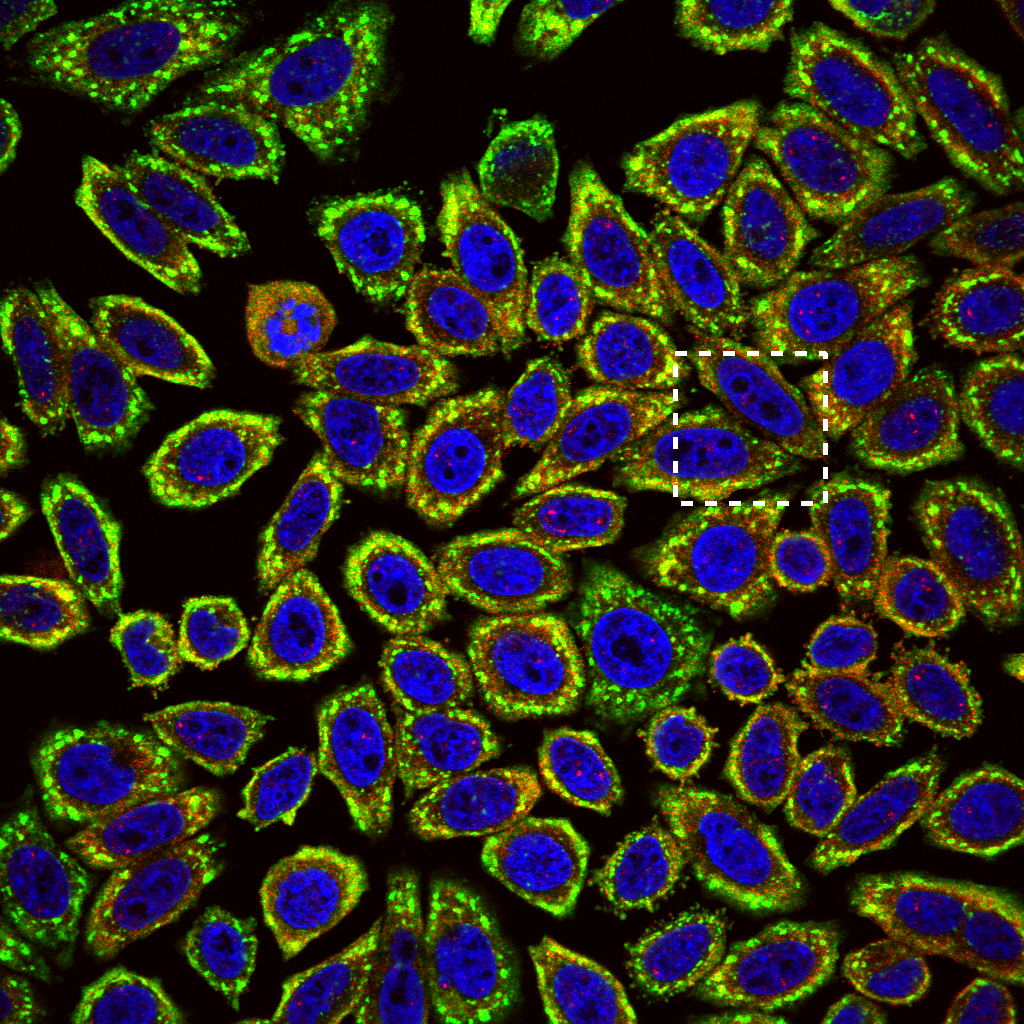

Supplement: Supplementary file 5 — Source Data Fig. 5 [file 44319_2024_98_MOESM5_ESM.zip › 5A/WT_60/wt_60min_ctrl_60x oil 1_merged_dashed cropped area.tif]

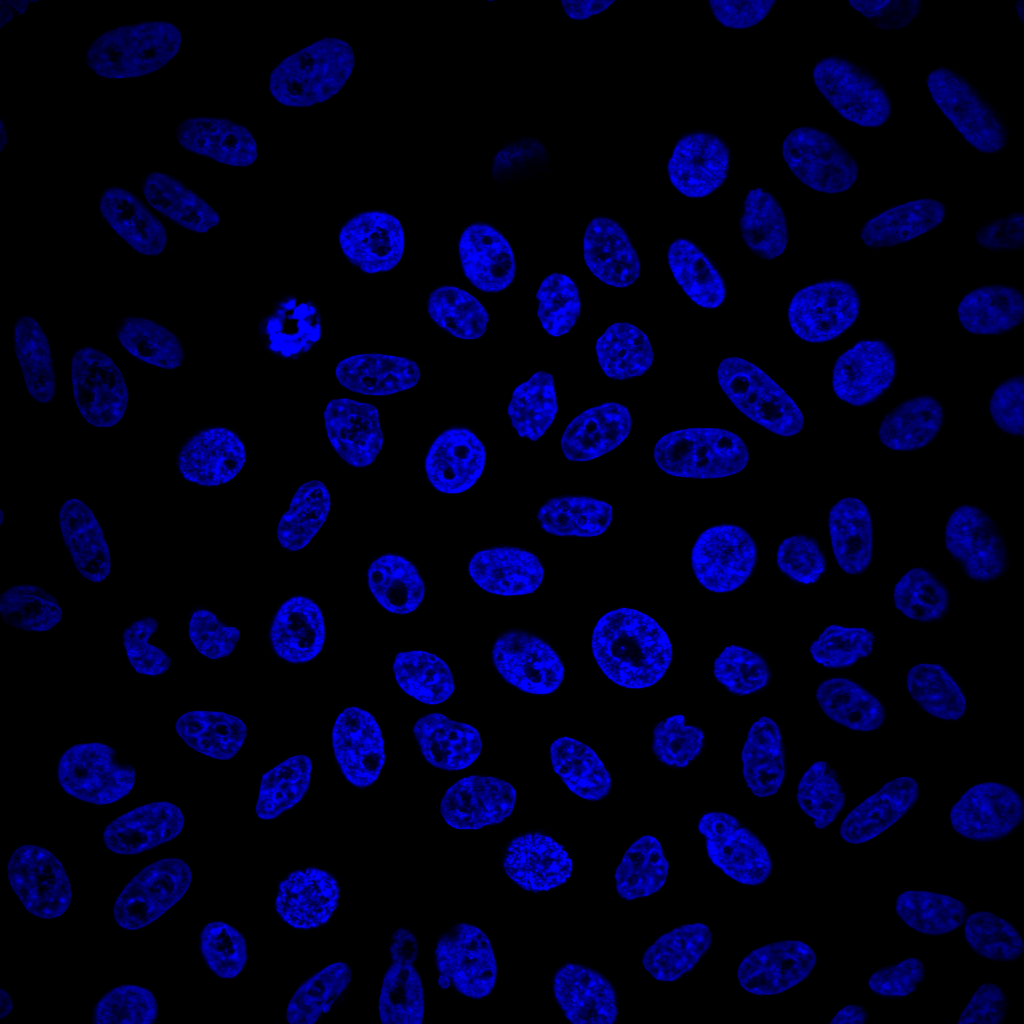

Supplement: Supplementary file 5 — Source Data Fig. 5 [file 44319_2024_98_MOESM5_ESM.zip › 5A/WT_60/wt_60min_ctrl_60x oil 1_dapi.tif]

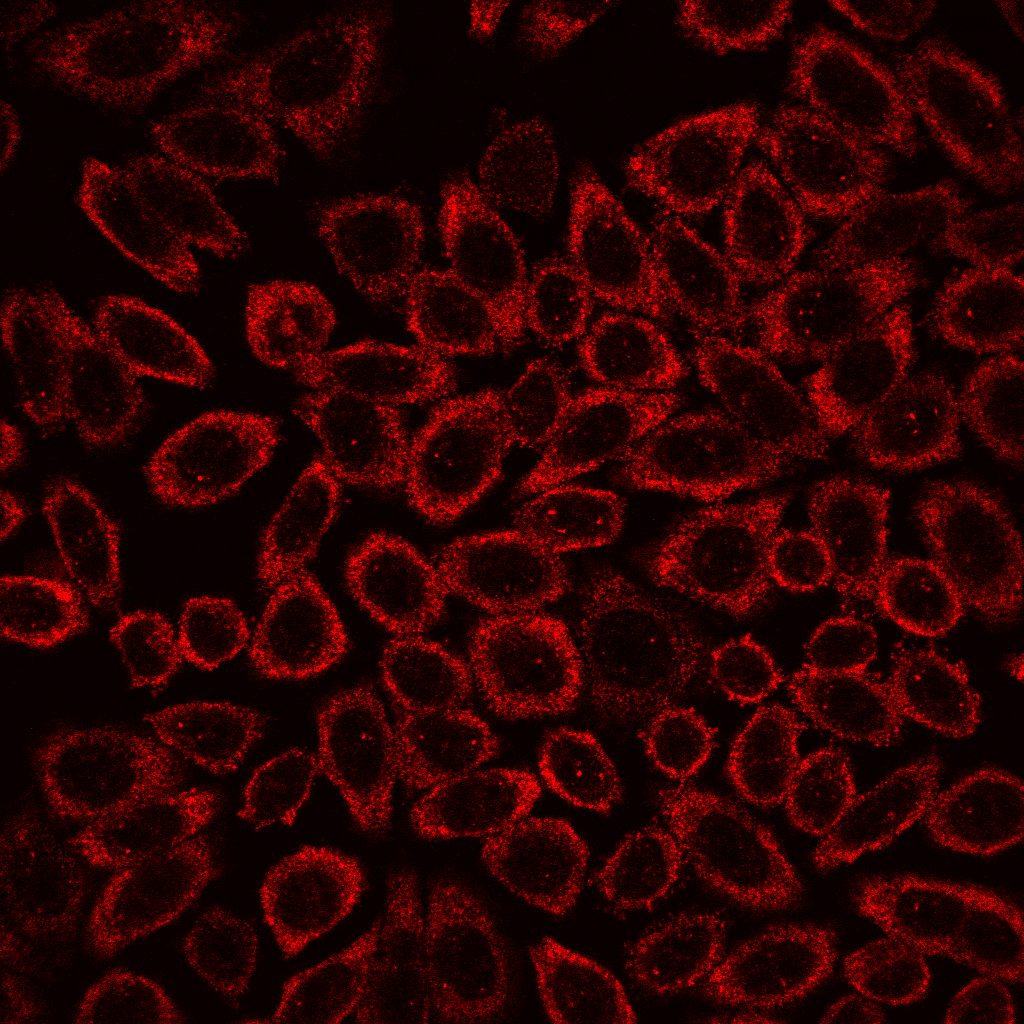

Supplement: Supplementary file 5 — Source Data Fig. 5 [file 44319_2024_98_MOESM5_ESM.zip › 5A/WT_60/wt_60min_ctrl_60x oil 1_gapdh.tif]

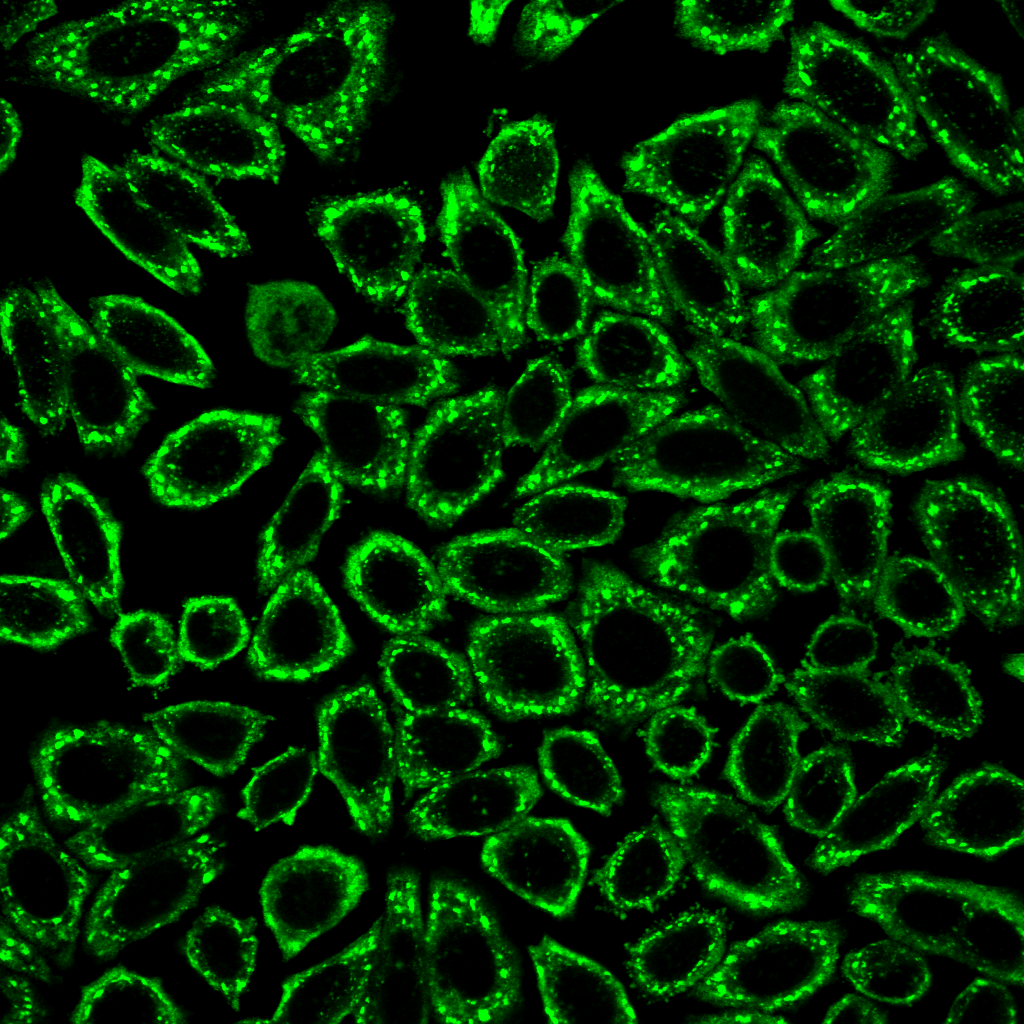

Supplement: Supplementary file 5 — Source Data Fig. 5 [file 44319_2024_98_MOESM5_ESM.zip › 5A/WT_60/wt_60min_ctrl_60x oil 1_g3bp.tif]

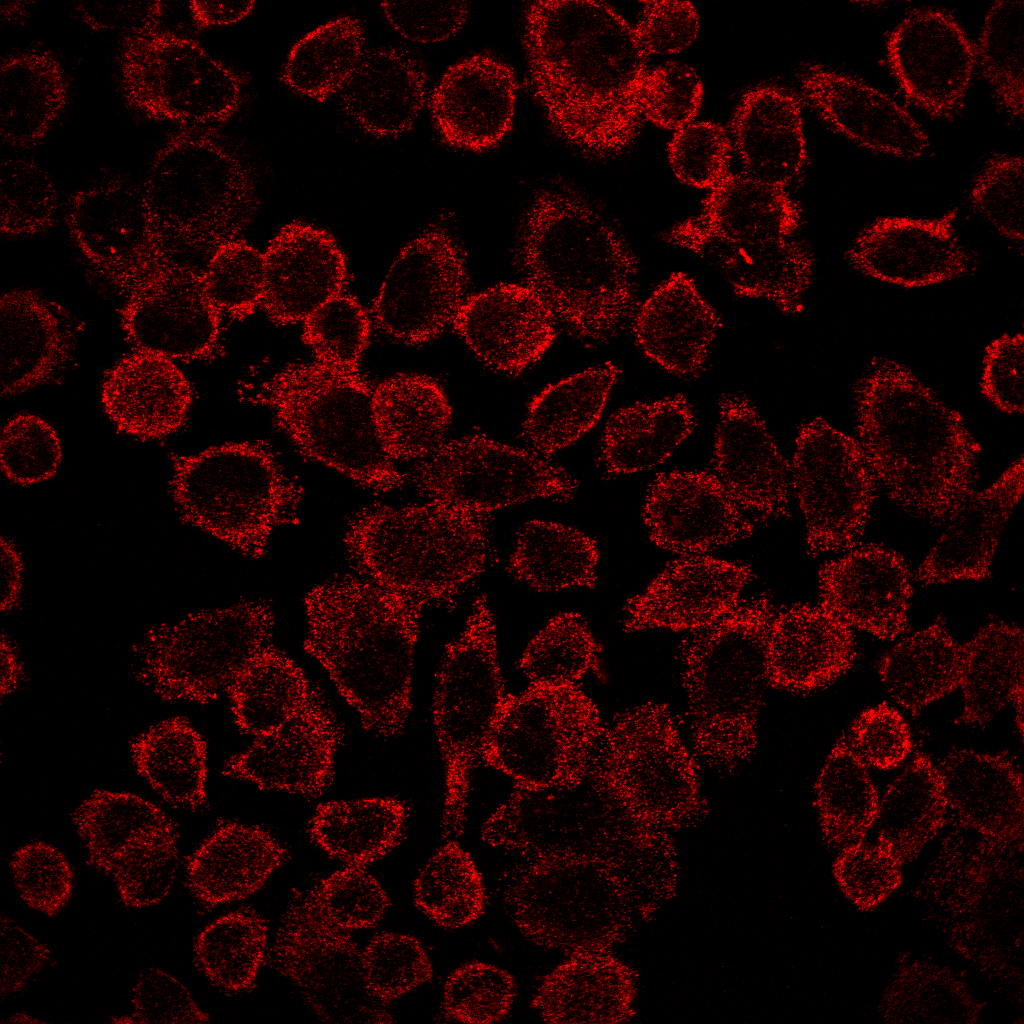

Supplement: Supplementary file 5 — Source Data Fig. 5 [file 44319_2024_98_MOESM5_ESM.zip › 5A/NAT10 KO_30/nat_30min_ctrl_60x oil 3_gapdh.tif]

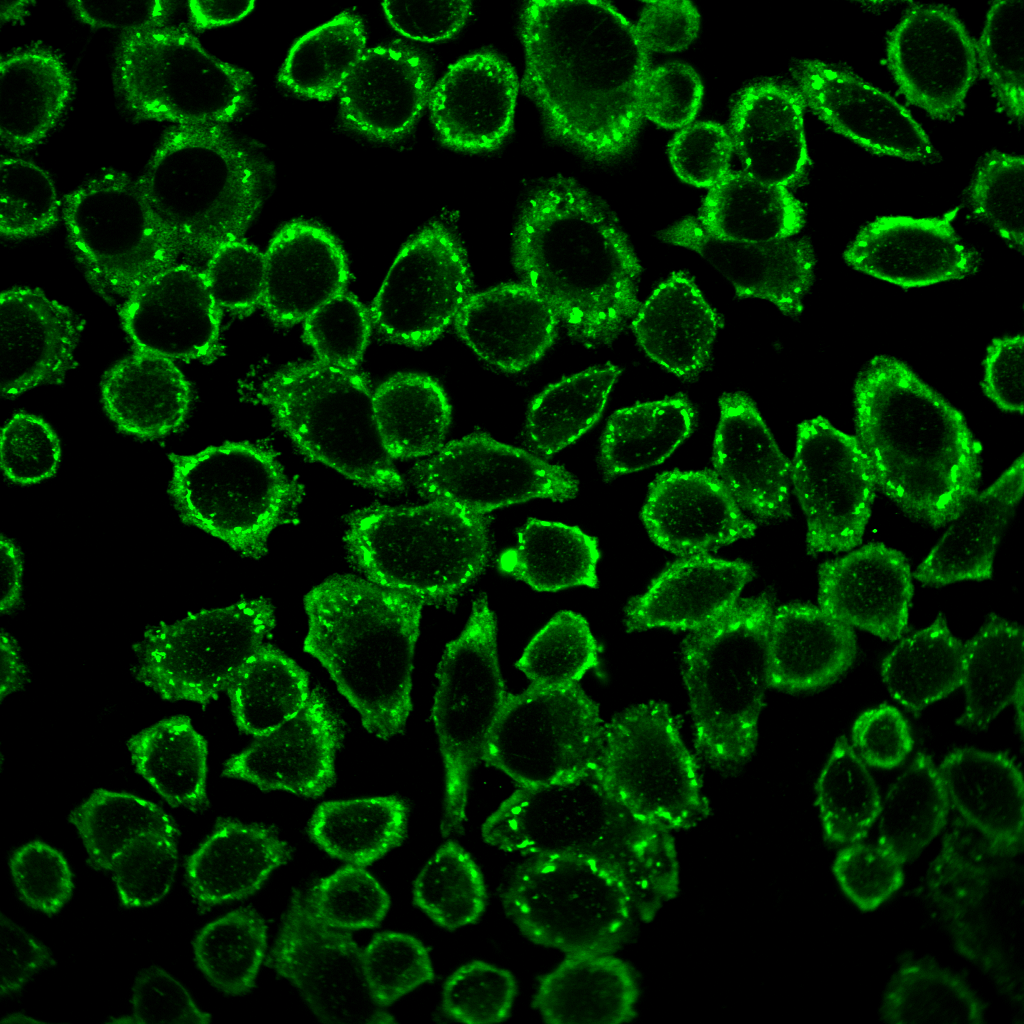

Supplement: Supplementary file 5 — Source Data Fig. 5 [file 44319_2024_98_MOESM5_ESM.zip › 5A/NAT10 KO_30/nat_30min_ctrl_60x oil 3_g3bp.tif]

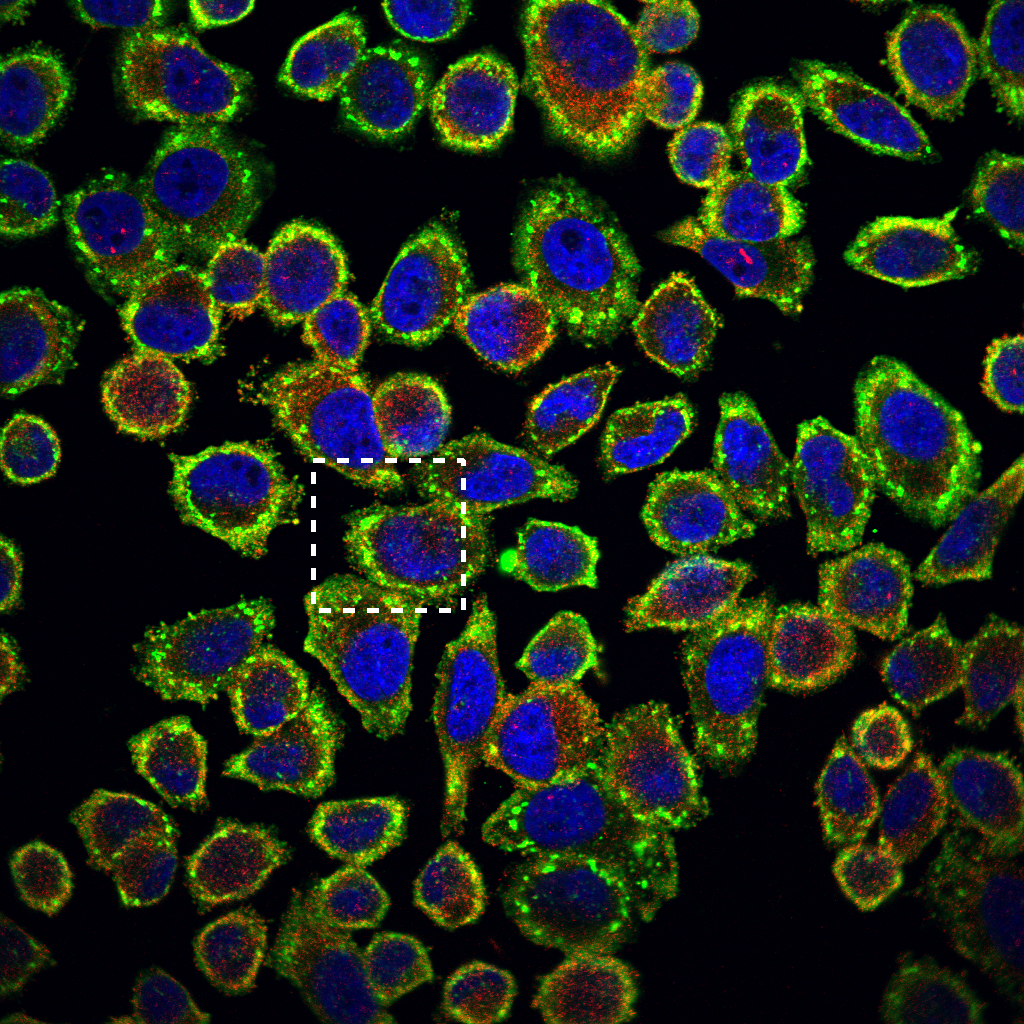

Supplement: Supplementary file 5 — Source Data Fig. 5 [file 44319_2024_98_MOESM5_ESM.zip › 5A/NAT10 KO_30/nat_30min_ctrl_60x oil 3_merged_dashed cropped area.tif]

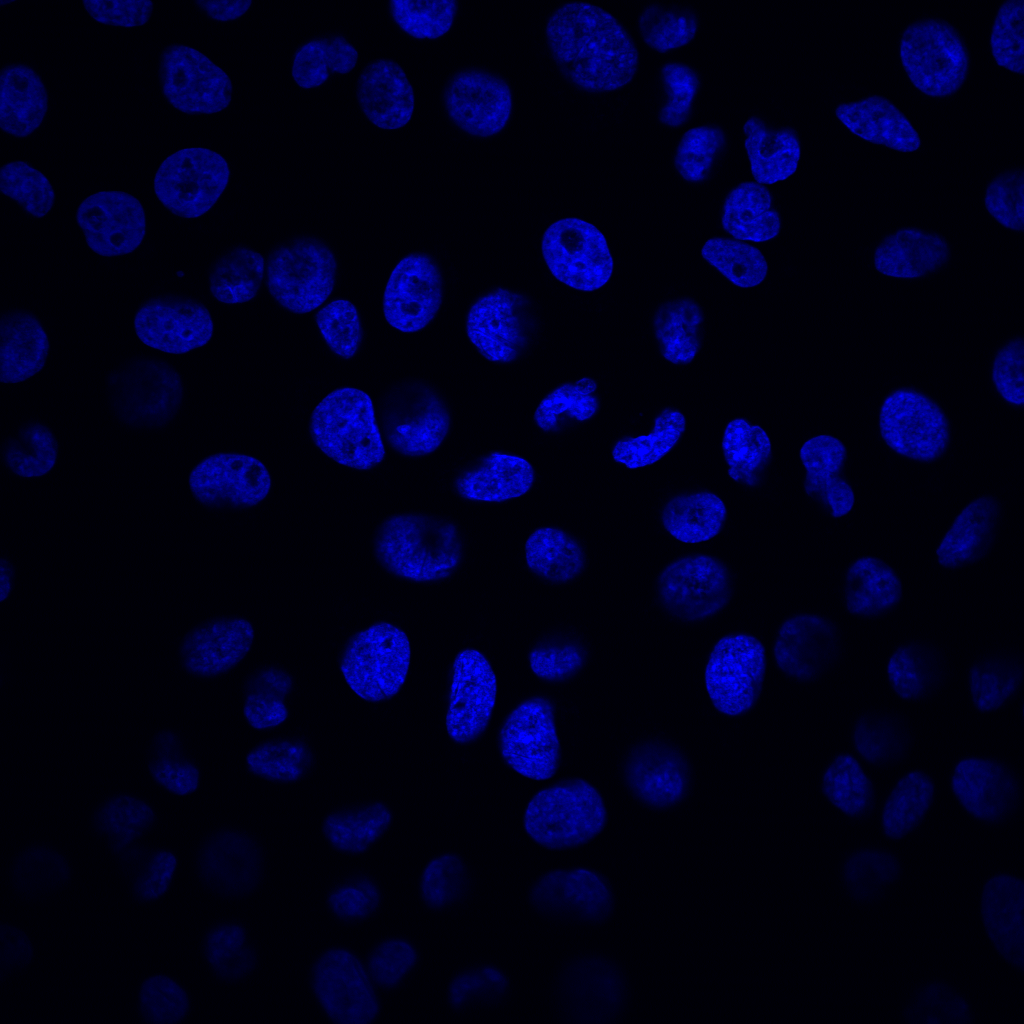

Supplement: Supplementary file 5 — Source Data Fig. 5 [file 44319_2024_98_MOESM5_ESM.zip › 5A/NAT10 KO_30/nat_30min_ctrl_60x oil 3_dapi.tif]

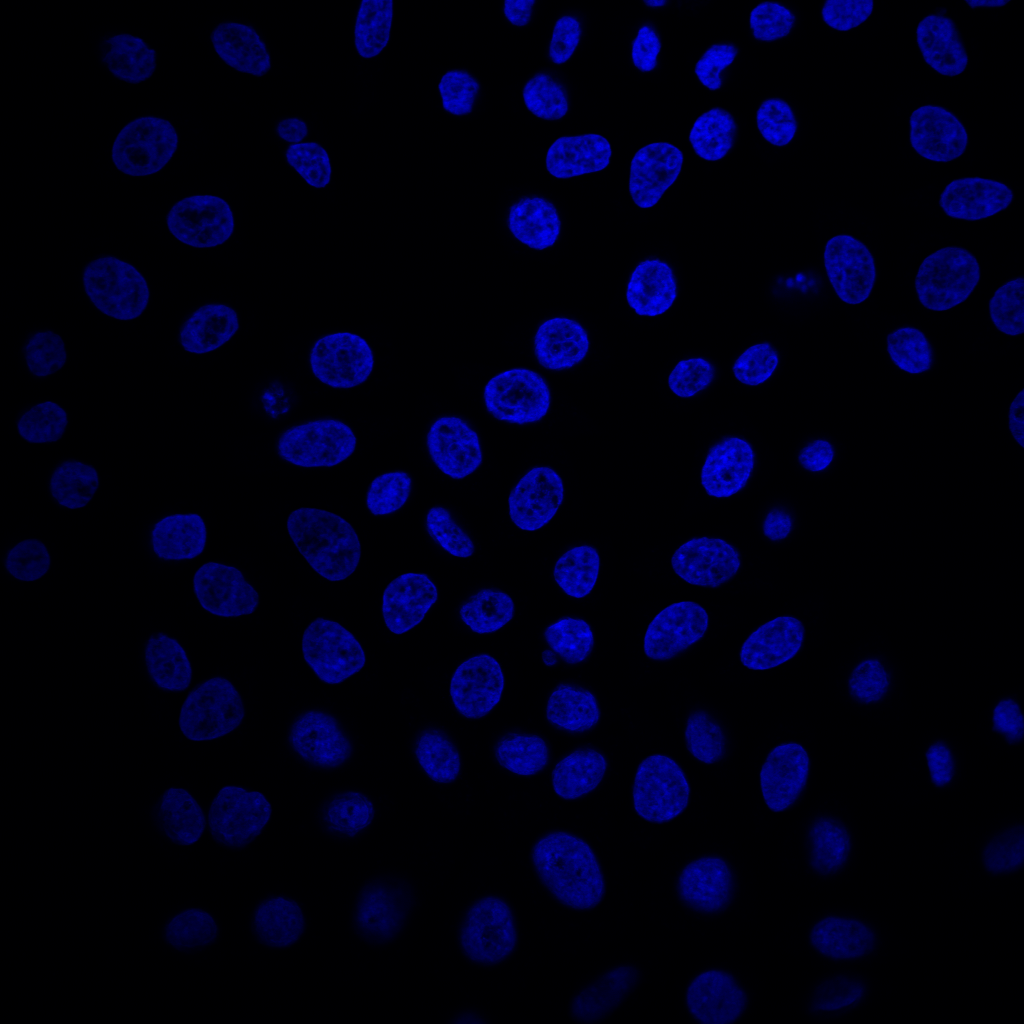

Supplement: Supplementary file 5 — Source Data Fig. 5 [file 44319_2024_98_MOESM5_ESM.zip › 5B/WT_0/wt_ctrl_60x oil 1_dapi.tif]

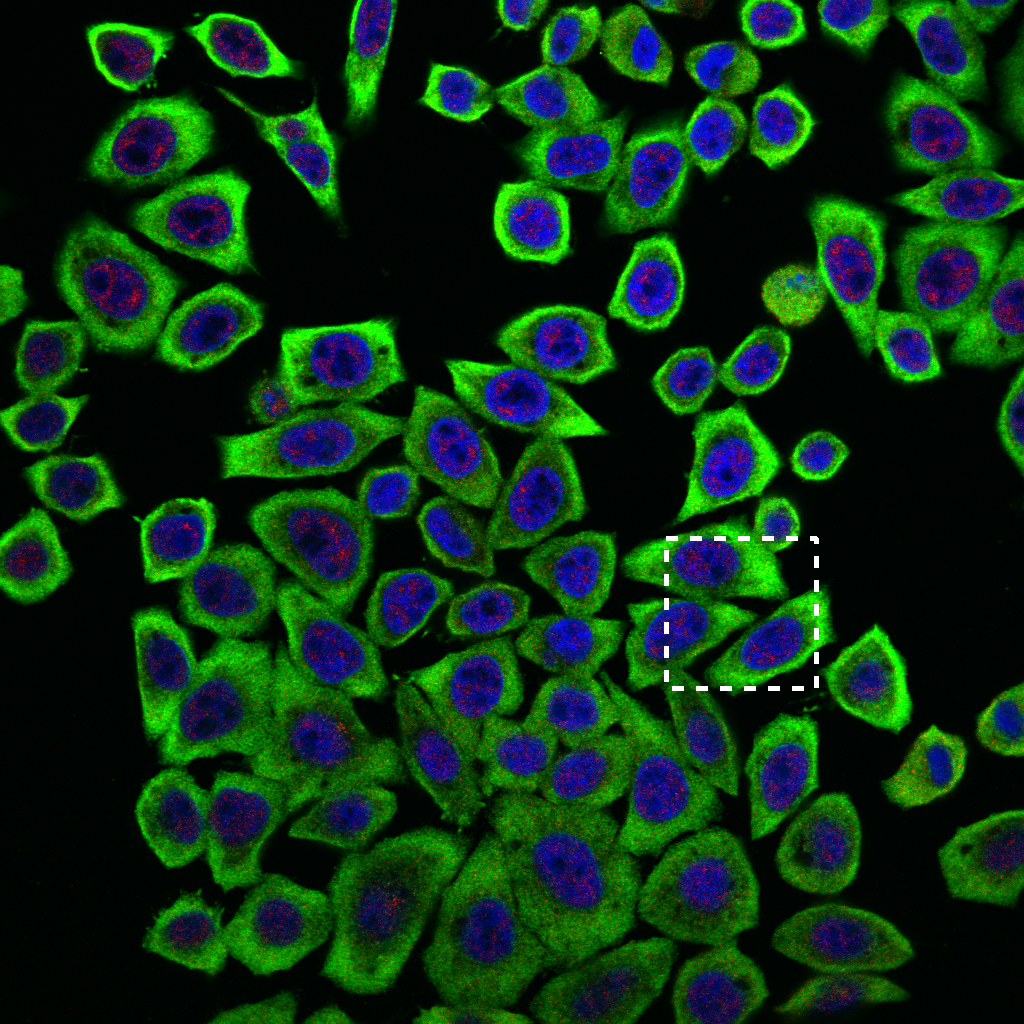

Supplement: Supplementary file 5 — Source Data Fig. 5 [file 44319_2024_98_MOESM5_ESM.zip › 5B/WT_0/wt_ctrl_60x oil 1_merged_dashed cropped area.tif]

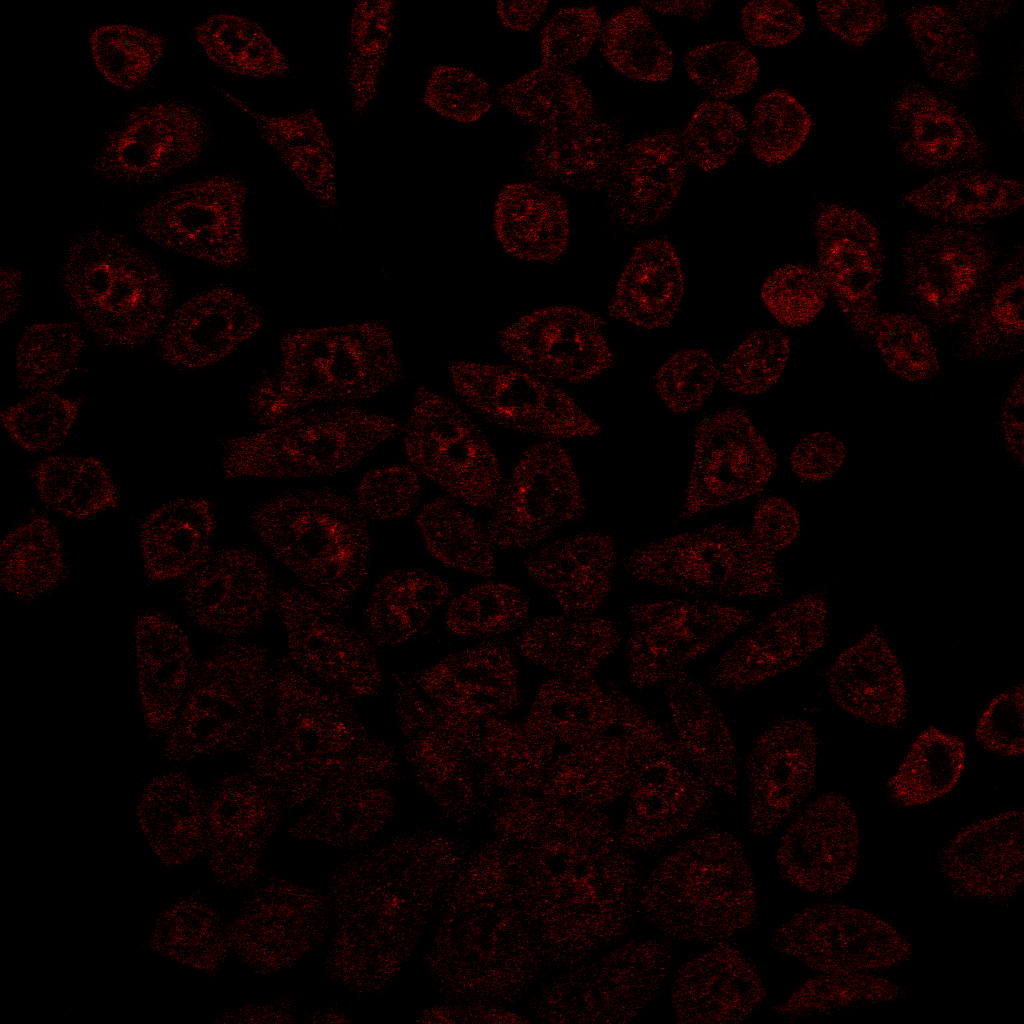

Supplement: Supplementary file 5 — Source Data Fig. 5 [file 44319_2024_98_MOESM5_ESM.zip › 5B/WT_0/wt_ctrl_60x oil 1_ahnak.tif]

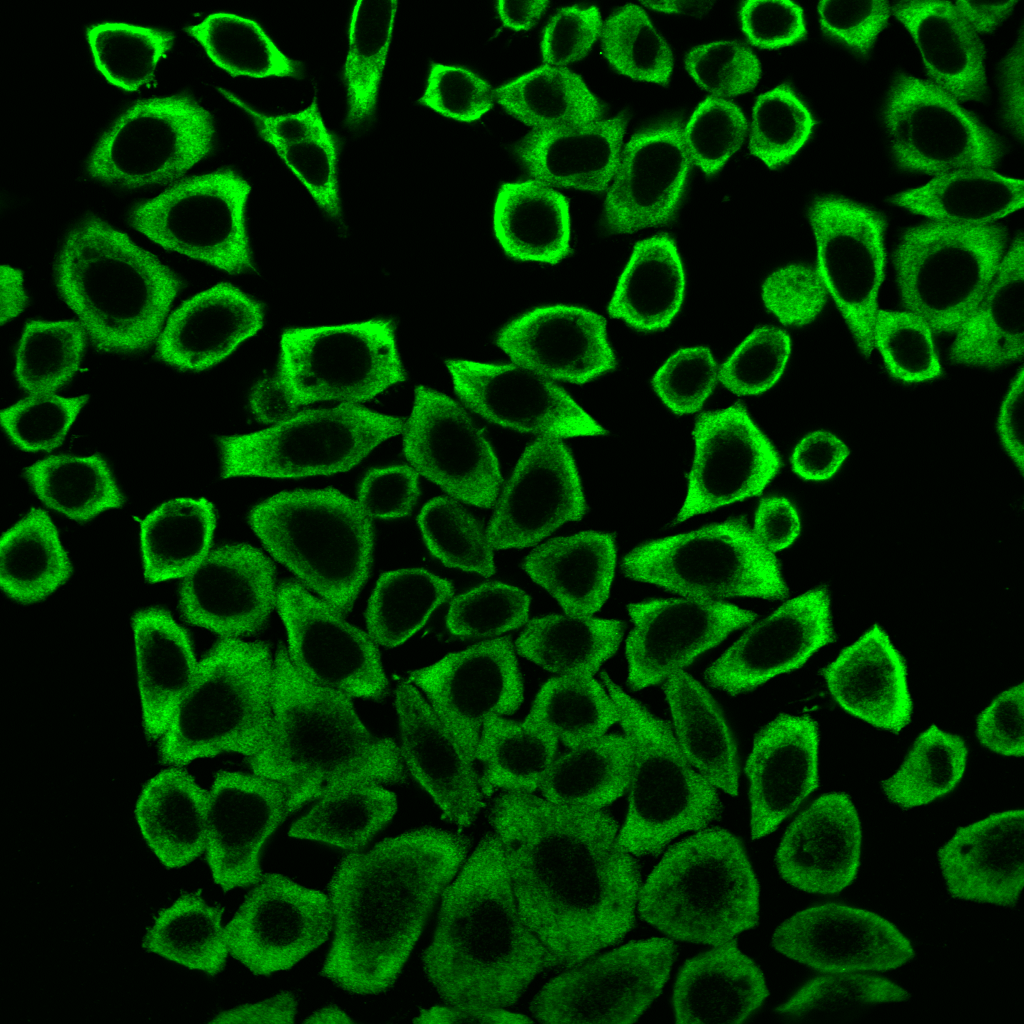

Supplement: Supplementary file 5 — Source Data Fig. 5 [file 44319_2024_98_MOESM5_ESM.zip › 5B/WT_0/wt_ctrl_60x oil 1_g3bp.tif]

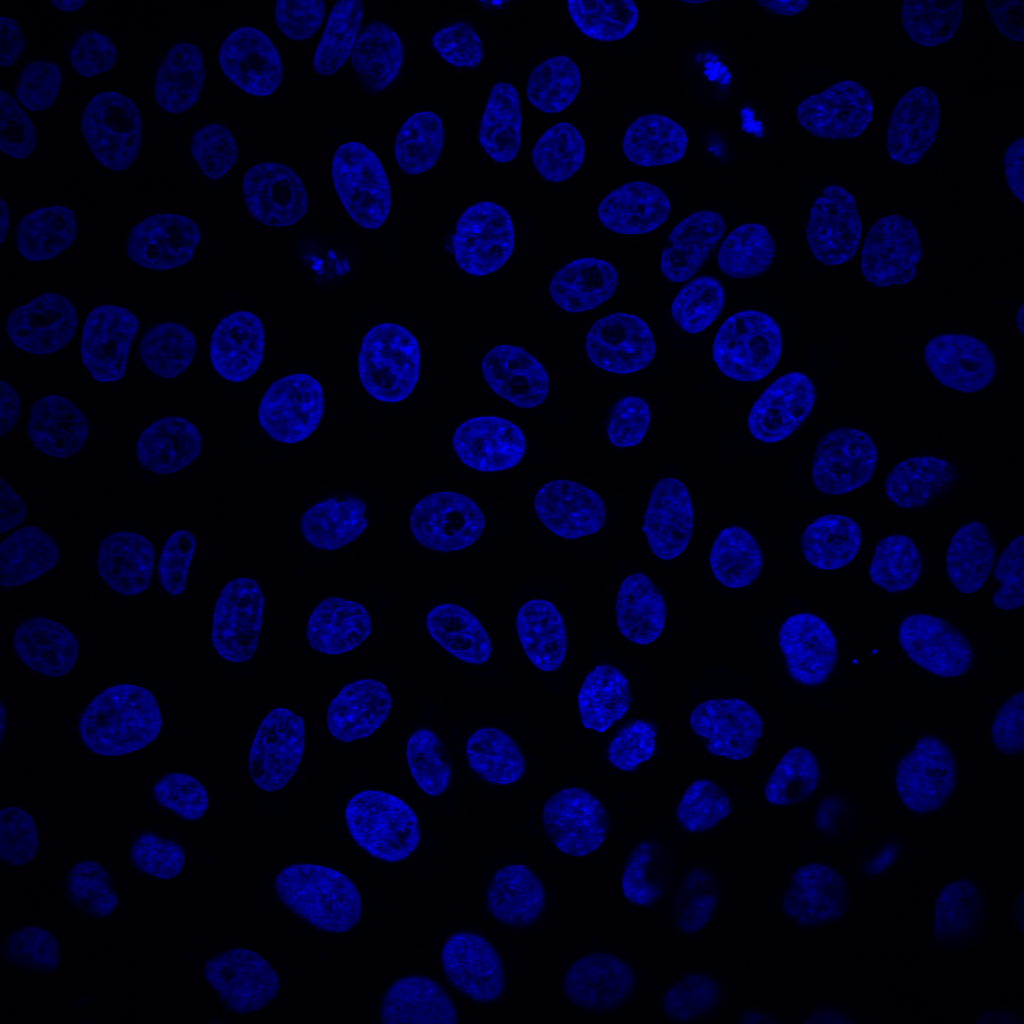

Supplement: Supplementary file 5 — Source Data Fig. 5 [file 44319_2024_98_MOESM5_ESM.zip › 5B/WT_30/wt_30 min_ctrl_60x oil 2_dapi.tif]

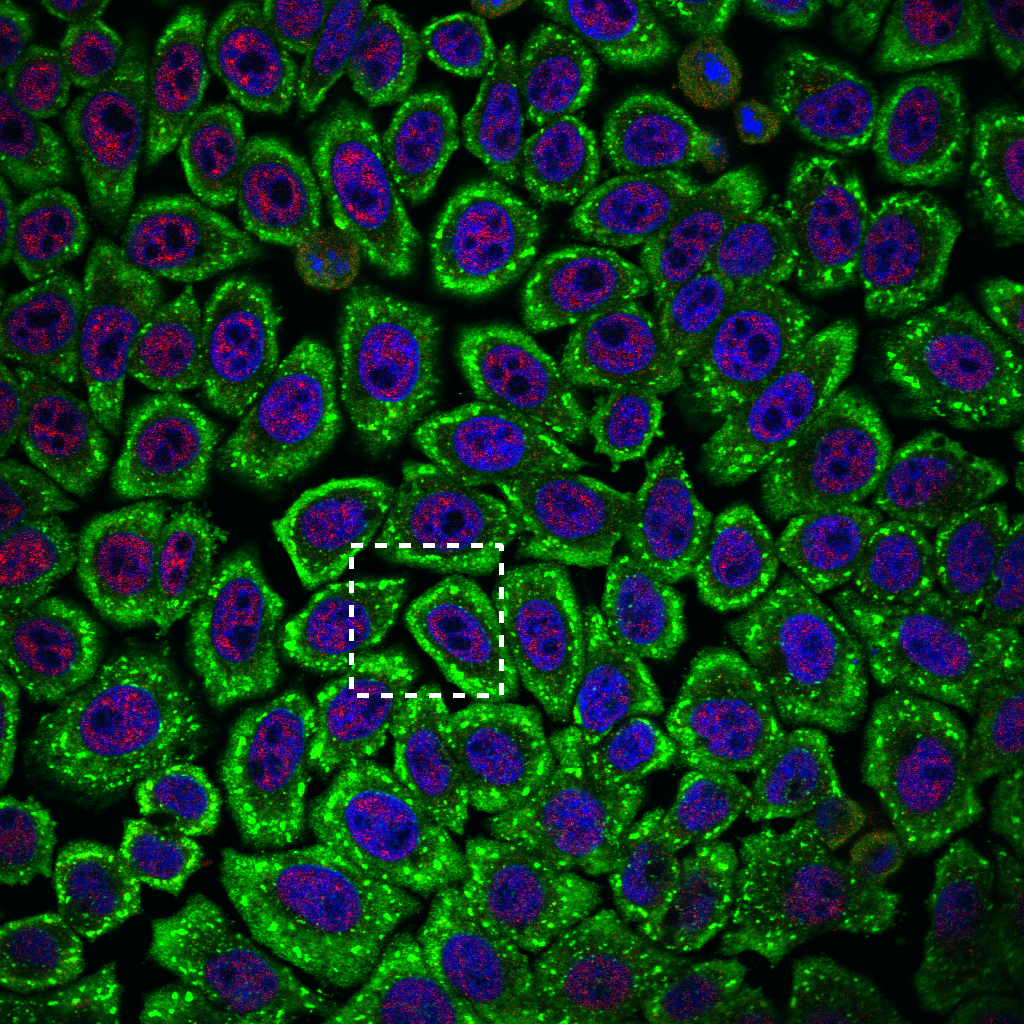

Supplement: Supplementary file 5 — Source Data Fig. 5 [file 44319_2024_98_MOESM5_ESM.zip › 5B/WT_30/wt_30 min_ctrl_60x oil 2_merged_dashed cropped area.tif]

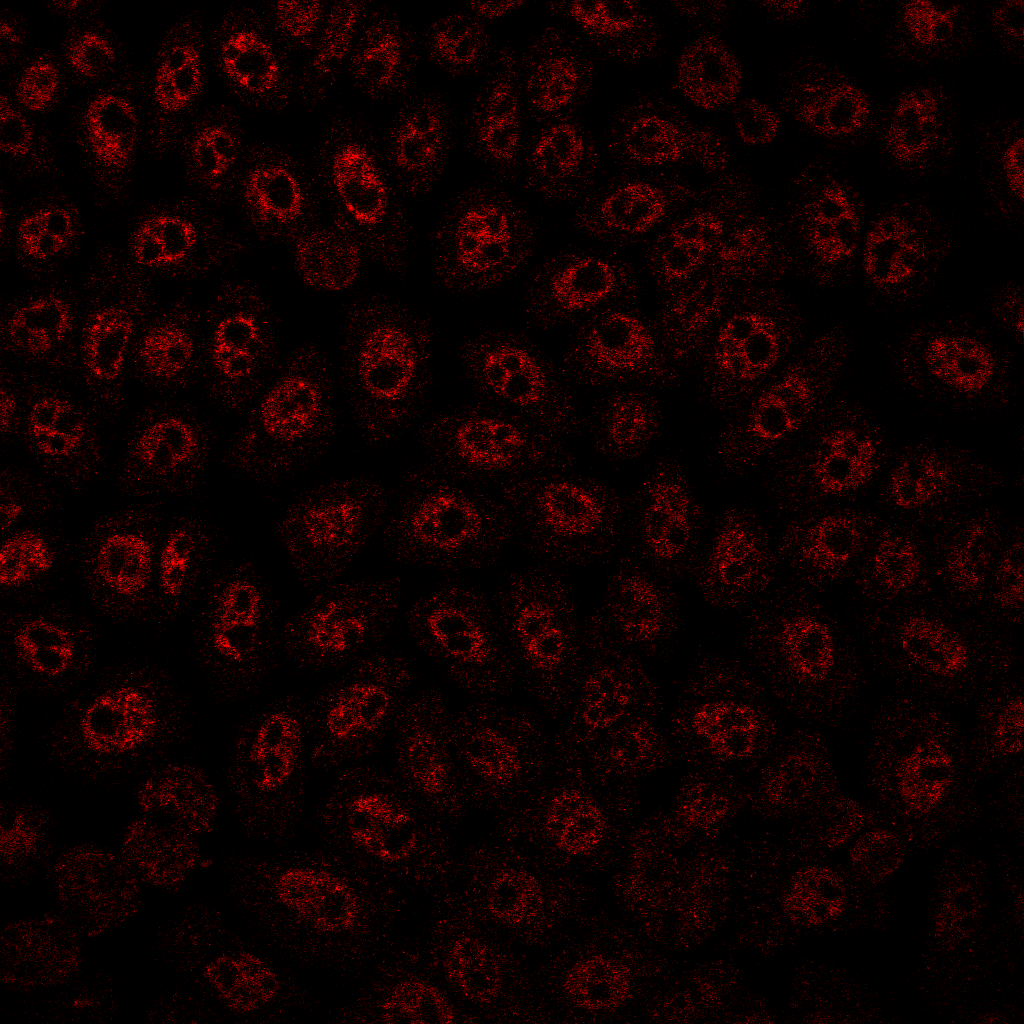

Supplement: Supplementary file 5 — Source Data Fig. 5 [file 44319_2024_98_MOESM5_ESM.zip › 5B/WT_30/wt_30 min_ctrl_60x oil 2_ahnak.tif]

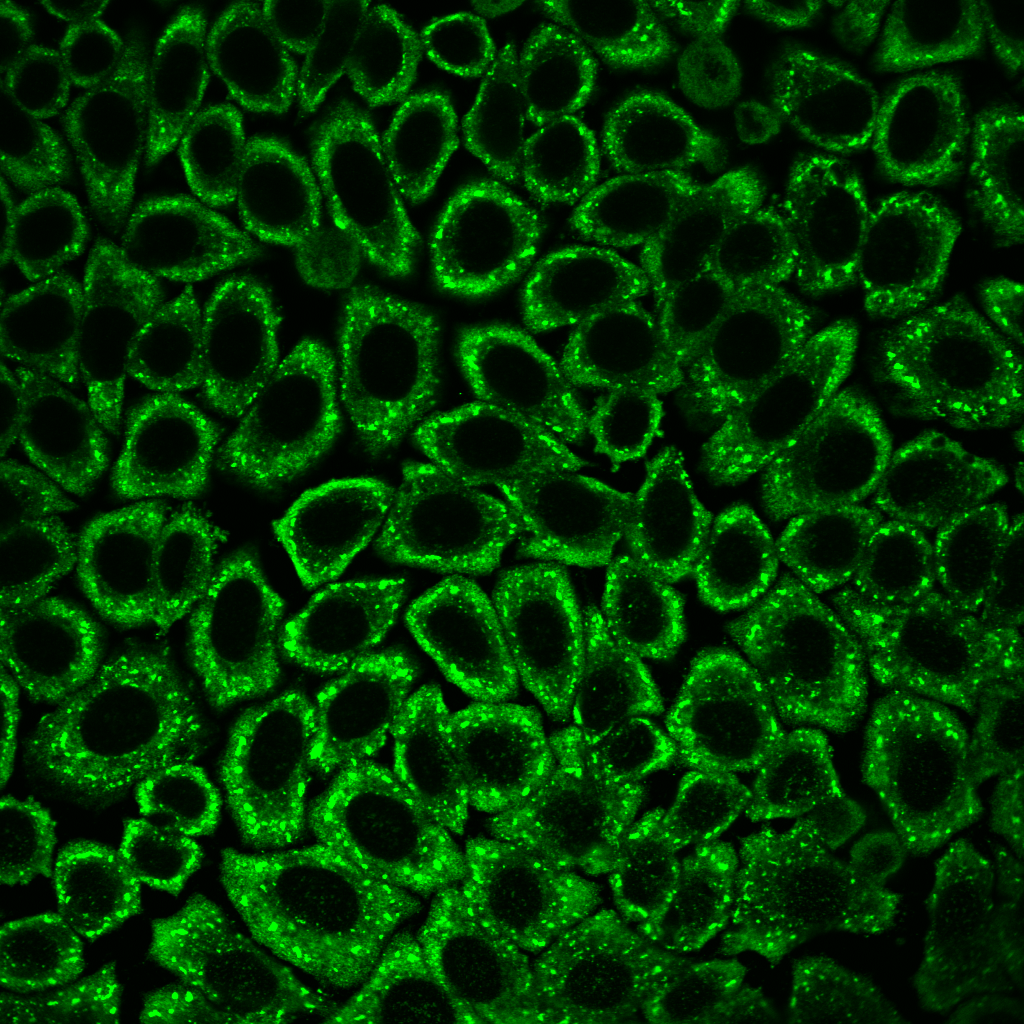

Supplement: Supplementary file 5 — Source Data Fig. 5 [file 44319_2024_98_MOESM5_ESM.zip › 5B/WT_30/wt_30 min_ctrl_60x oil 2_g3bp.tif]

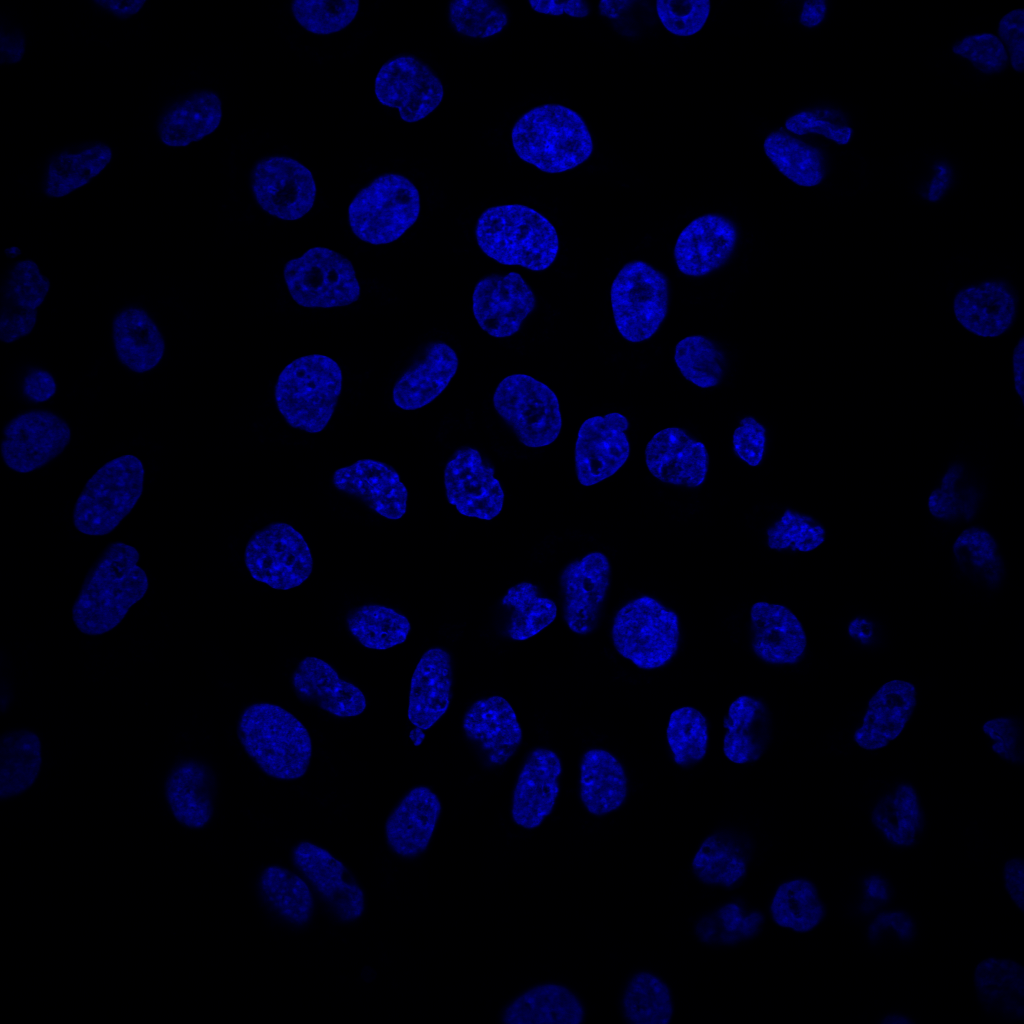

Supplement: Supplementary file 5 — Source Data Fig. 5 [file 44319_2024_98_MOESM5_ESM.zip › 5B/NAT10 KO_60/nat_60 min_ctrl_60x oil 2_dapi.tif]

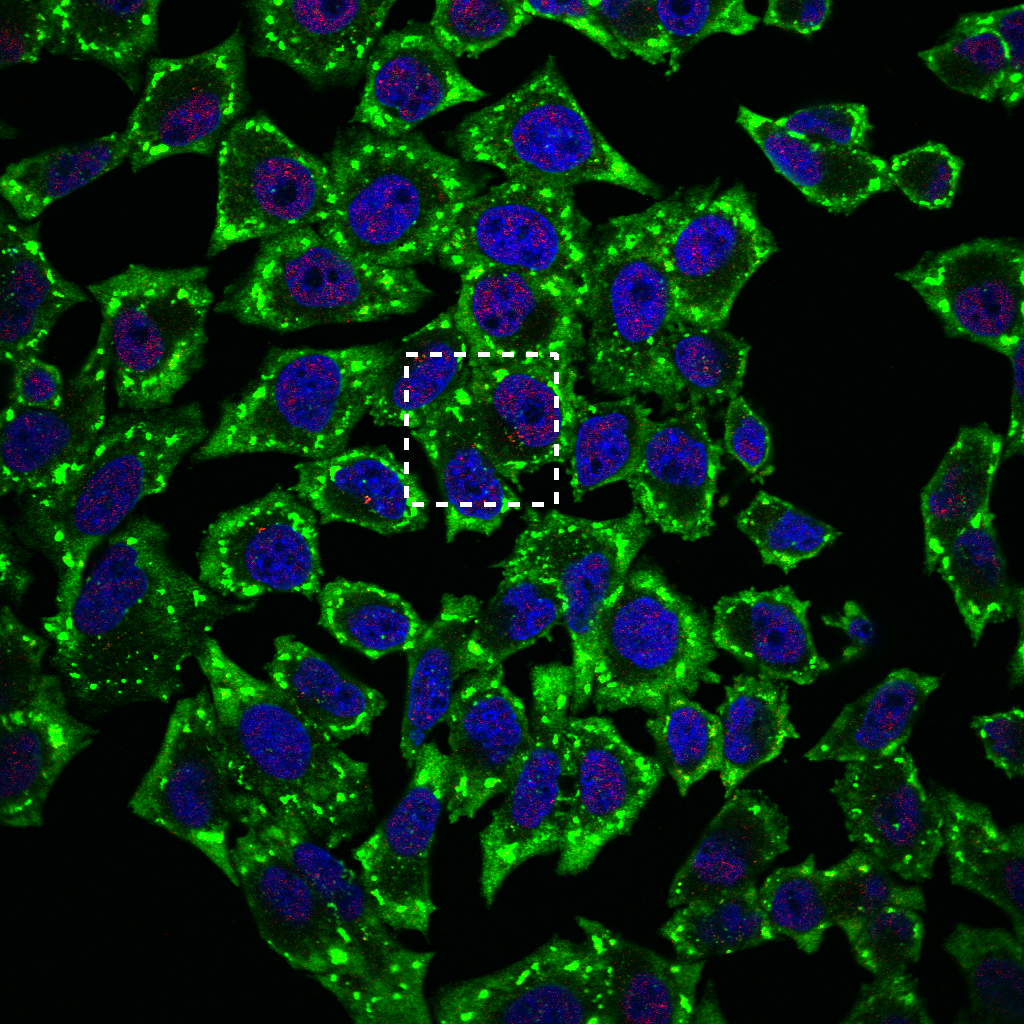

Supplement: Supplementary file 5 — Source Data Fig. 5 [file 44319_2024_98_MOESM5_ESM.zip › 5B/NAT10 KO_60/nat_60 min_ctrl_60x oil 2_merged_dashed cropped area.tif]

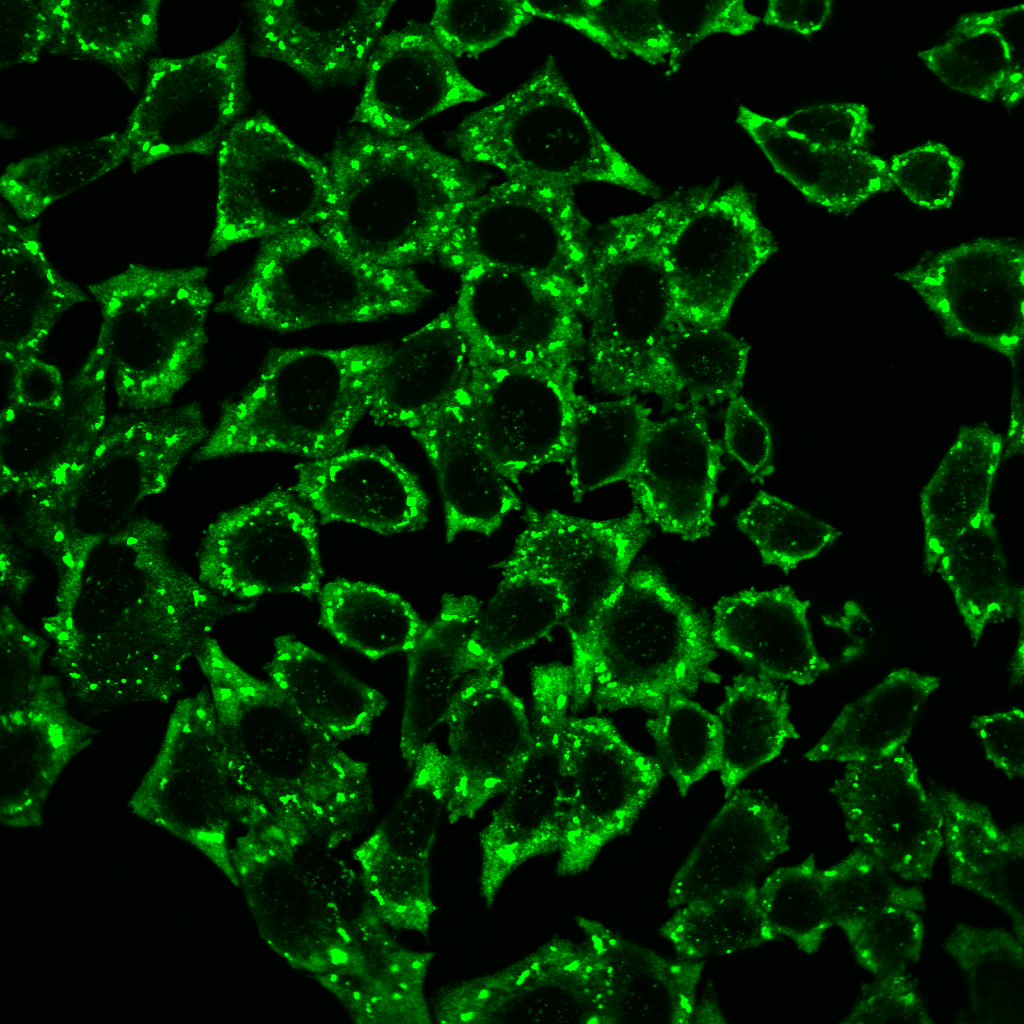

Supplement: Supplementary file 5 — Source Data Fig. 5 [file 44319_2024_98_MOESM5_ESM.zip › 5B/NAT10 KO_60/nat_60 min_ctrl_60x oil 2_g3bp.tif]

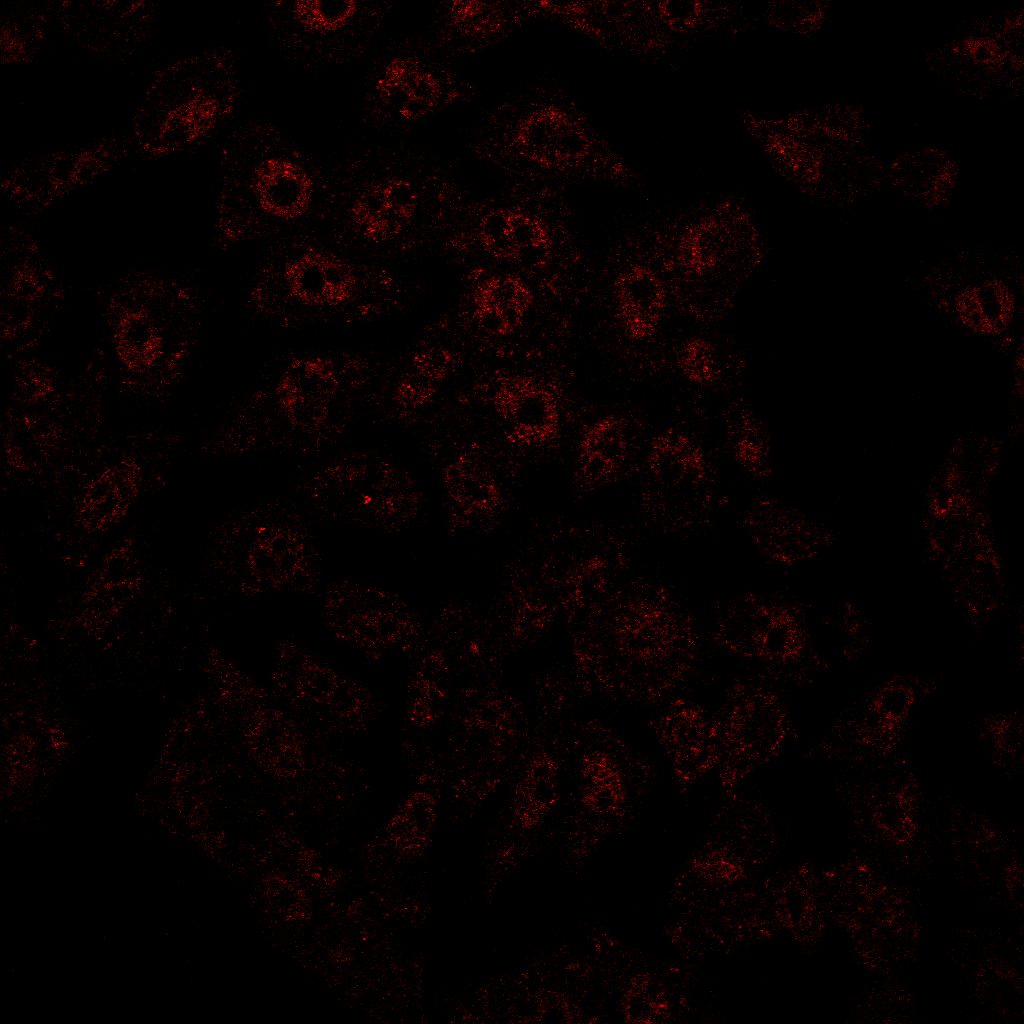

Supplement: Supplementary file 5 — Source Data Fig. 5 [file 44319_2024_98_MOESM5_ESM.zip › 5B/NAT10 KO_60/nat_60 min_ctrl_60x oil 2_ahnak.tif]

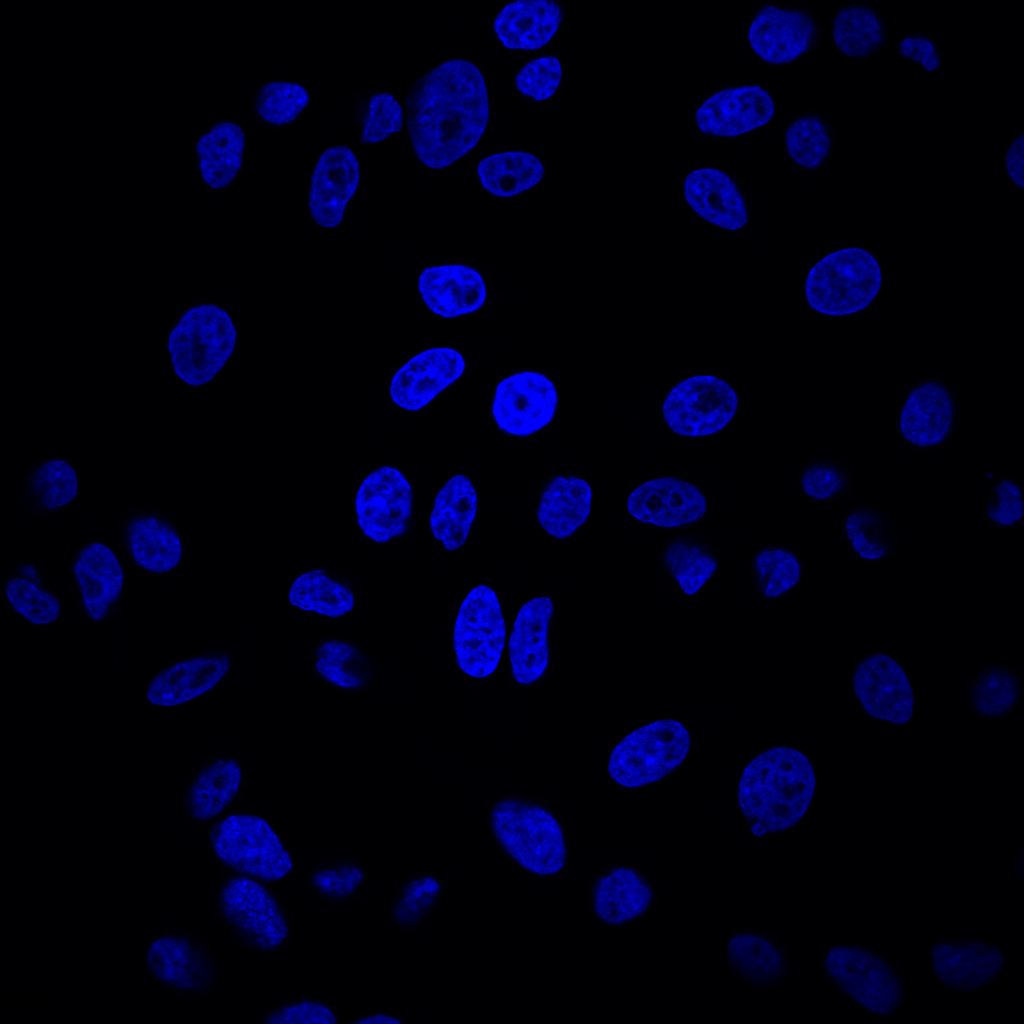

Supplement: Supplementary file 5 — Source Data Fig. 5 [file 44319_2024_98_MOESM5_ESM.zip › 5B/NAT10 KO_0/nat_ctrl_60x oil 2_dapi.tif]

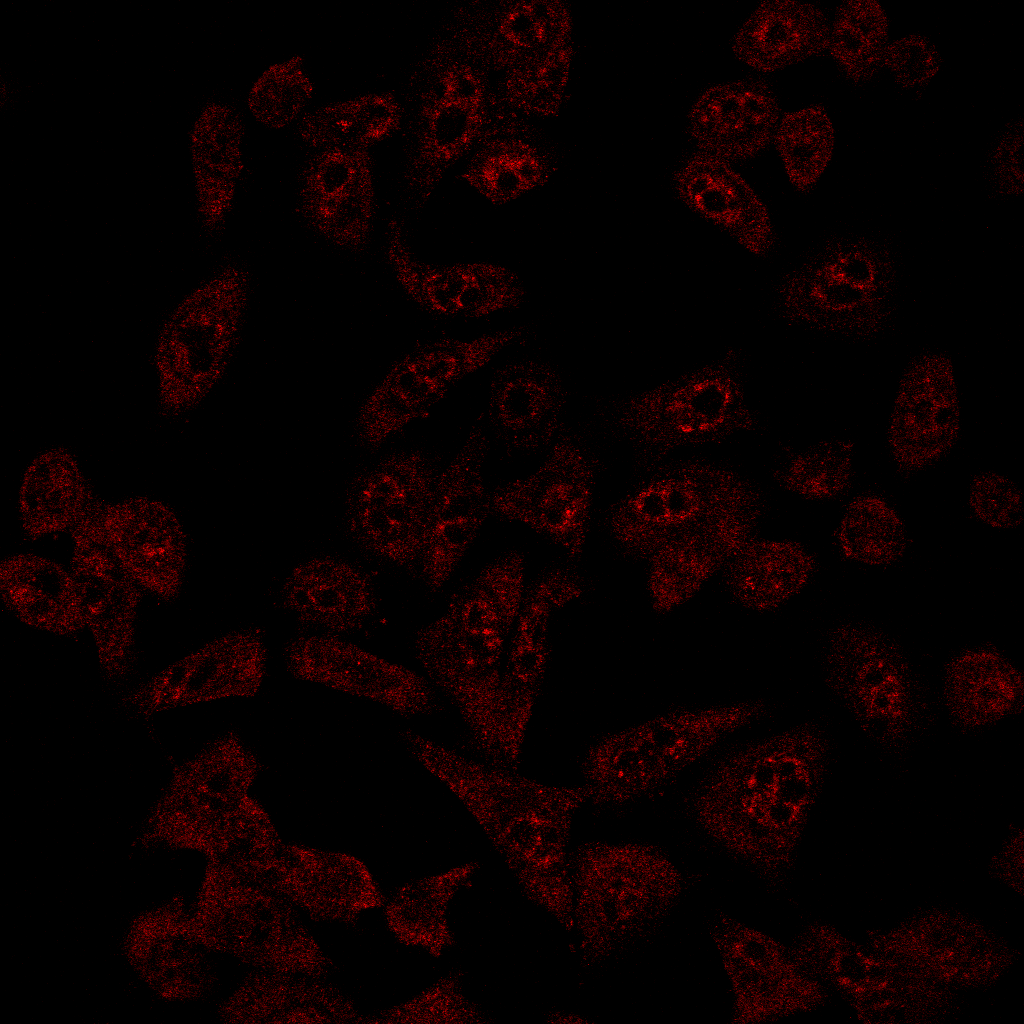

Supplement: Supplementary file 5 — Source Data Fig. 5 [file 44319_2024_98_MOESM5_ESM.zip › 5B/NAT10 KO_0/nat_ctrl_60x oil 2_ahnak.tif]

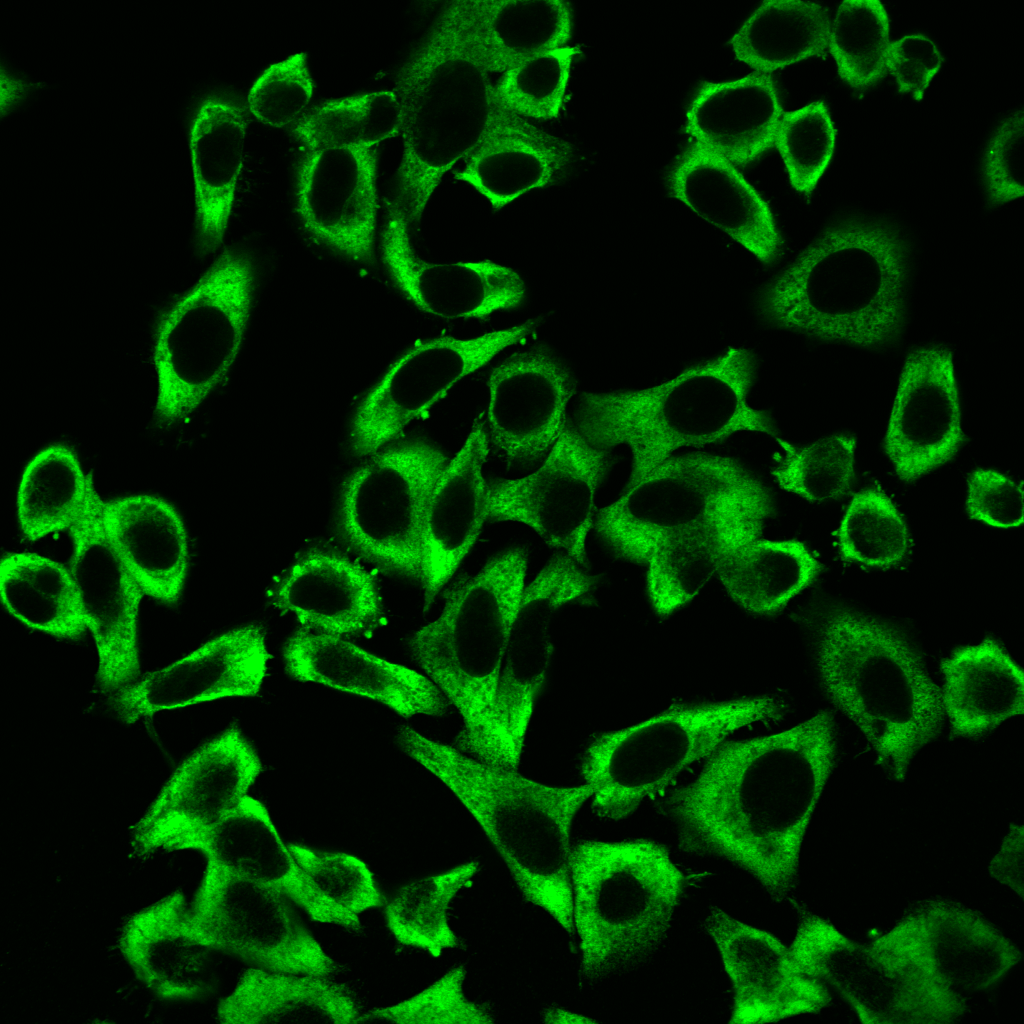

Supplement: Supplementary file 5 — Source Data Fig. 5 [file 44319_2024_98_MOESM5_ESM.zip › 5B/NAT10 KO_0/nat_ctrl_60x oil 2_g3bp.tif]

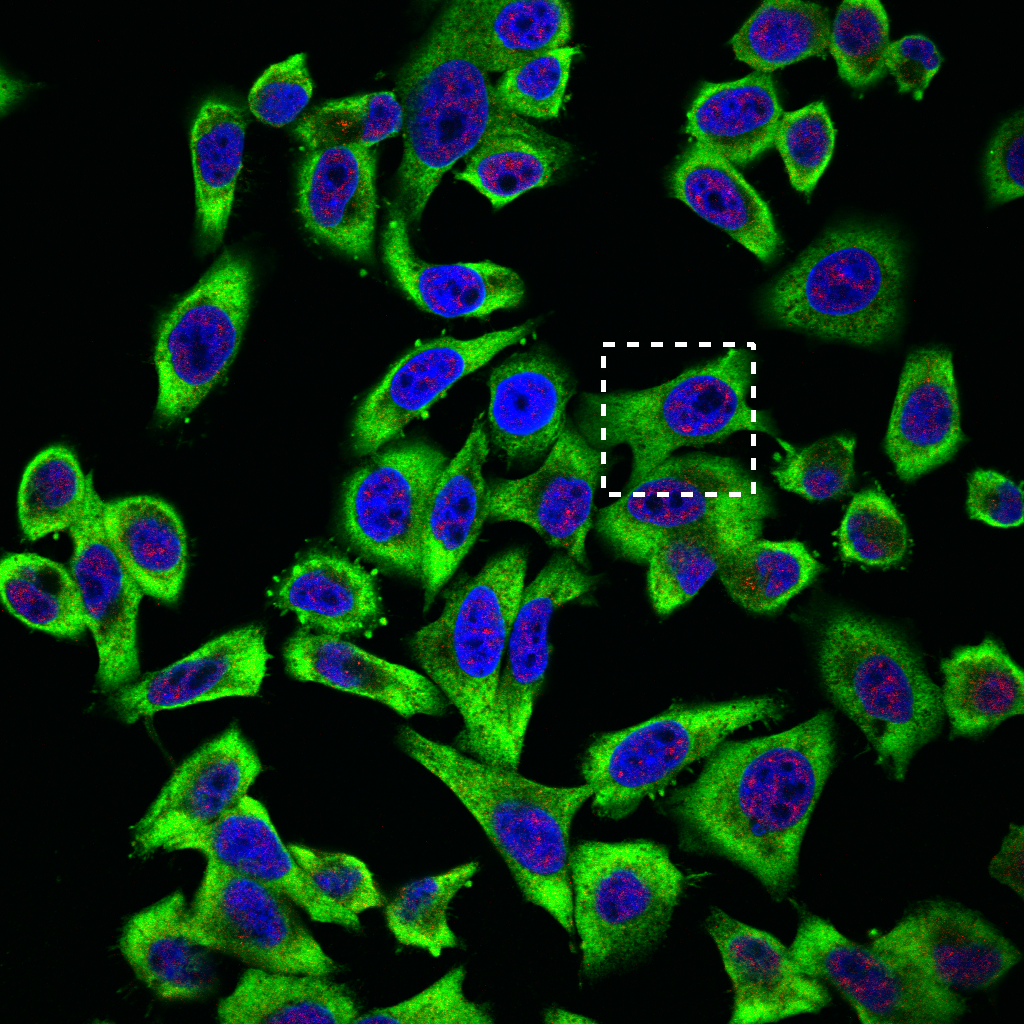

Supplement: Supplementary file 5 — Source Data Fig. 5 [file 44319_2024_98_MOESM5_ESM.zip › 5B/NAT10 KO_0/nat_ctrl_60x oil 2_merged_dashed cropped area.tif]

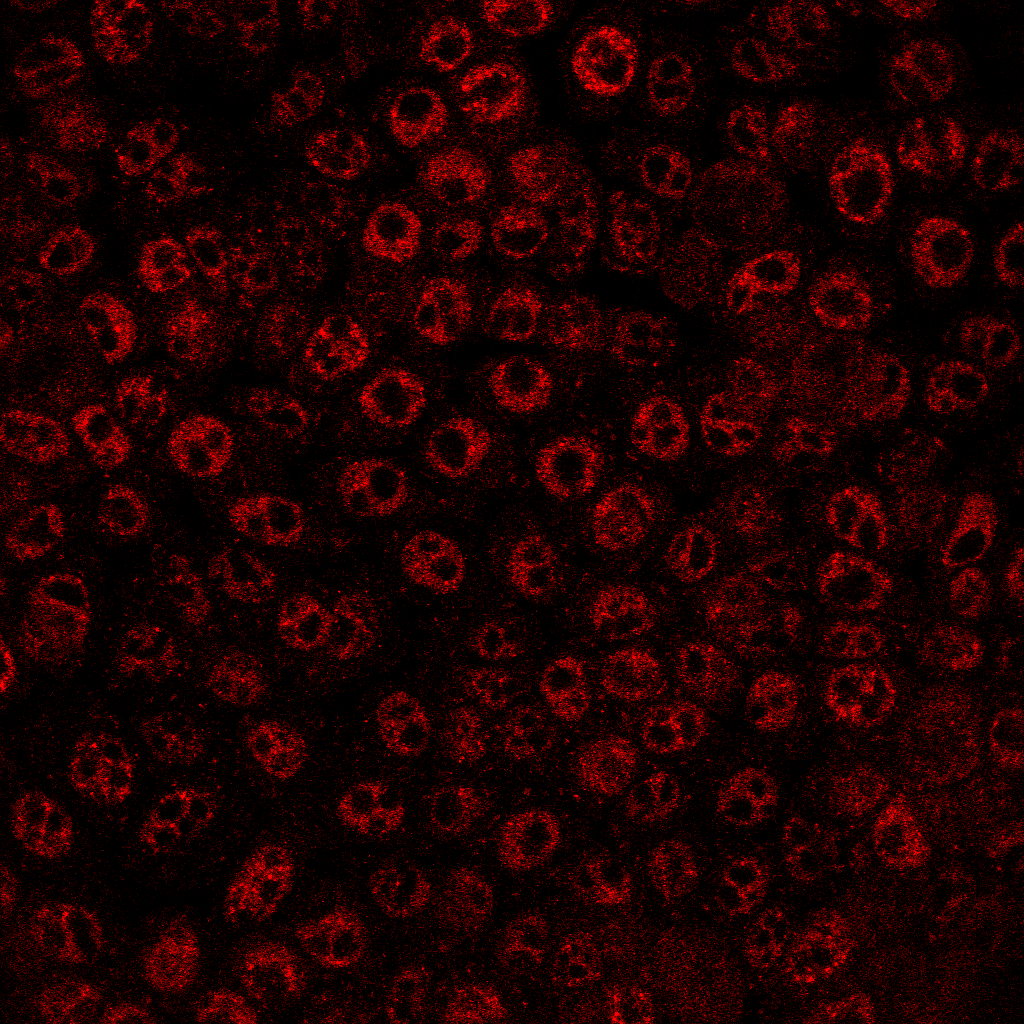

Supplement: Supplementary file 5 — Source Data Fig. 5 [file 44319_2024_98_MOESM5_ESM.zip › 5B/WT_60/wt_60 min_ctrl_60x oil 2_ahnak.tif]

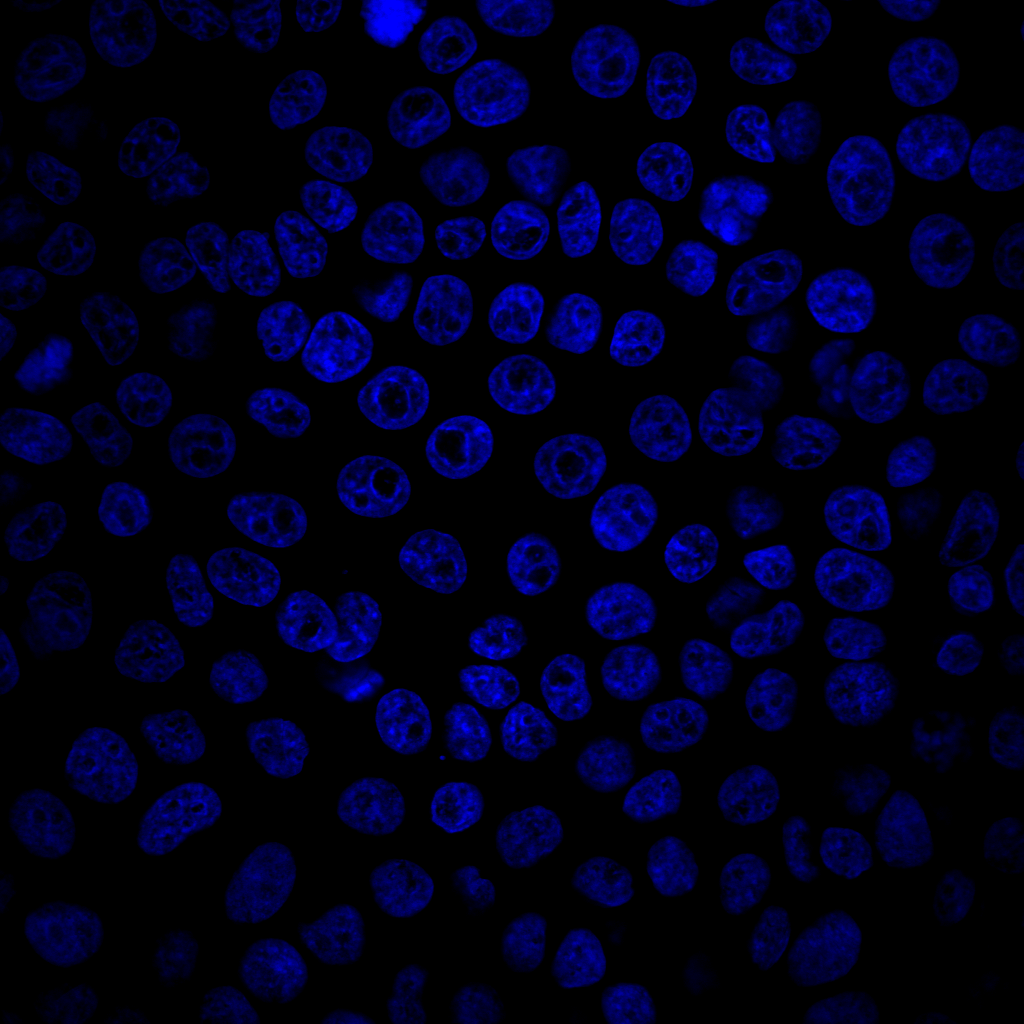

Supplement: Supplementary file 5 — Source Data Fig. 5 [file 44319_2024_98_MOESM5_ESM.zip › 5B/WT_60/wt_60 min_ctrl_60x oil 2_dapi.tif]

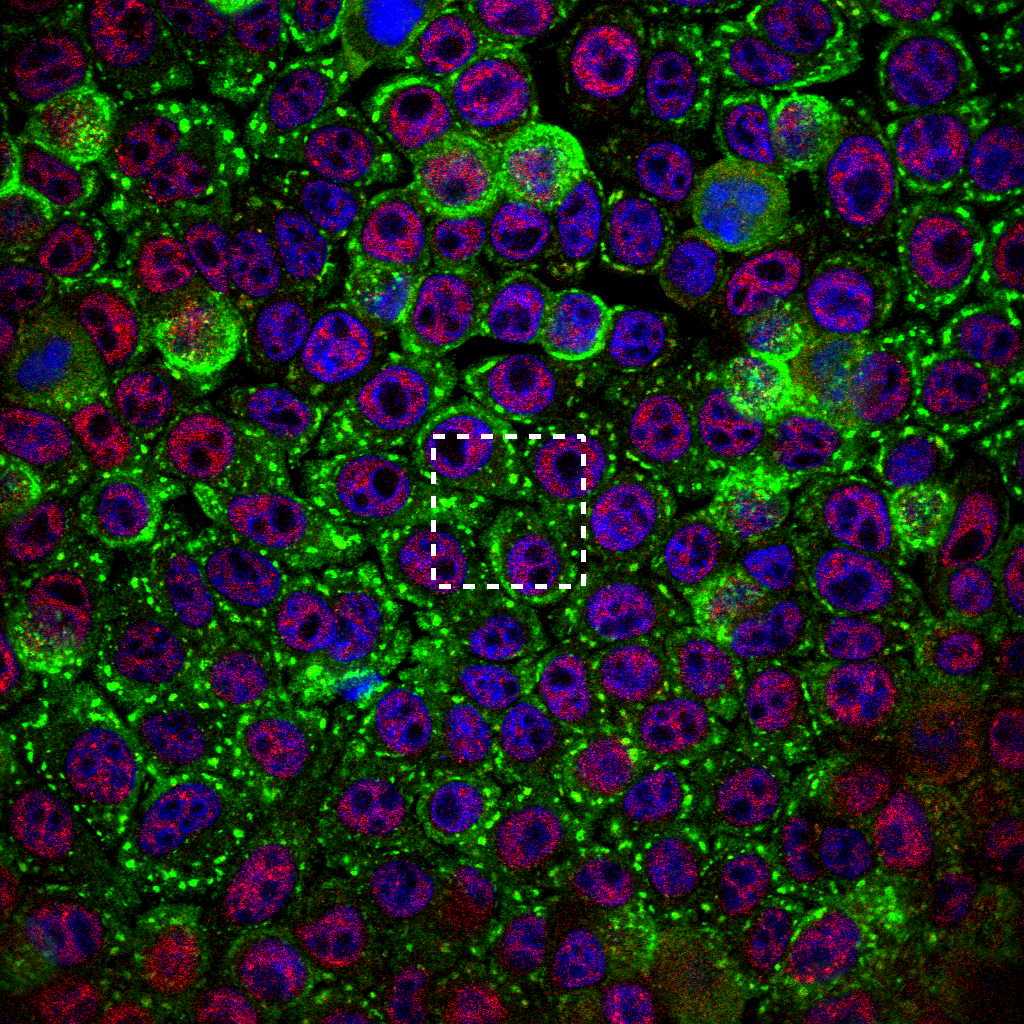

Supplement: Supplementary file 5 — Source Data Fig. 5 [file 44319_2024_98_MOESM5_ESM.zip › 5B/WT_60/wt_60 min_ctrl_60x oil 2_merged_dashed cropped area.tif]

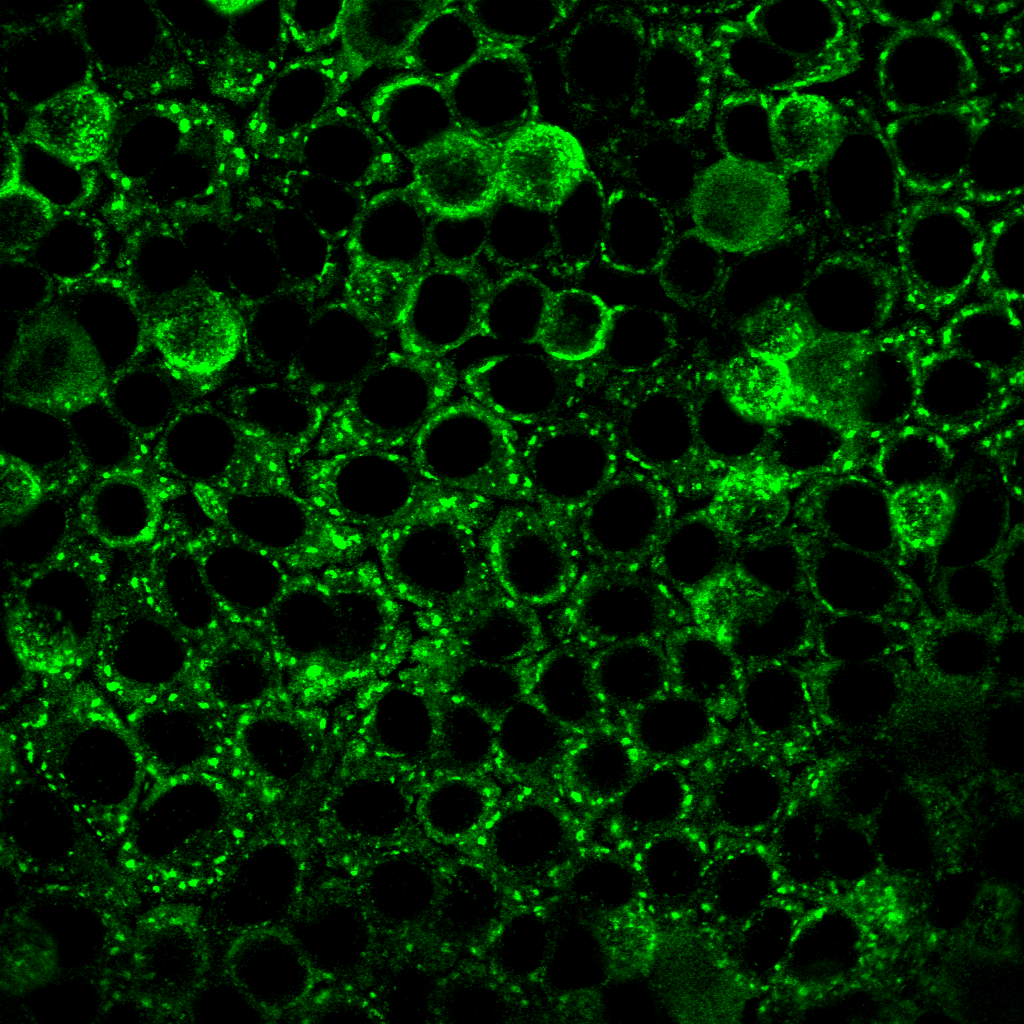

Supplement: Supplementary file 5 — Source Data Fig. 5 [file 44319_2024_98_MOESM5_ESM.zip › 5B/WT_60/wt_60 min_ctrl_60x oil 2_g3bp.tif]

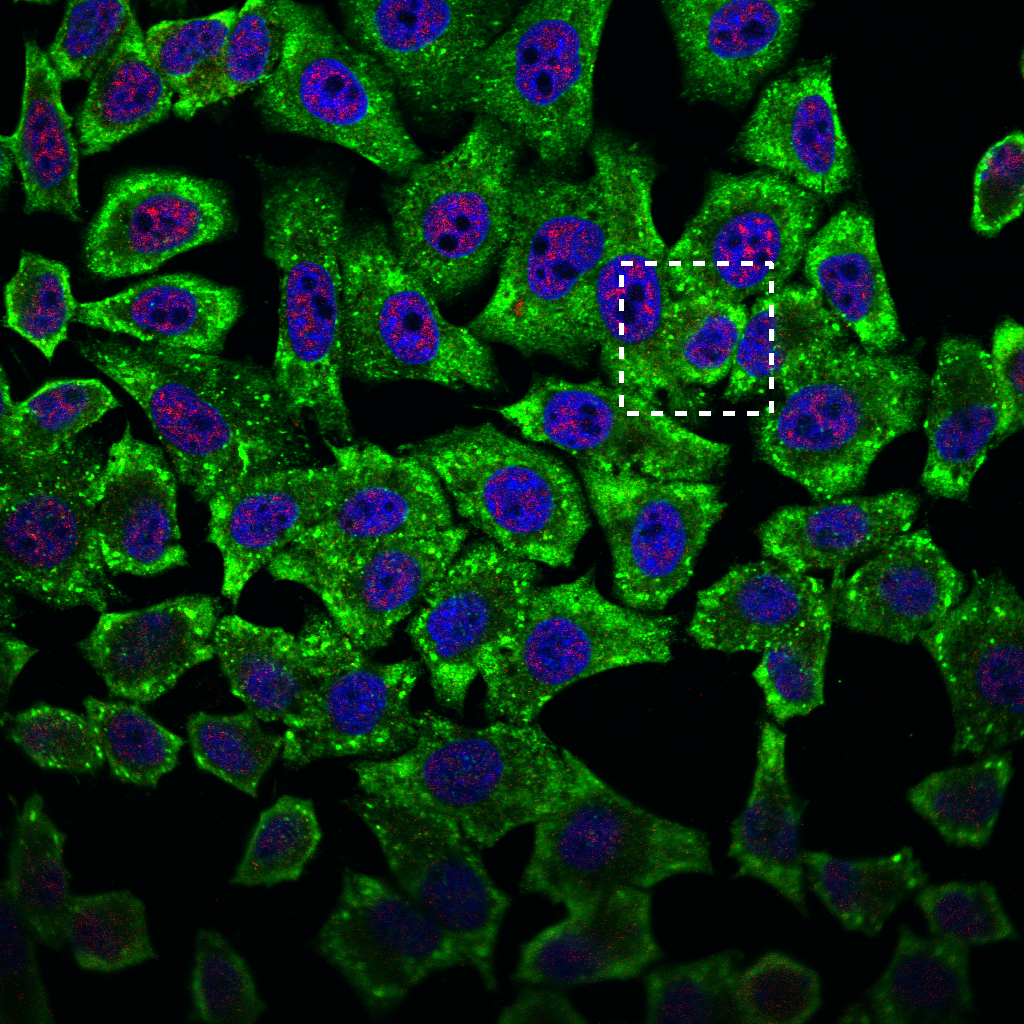

Supplement: Supplementary file 5 — Source Data Fig. 5 [file 44319_2024_98_MOESM5_ESM.zip › 5B/NAT10 KO_30/nat_30 min_ctrl_60x oil 1_merged_dashed cropped area.tif]

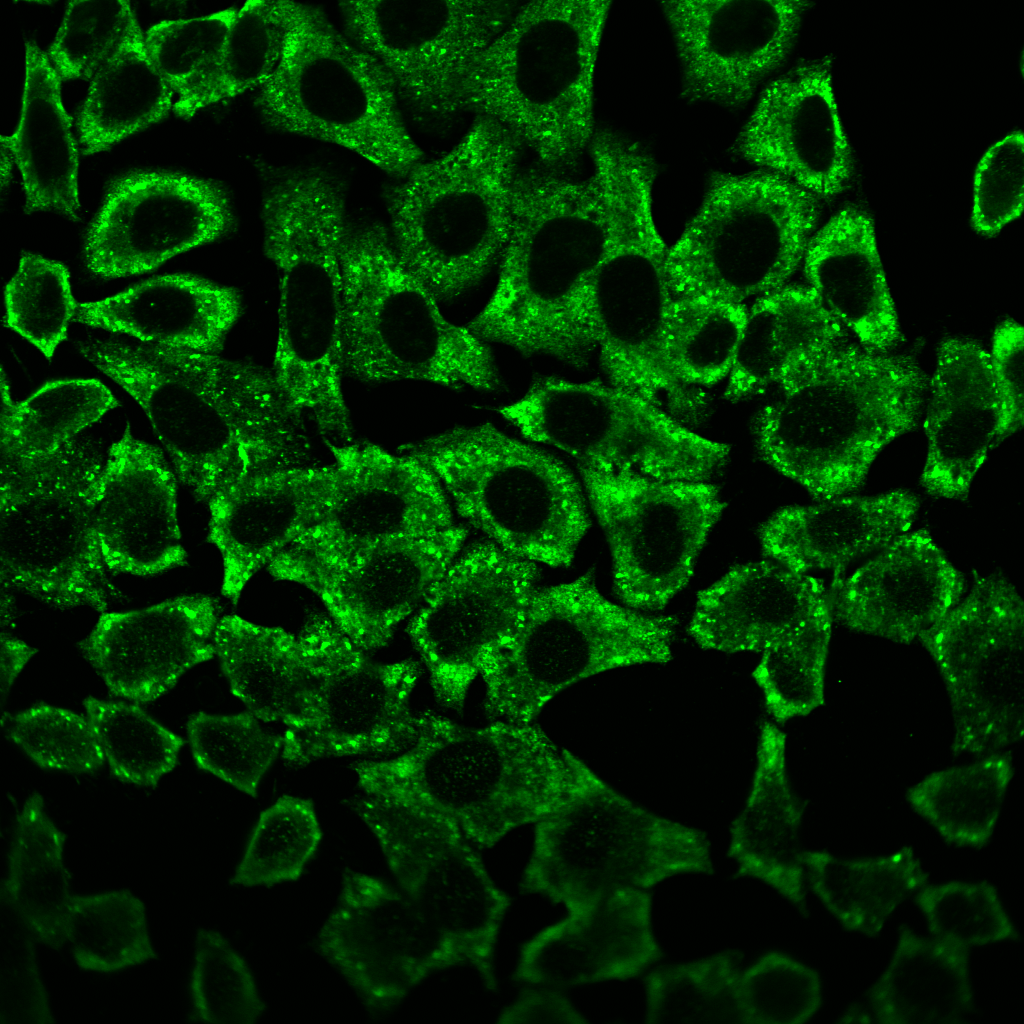

Supplement: Supplementary file 5 — Source Data Fig. 5 [file 44319_2024_98_MOESM5_ESM.zip › 5B/NAT10 KO_30/nat_30 min_ctrl_60x oil 1_g3bp.tif]

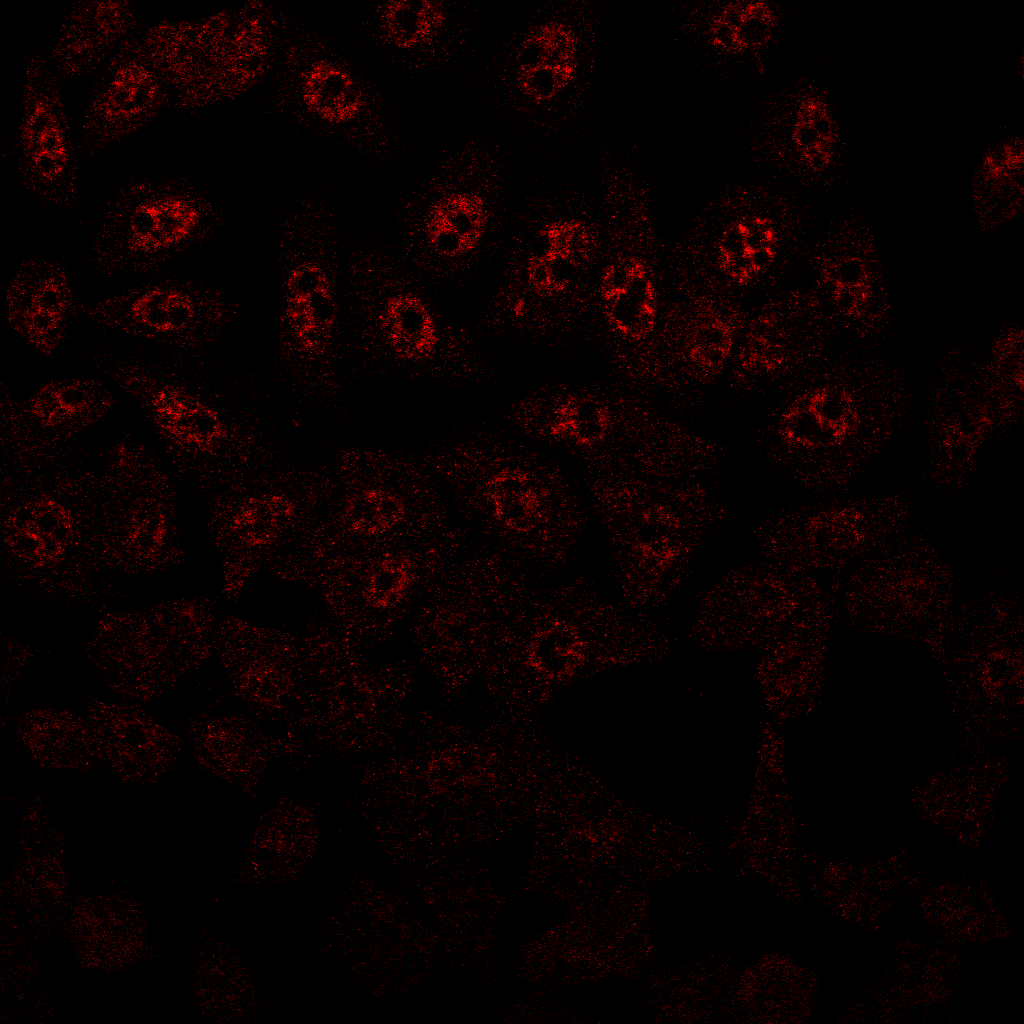

Supplement: Supplementary file 5 — Source Data Fig. 5 [file 44319_2024_98_MOESM5_ESM.zip › 5B/NAT10 KO_30/nat_30 min_ctrl_60x oil 1_ahnak.tif]

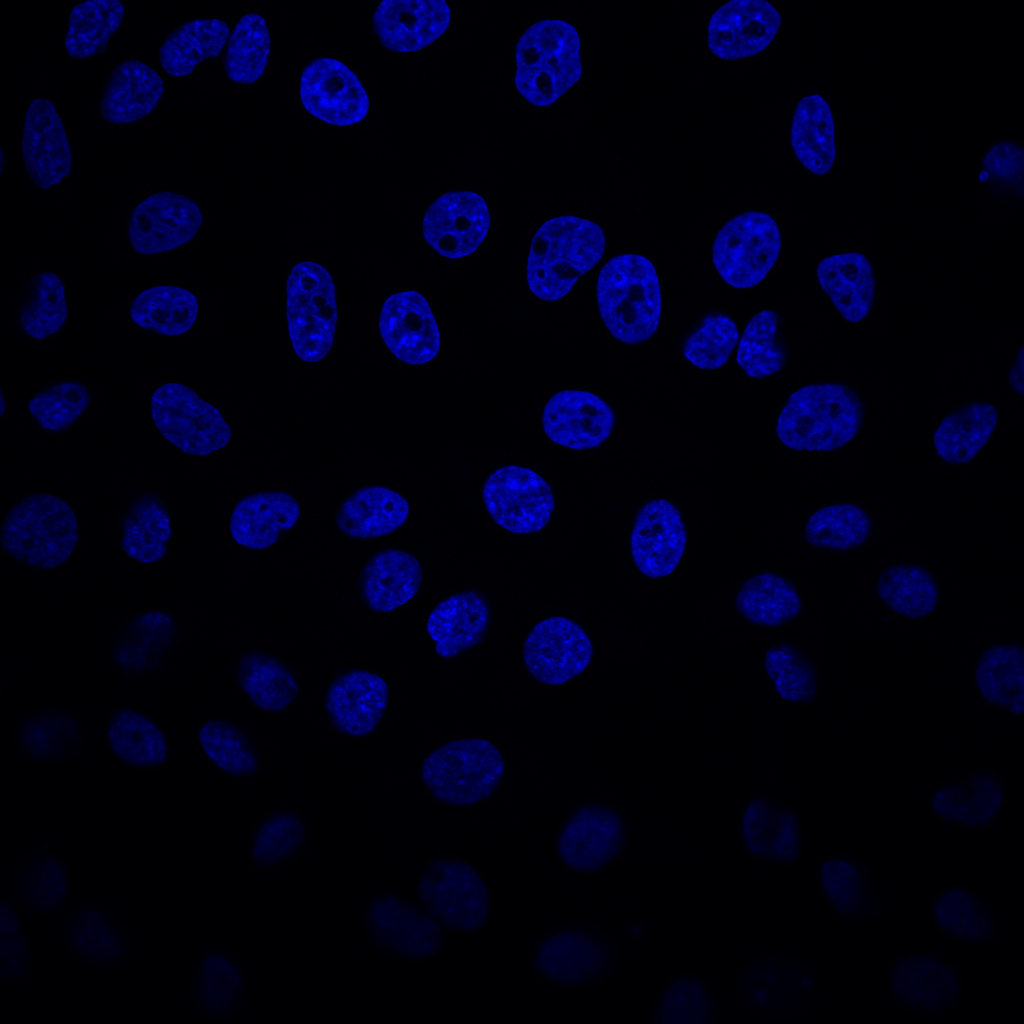

Supplement: Supplementary file 5 — Source Data Fig. 5 [file 44319_2024_98_MOESM5_ESM.zip › 5B/NAT10 KO_30/nat_30 min_ctrl_60x oil 1_dapi.tif]

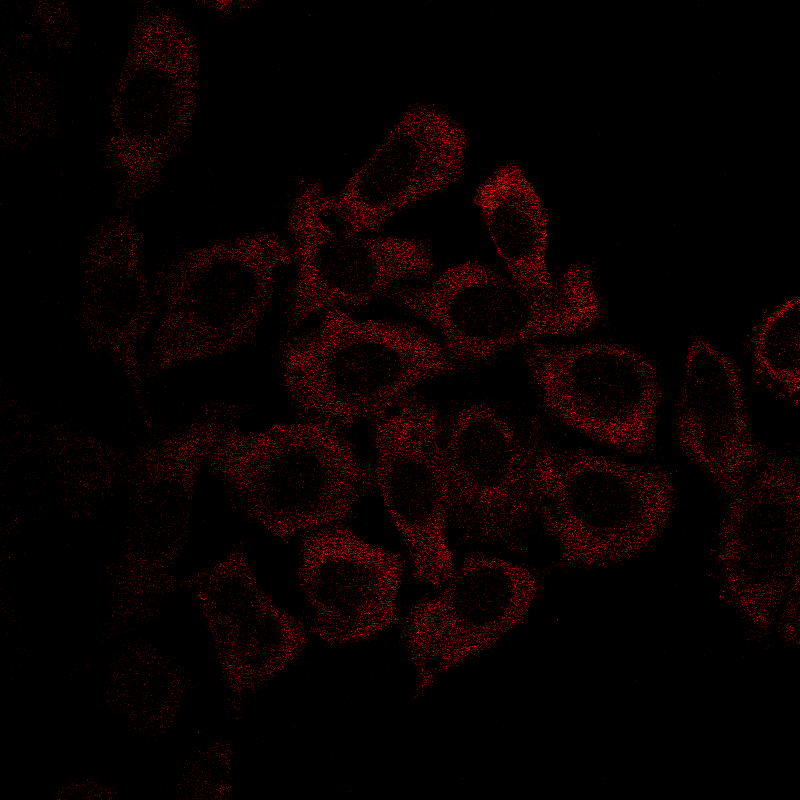

Supplement: Supplementary file 5 — Source Data Fig. 5 [file 44319_2024_98_MOESM5_ESM.zip › 5C/WT_0/wt_ctrl_60x oil 1_mki.tif]

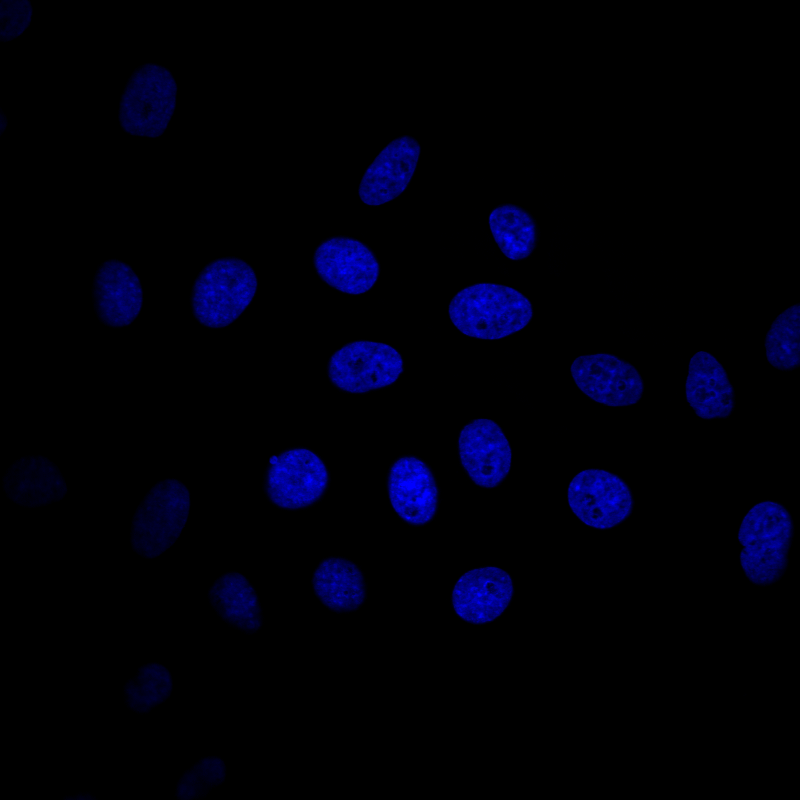

Supplement: Supplementary file 5 — Source Data Fig. 5 [file 44319_2024_98_MOESM5_ESM.zip › 5C/WT_0/wt_ctrl_60x oil 1_dapi.tif]

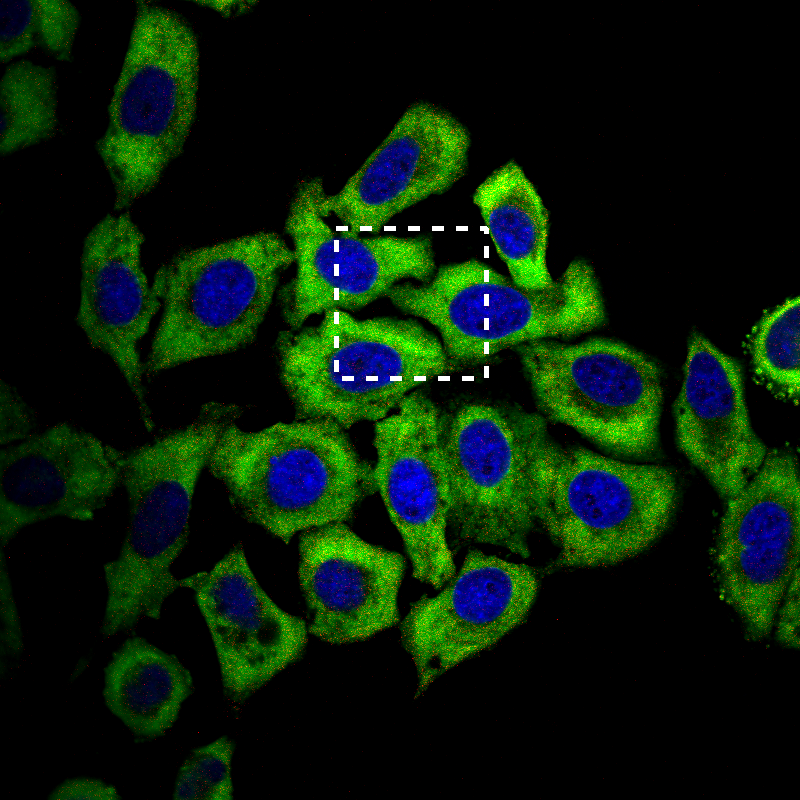

Supplement: Supplementary file 5 — Source Data Fig. 5 [file 44319_2024_98_MOESM5_ESM.zip › 5C/WT_0/wt_ctrl_60x oil 1_merged_dashed cropped area.tif]

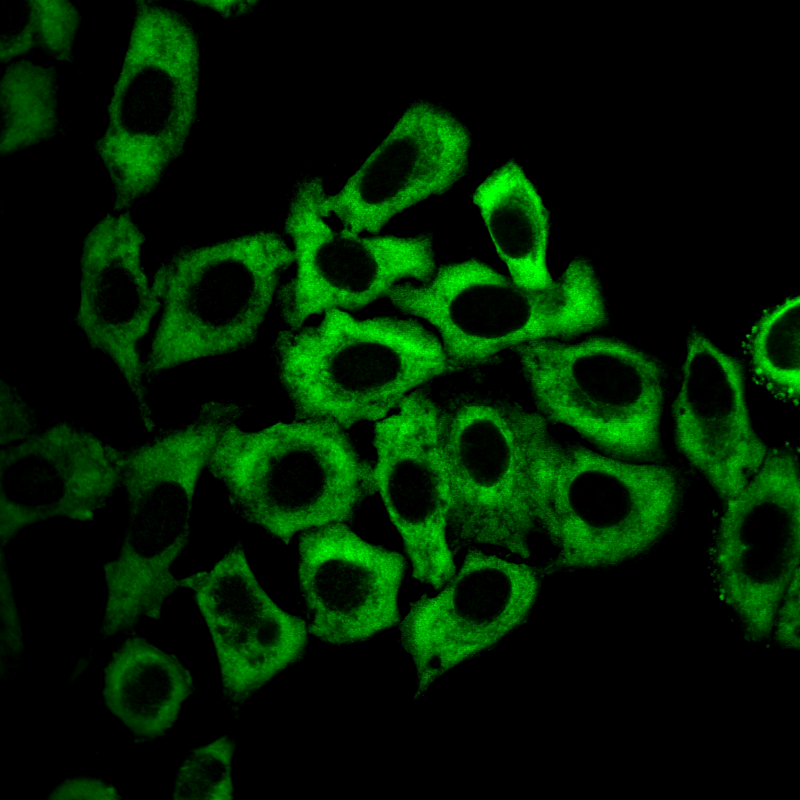

Supplement: Supplementary file 5 — Source Data Fig. 5 [file 44319_2024_98_MOESM5_ESM.zip › 5C/WT_0/wt_ctrl_60x oil 1_g3bp.tif]

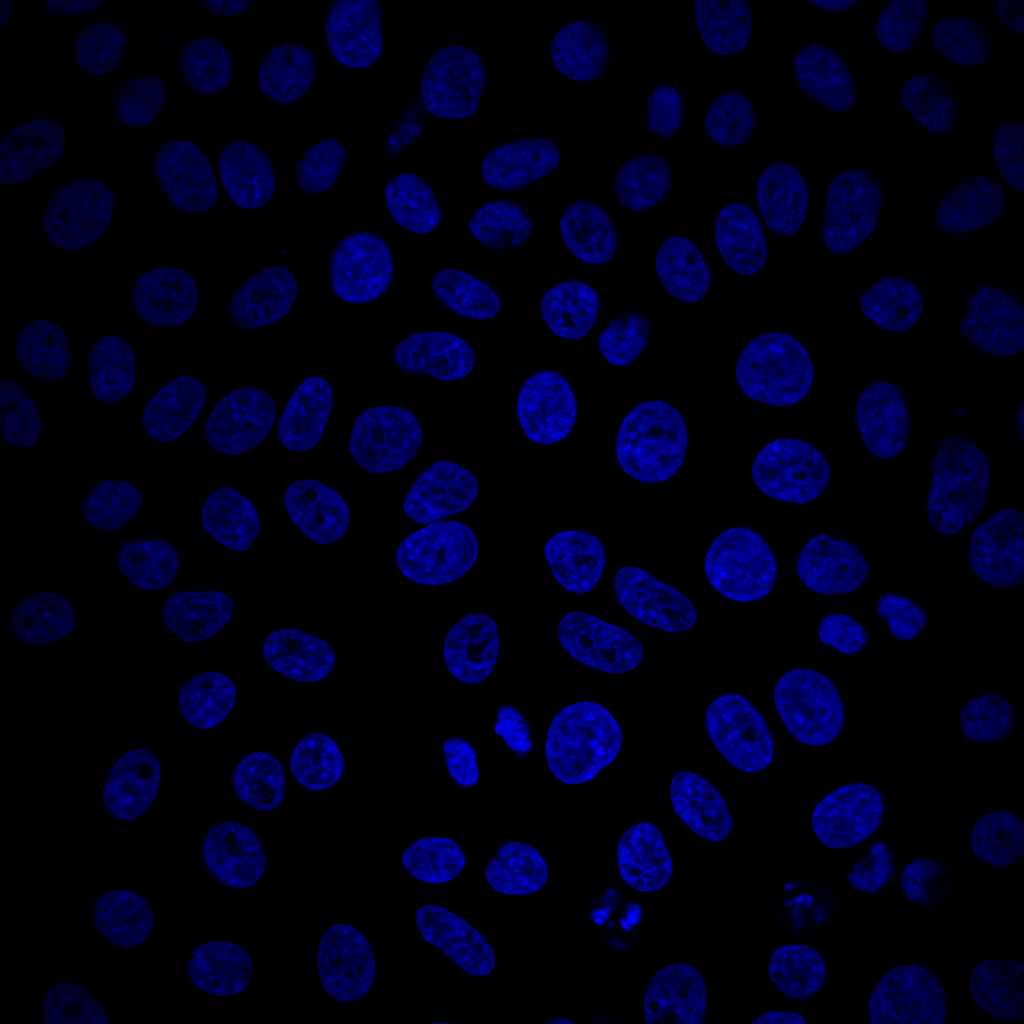

Supplement: Supplementary file 5 — Source Data Fig. 5 [file 44319_2024_98_MOESM5_ESM.zip › 5C/WT_30/wt_30 min_ctrl_60x oil 2_dapi.tif]

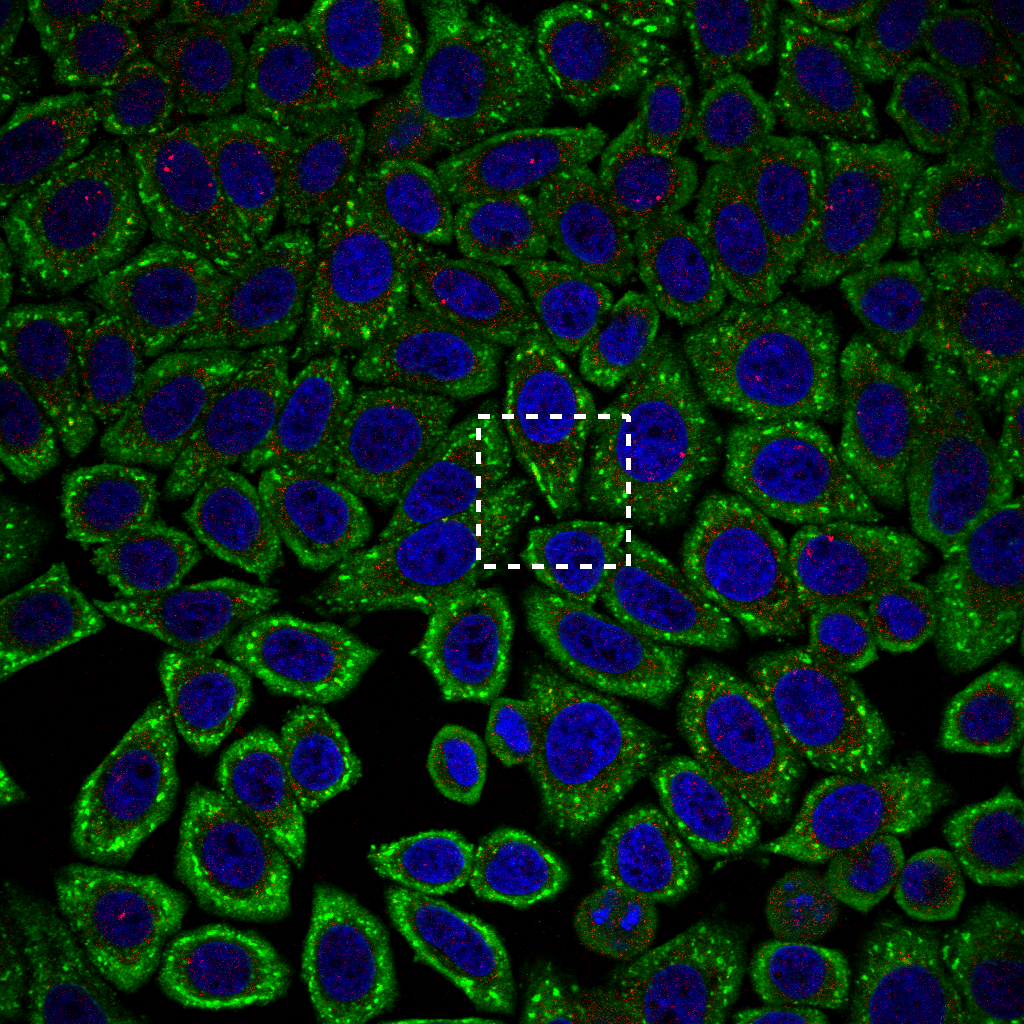

Supplement: Supplementary file 5 — Source Data Fig. 5 [file 44319_2024_98_MOESM5_ESM.zip › 5C/WT_30/wt_30 min_ctrl_60x oil 2_merged_dashed cropped area.tif]

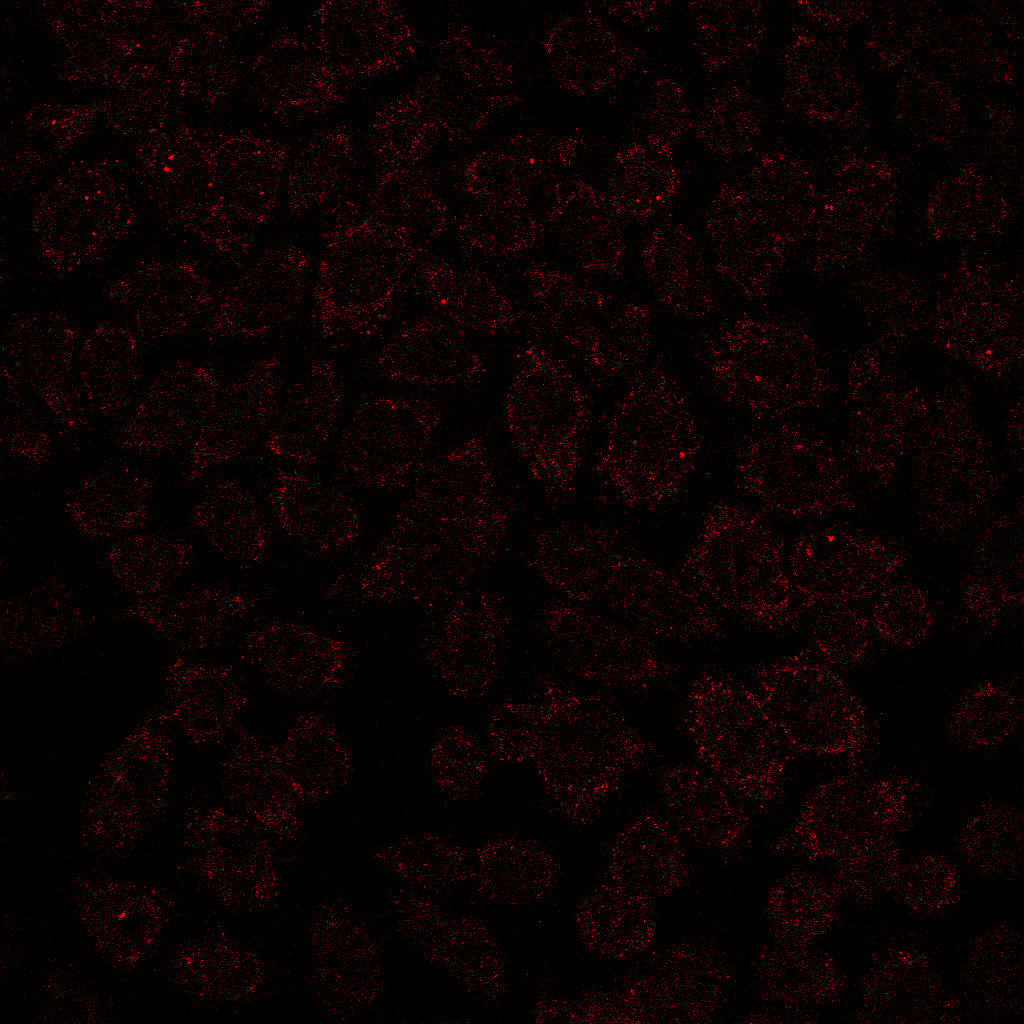

Supplement: Supplementary file 5 — Source Data Fig. 5 [file 44319_2024_98_MOESM5_ESM.zip › 5C/WT_30/wt_30 min_ctrl_60x oil 2_mki.tif]

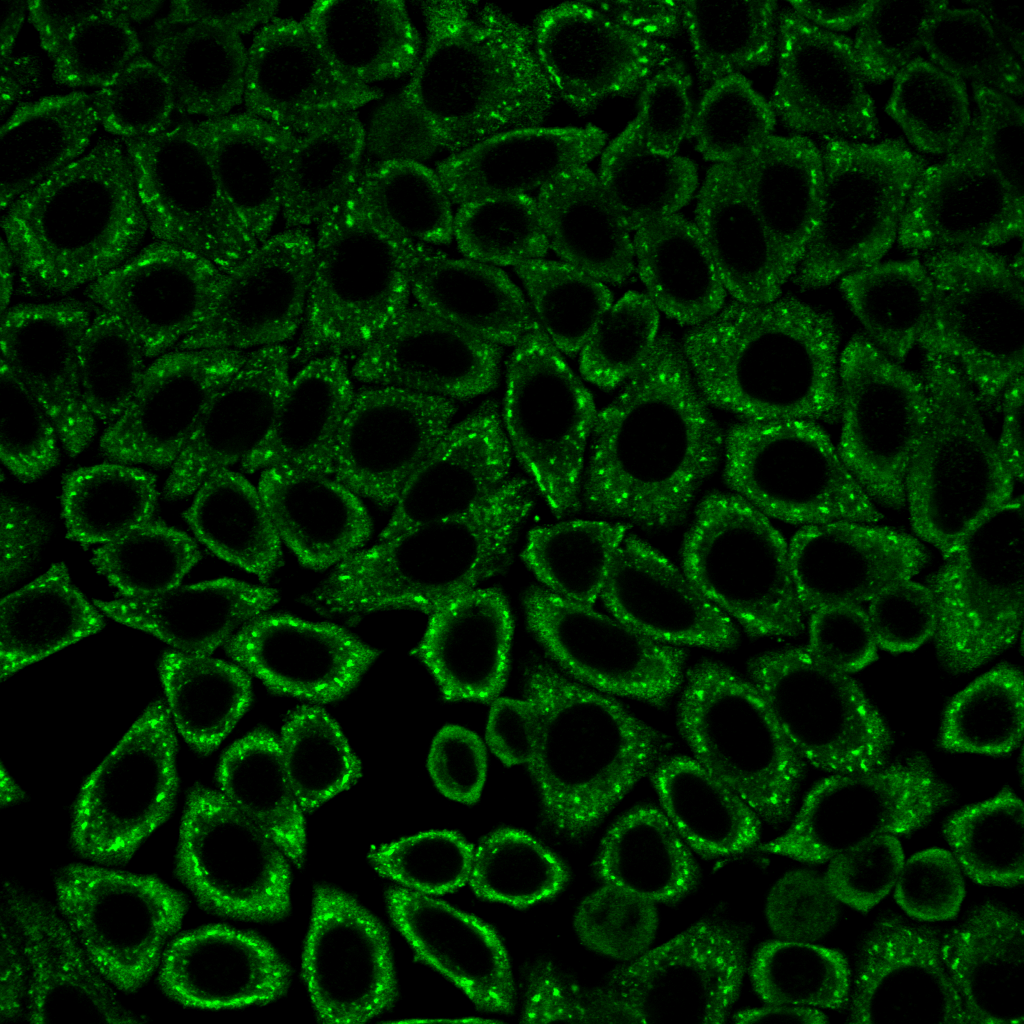

Supplement: Supplementary file 5 — Source Data Fig. 5 [file 44319_2024_98_MOESM5_ESM.zip › 5C/WT_30/wt_30 min_ctrl_60x oil 2_g3bp.tif]

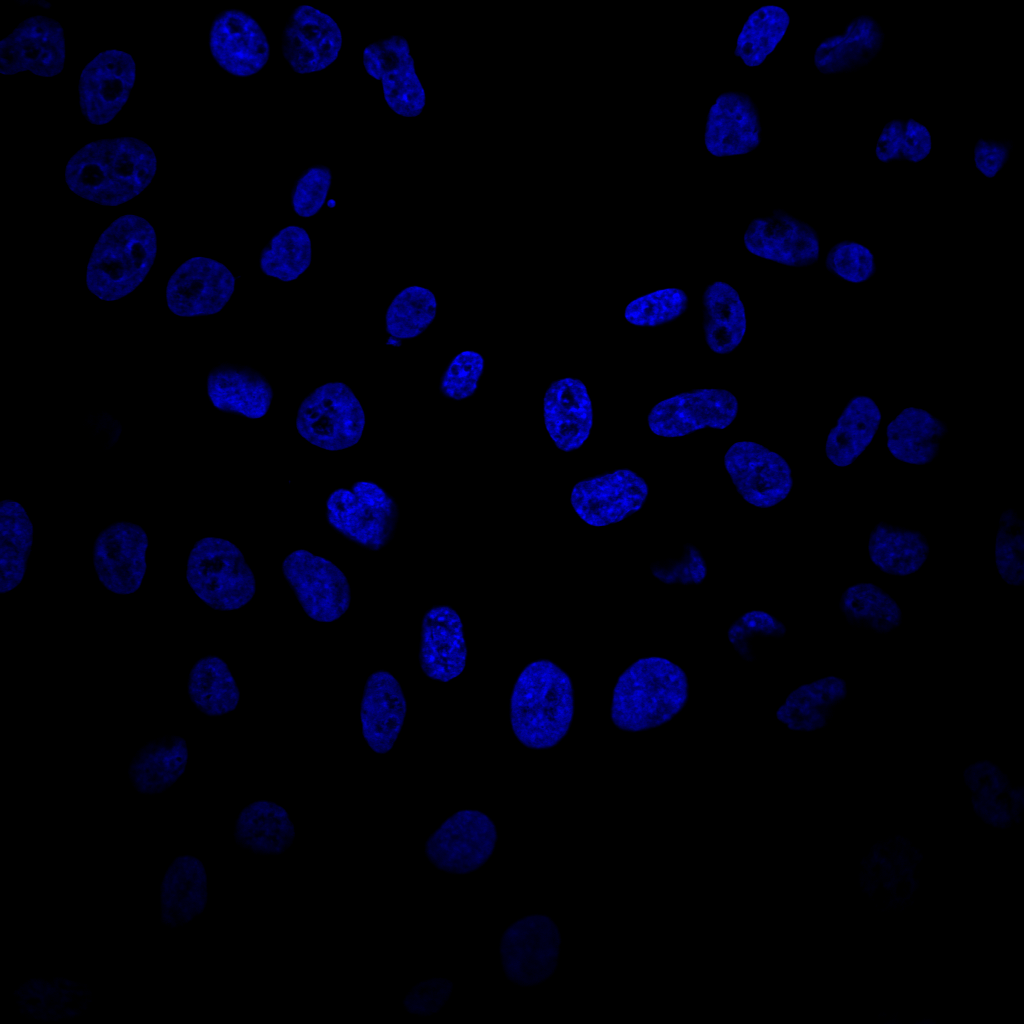

Supplement: Supplementary file 5 — Source Data Fig. 5 [file 44319_2024_98_MOESM5_ESM.zip › 5C/NAT10 KO_60/nat_60 min_ctrl_60x oil 2_dapi.tif]

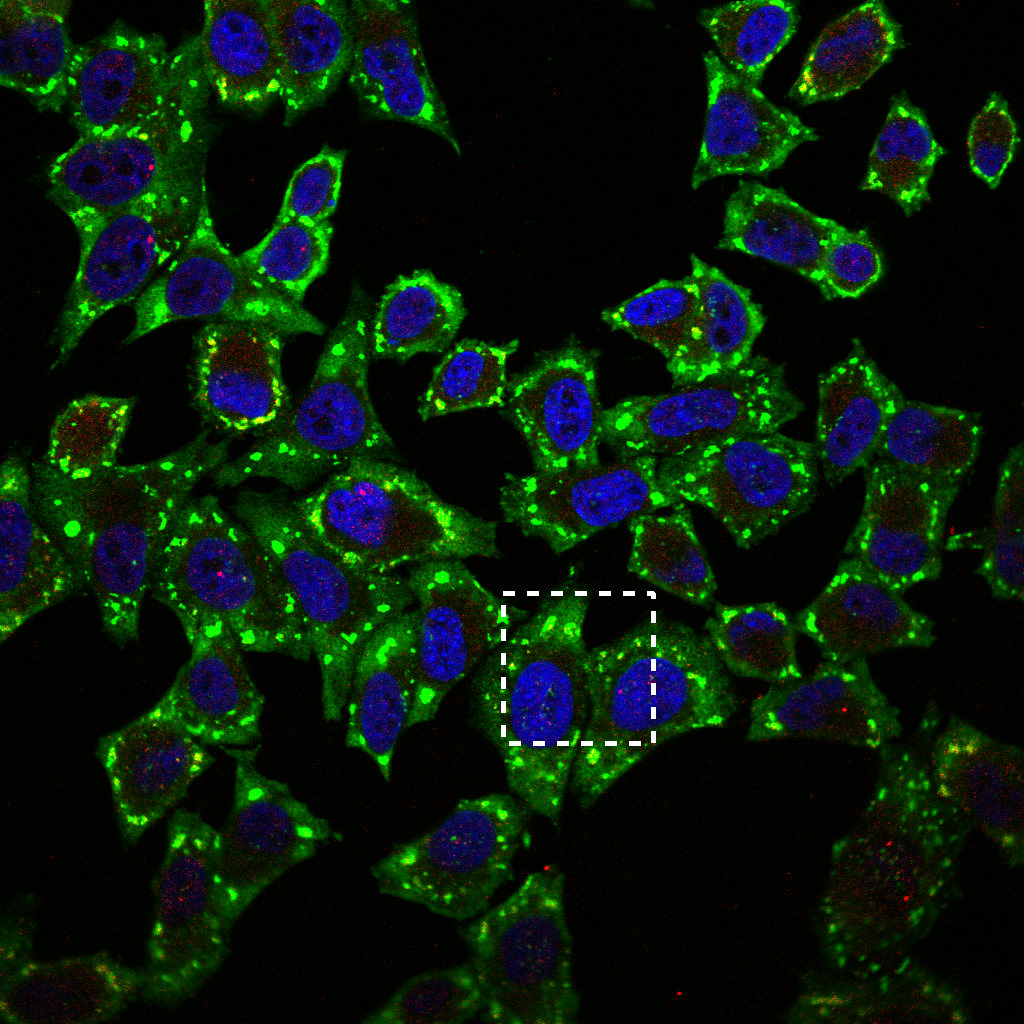

Supplement: Supplementary file 5 — Source Data Fig. 5 [file 44319_2024_98_MOESM5_ESM.zip › 5C/NAT10 KO_60/nat_60 min_ctrl_60x oil 2_merged_dashed cropped area.tif]

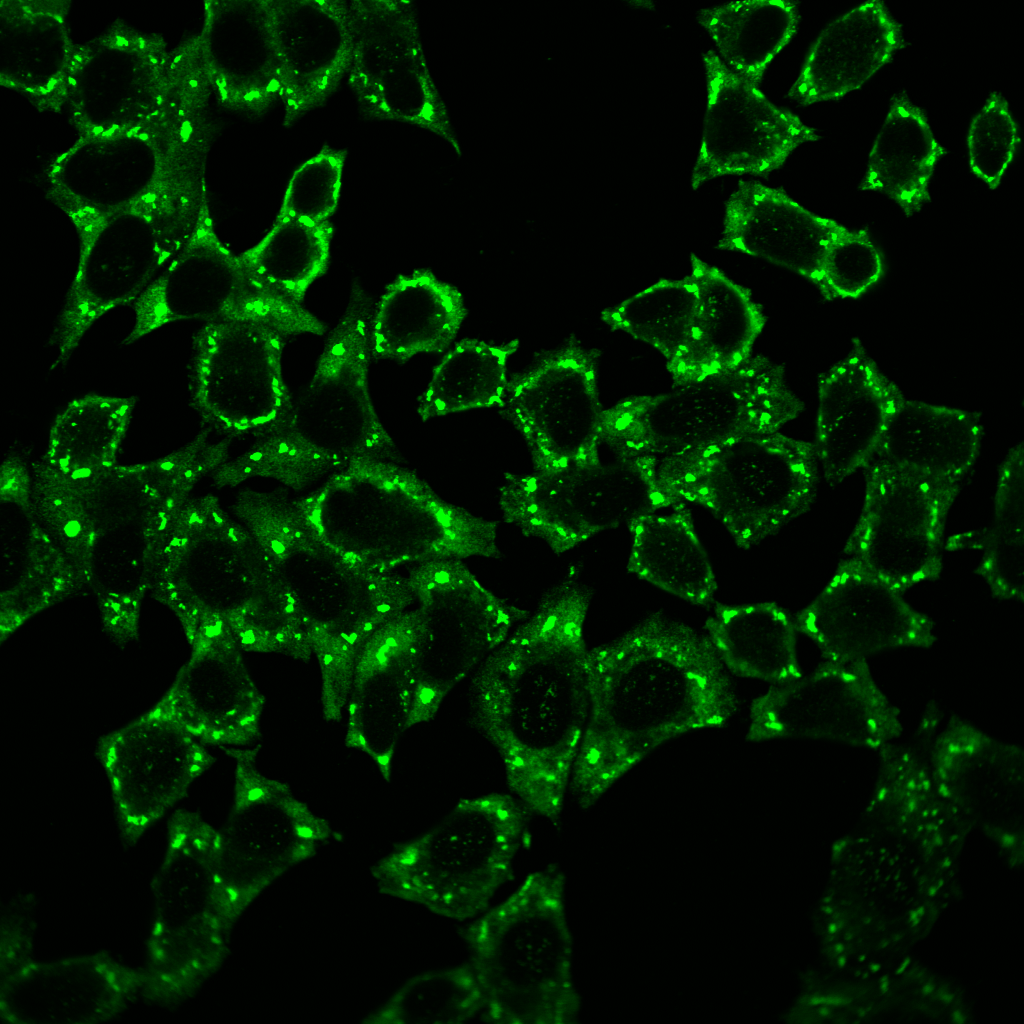

Supplement: Supplementary file 5 — Source Data Fig. 5 [file 44319_2024_98_MOESM5_ESM.zip › 5C/NAT10 KO_60/nat_60 min_ctrl_60x oil 2_g3bp.tif]

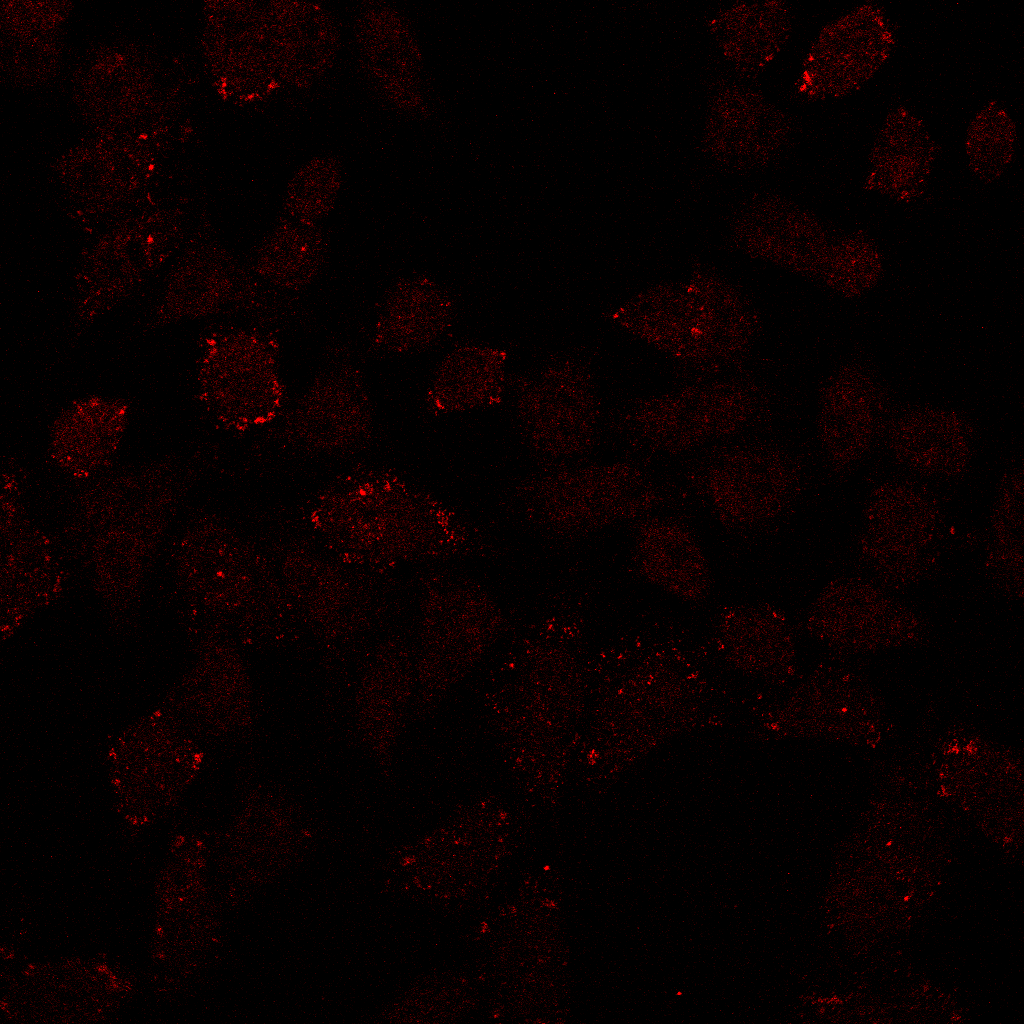

Supplement: Supplementary file 5 — Source Data Fig. 5 [file 44319_2024_98_MOESM5_ESM.zip › 5C/NAT10 KO_60/nat_60 min_ctrl_60x oil 2_mki.tif]

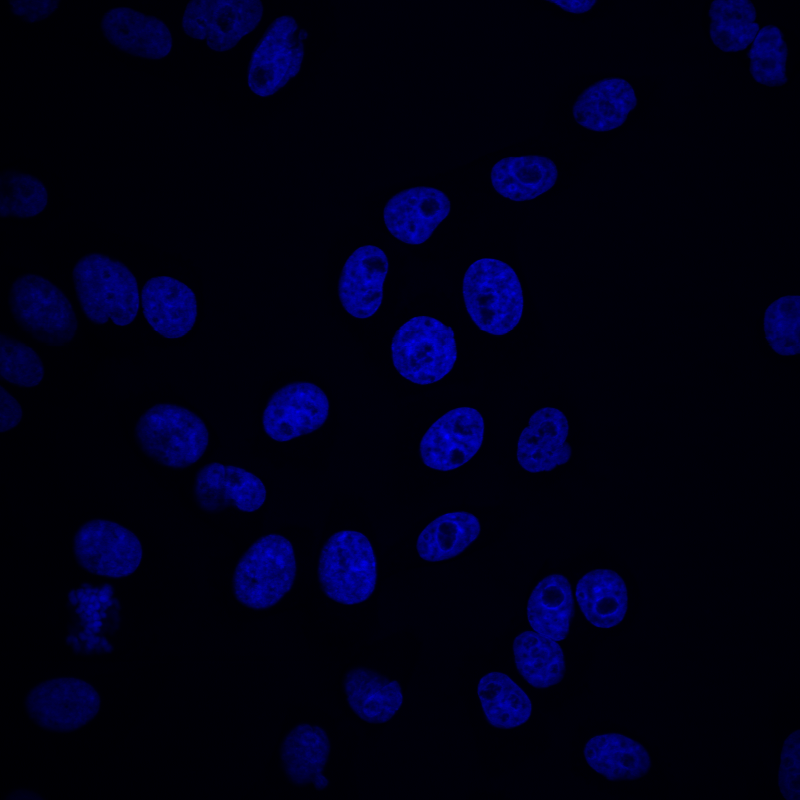

Supplement: Supplementary file 5 — Source Data Fig. 5 [file 44319_2024_98_MOESM5_ESM.zip › 5C/NAT10 KO_0/nat_ctrl_60x oil 2_dapi.tif]

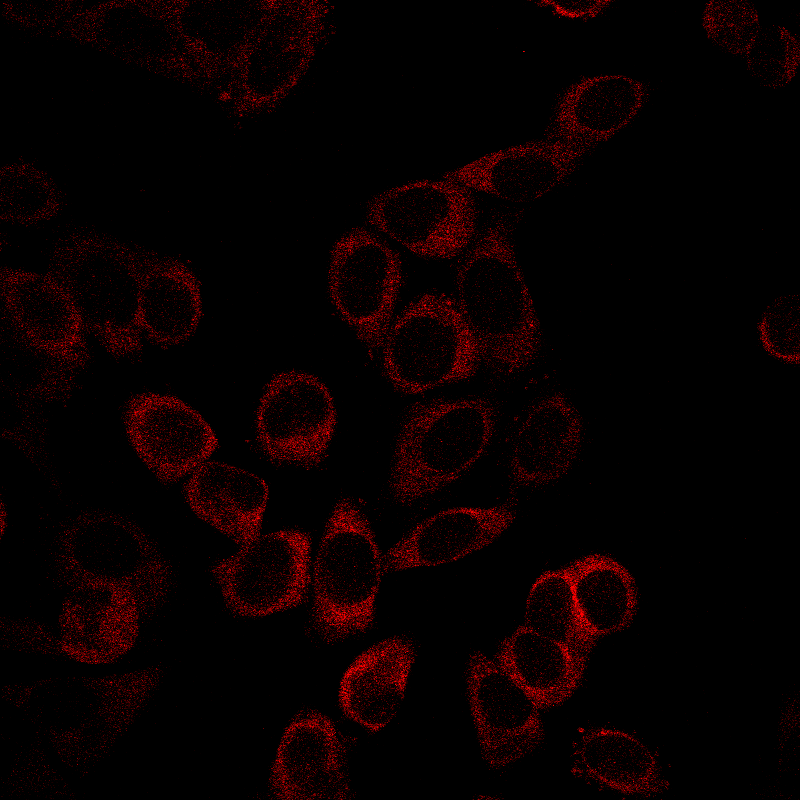

Supplement: Supplementary file 5 — Source Data Fig. 5 [file 44319_2024_98_MOESM5_ESM.zip › 5C/NAT10 KO_0/nat_ctrl_60x oil 2_mki.tif]

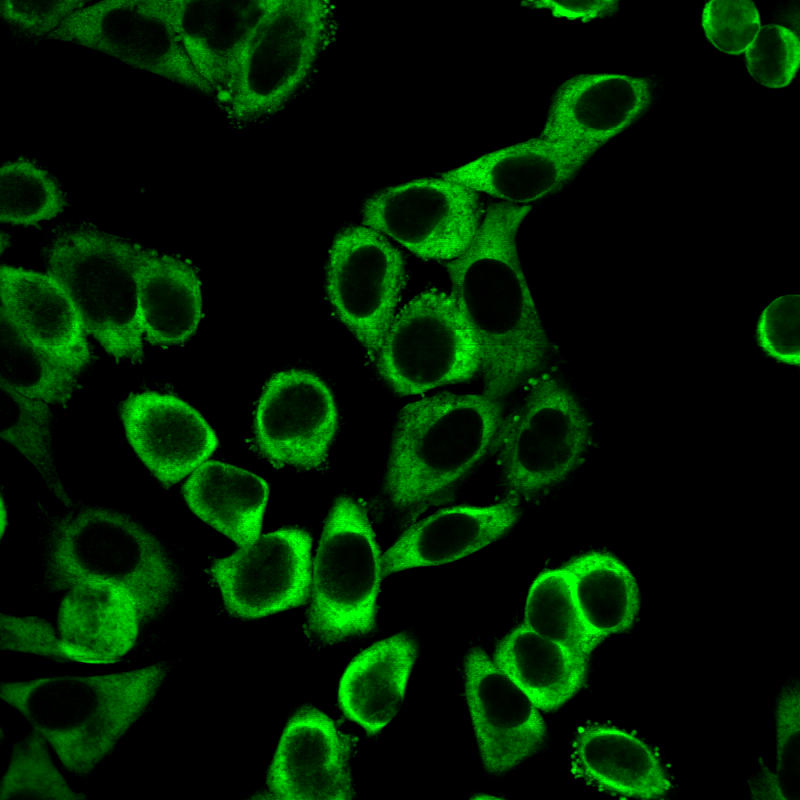

Supplement: Supplementary file 5 — Source Data Fig. 5 [file 44319_2024_98_MOESM5_ESM.zip › 5C/NAT10 KO_0/nat_ctrl_60x oil 2_g3bp.tif]

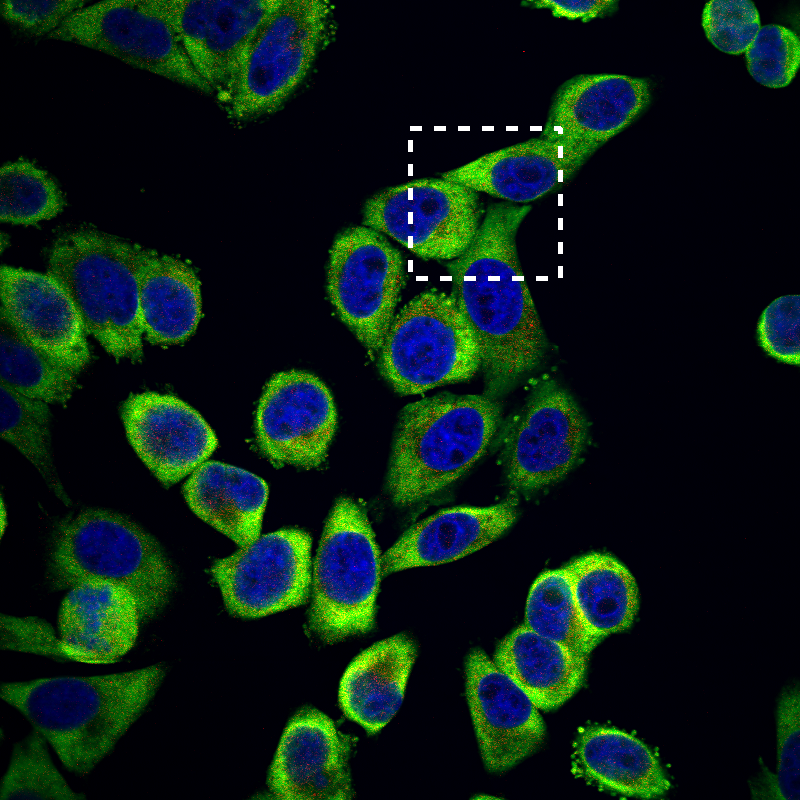

Supplement: Supplementary file 5 — Source Data Fig. 5 [file 44319_2024_98_MOESM5_ESM.zip › 5C/NAT10 KO_0/nat_ctrl_60x oil 2_merged_dashed cropped area.tif]

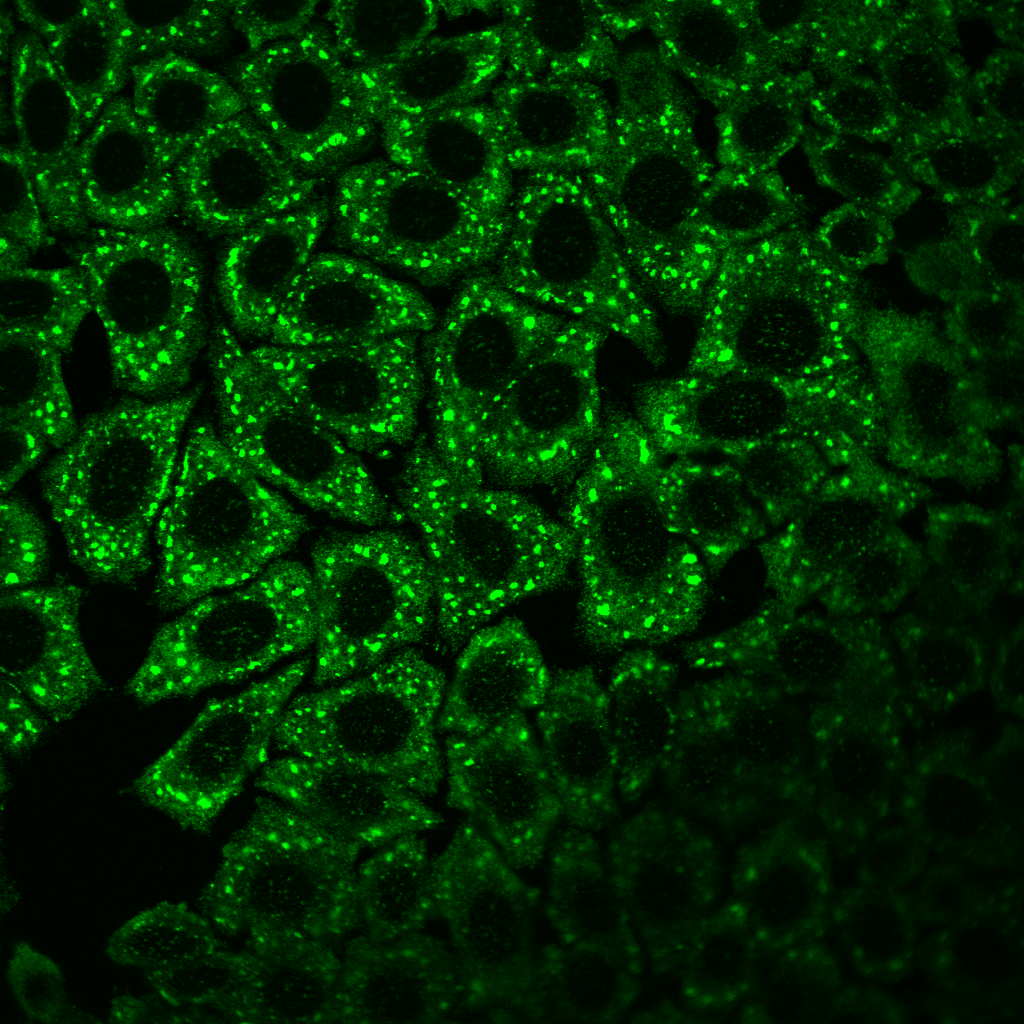

Supplement: Supplementary file 5 — Source Data Fig. 5 [file 44319_2024_98_MOESM5_ESM.zip › 5C/WT_60/wt_60 min_ctrl_60x oil 1_g3bp.tif]

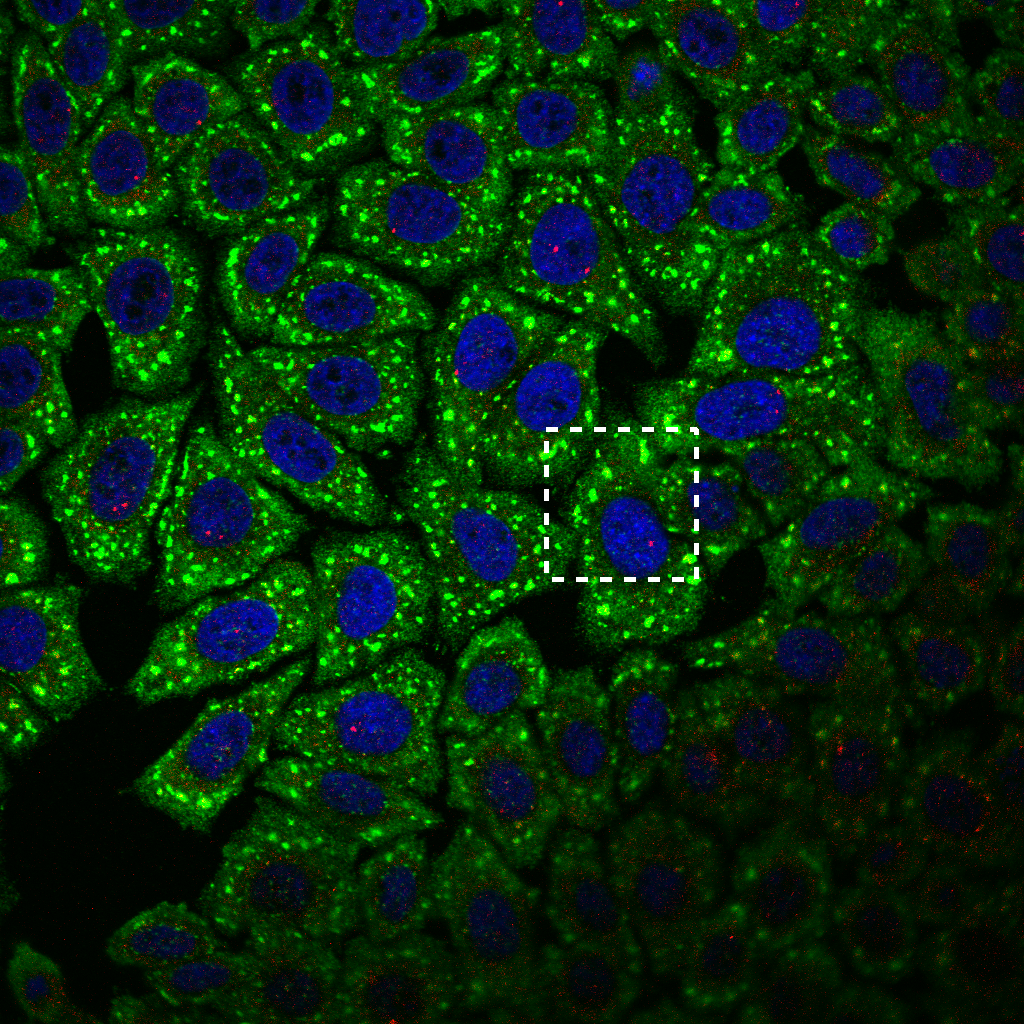

Supplement: Supplementary file 5 — Source Data Fig. 5 [file 44319_2024_98_MOESM5_ESM.zip › 5C/WT_60/wt_60 min_ctrl_60x oil 1_merged_dashed cropped area.tif]

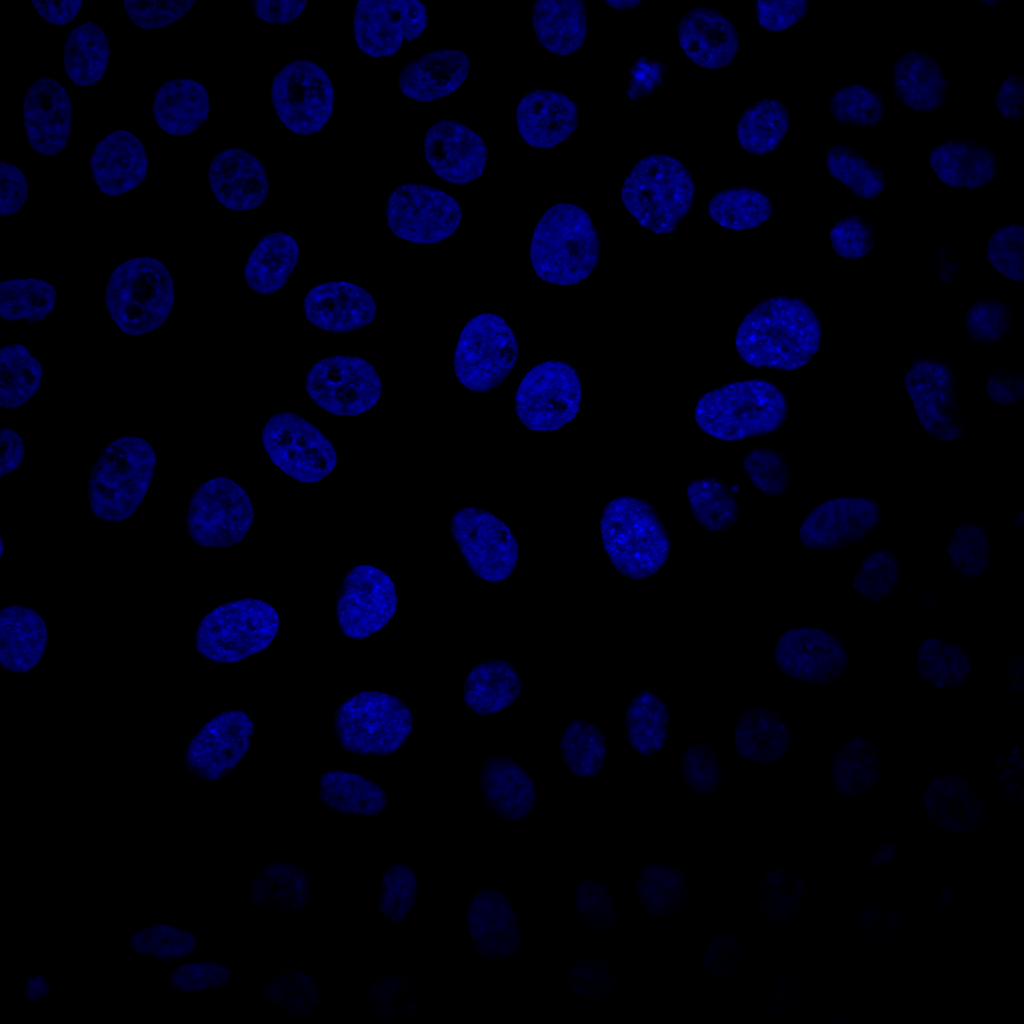

Supplement: Supplementary file 5 — Source Data Fig. 5 [file 44319_2024_98_MOESM5_ESM.zip › 5C/WT_60/wt_60 min_ctrl_60x oil 1_dapi.tif]

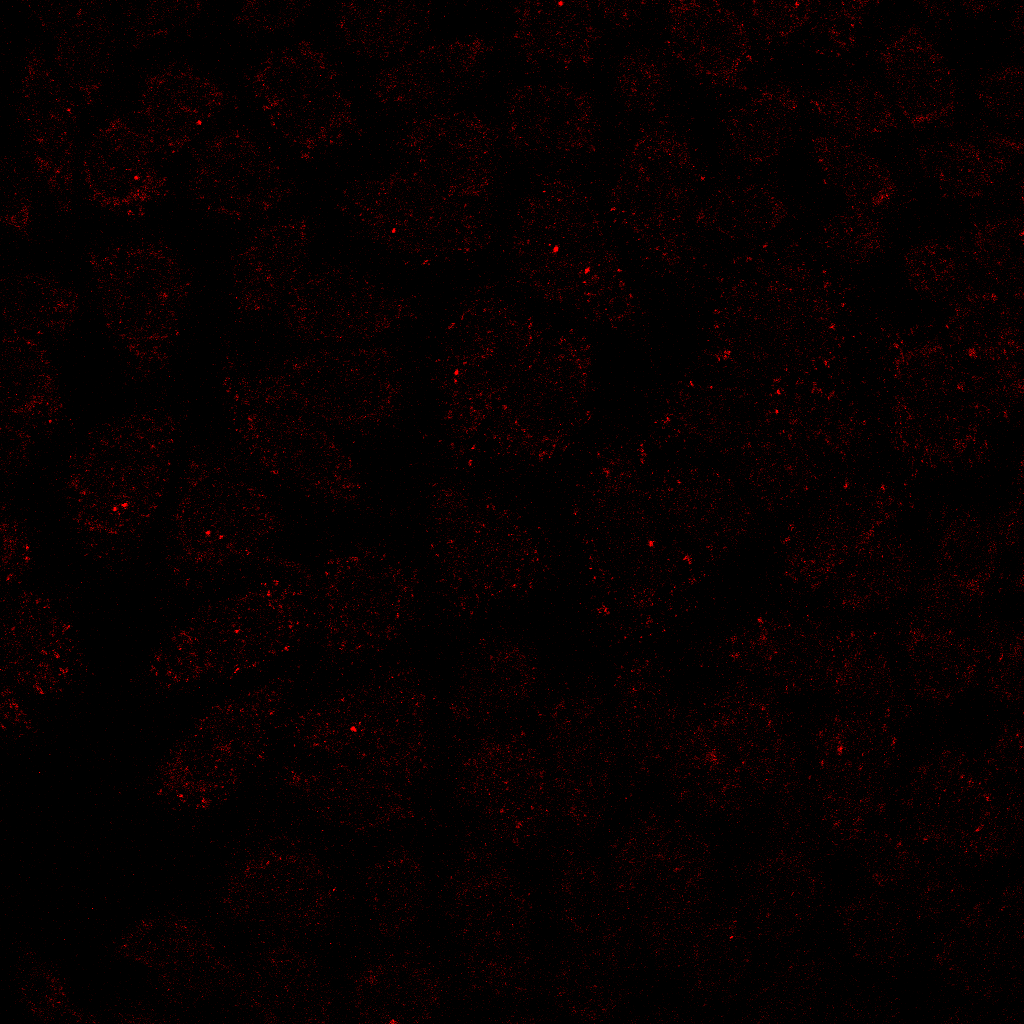

Supplement: Supplementary file 5 — Source Data Fig. 5 [file 44319_2024_98_MOESM5_ESM.zip › 5C/WT_60/wt_60 min_ctrl_60x oil 1_mki.tif]

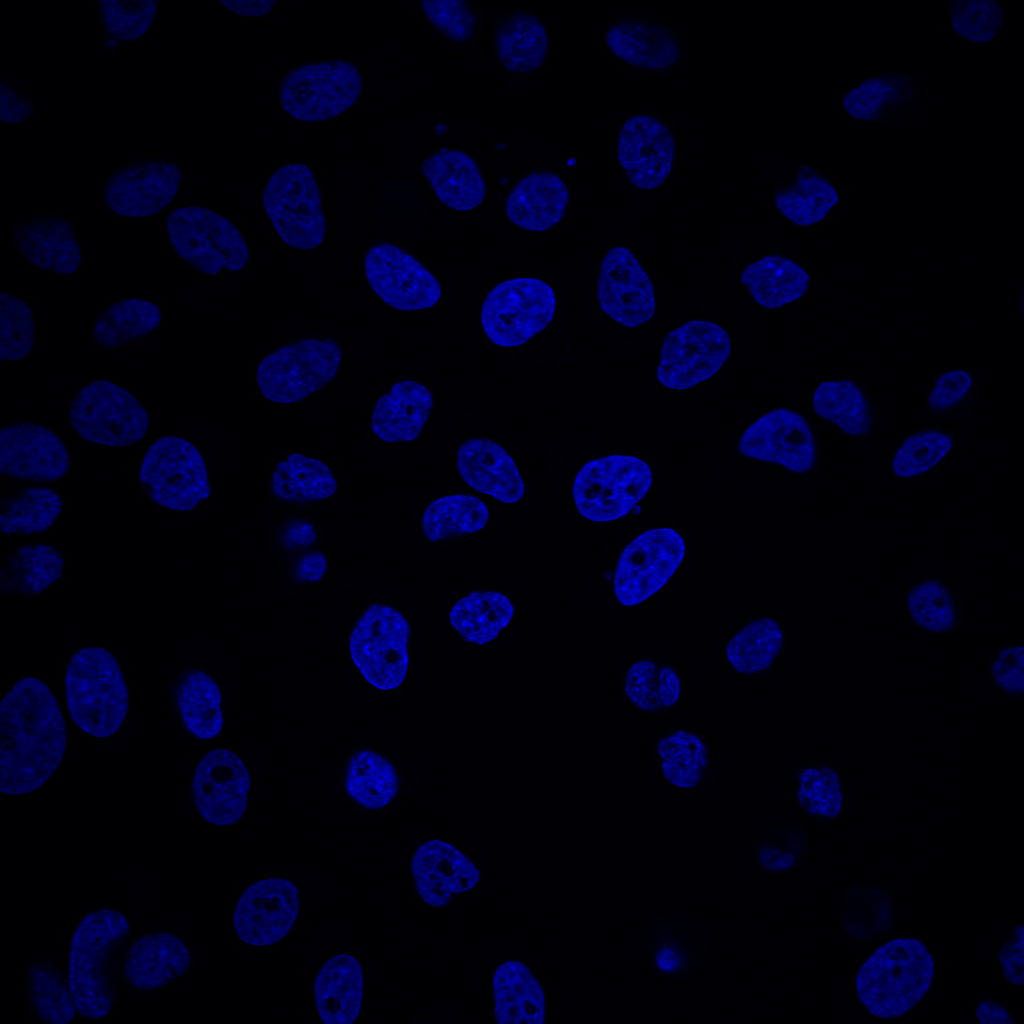

Supplement: Supplementary file 5 — Source Data Fig. 5 [file 44319_2024_98_MOESM5_ESM.zip › 5C/NAT10 KO_30/nat_30 min_ctrl_60x oil 2_dapi.tif]

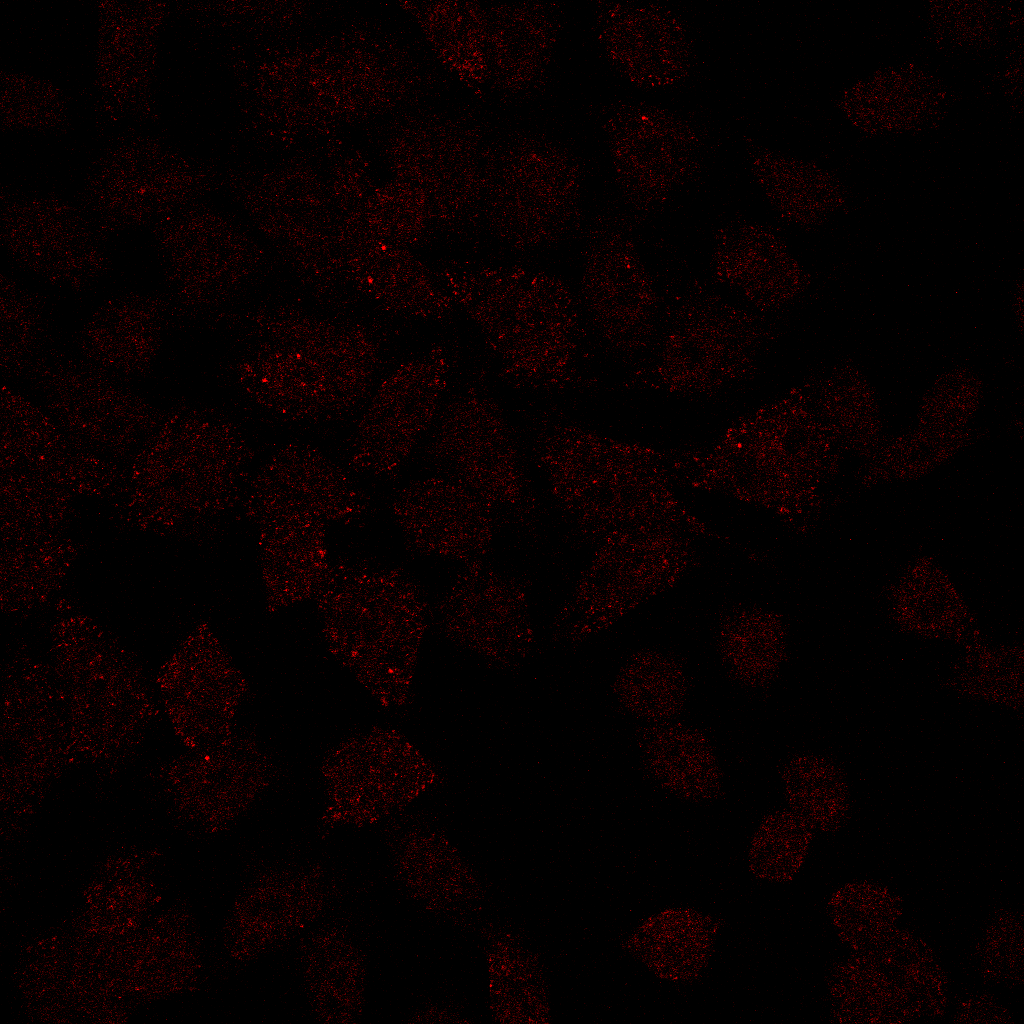

Supplement: Supplementary file 5 — Source Data Fig. 5 [file 44319_2024_98_MOESM5_ESM.zip › 5C/NAT10 KO_30/nat_30 min_ctrl_60x oil 2_mki.tif]

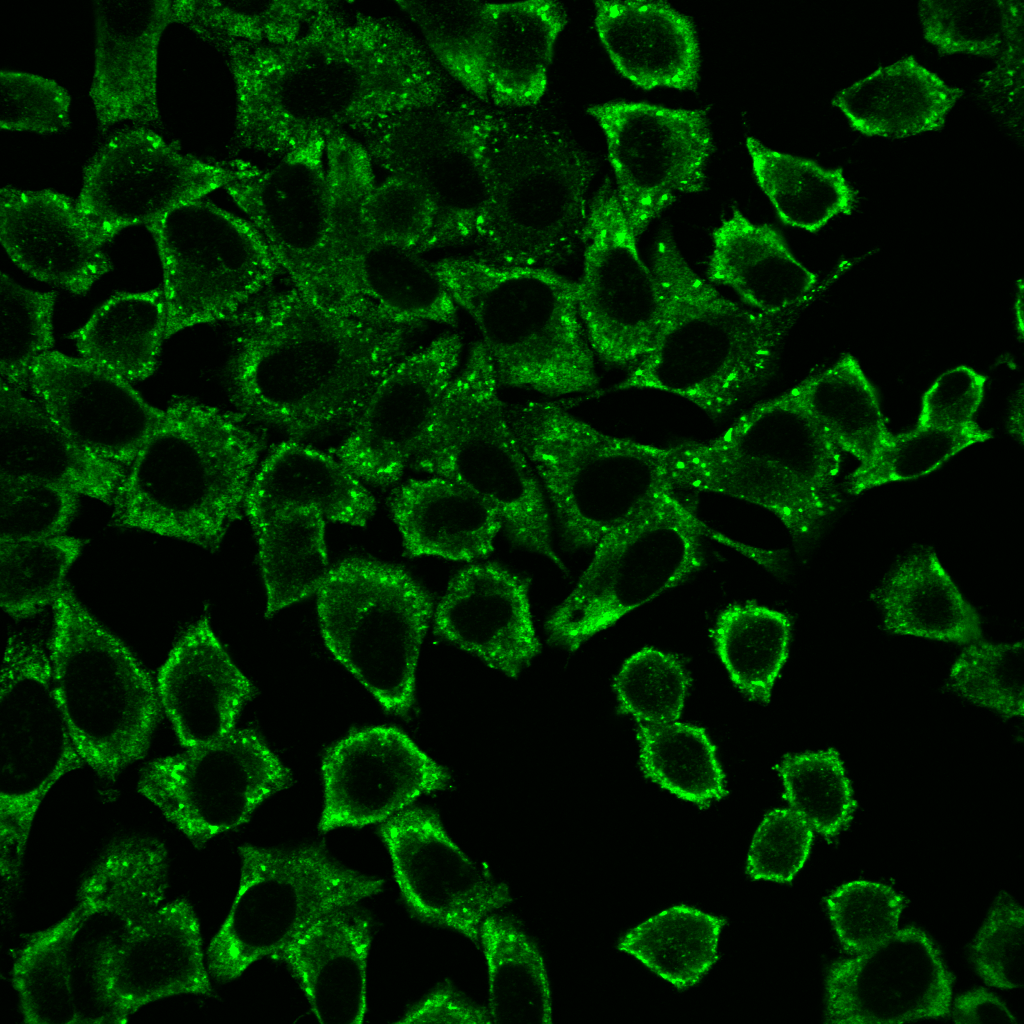

Supplement: Supplementary file 5 — Source Data Fig. 5 [file 44319_2024_98_MOESM5_ESM.zip › 5C/NAT10 KO_30/nat_30 min_ctrl_60x oil 2_g3bp.tif]

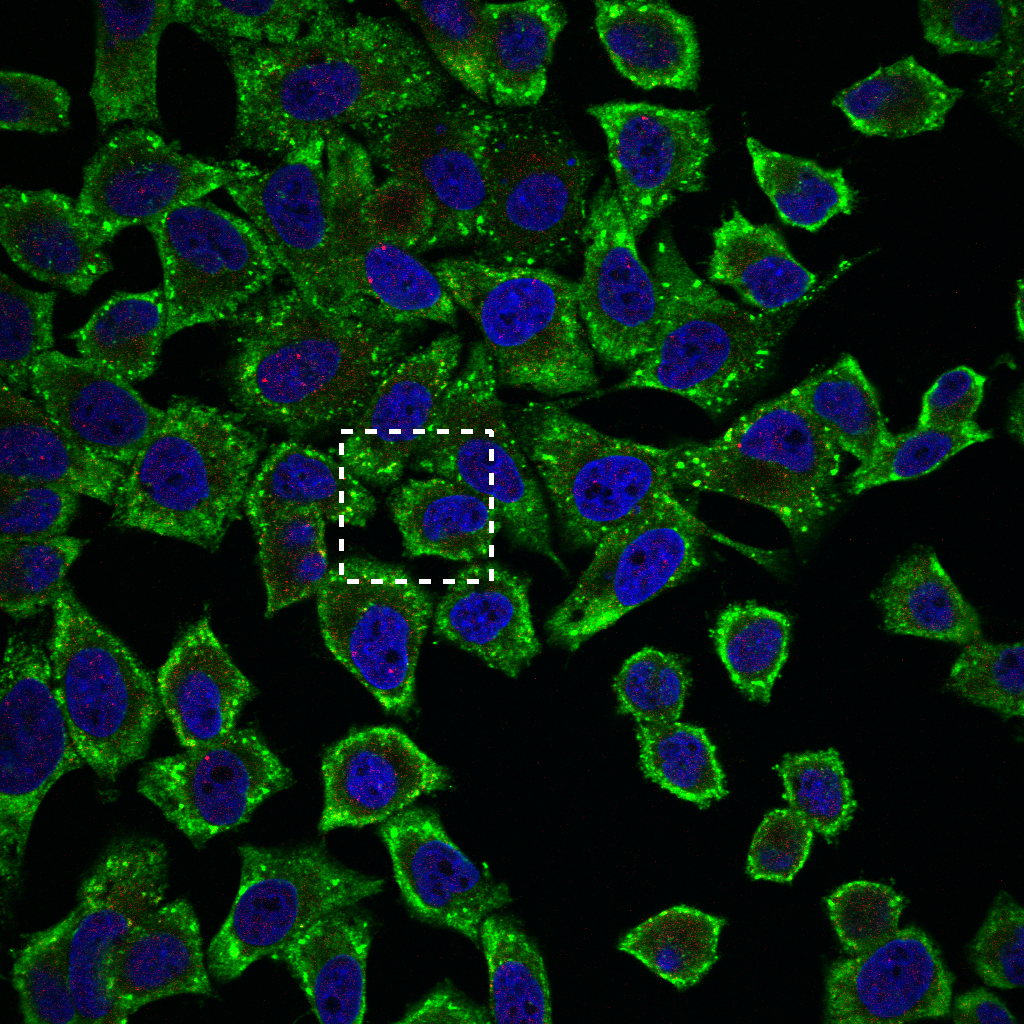

Supplement: Supplementary file 5 — Source Data Fig. 5 [file 44319_2024_98_MOESM5_ESM.zip › 5C/NAT10 KO_30/nat_30 min_ctrl_60x oil 2_merged_dashed cropped area.tif]

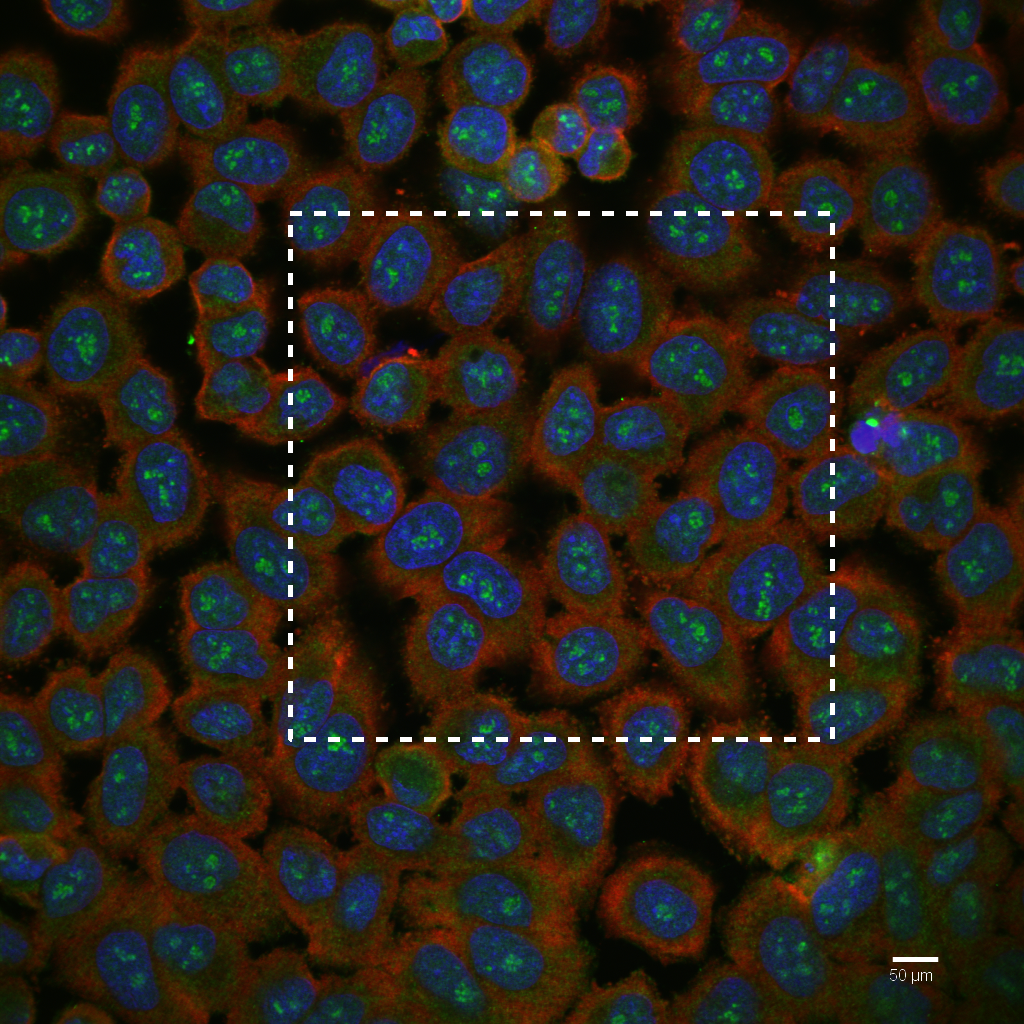

Supplement: Supplementary file 6 — Source Data Fig. 6 [file 44319_2024_98_MOESM6_ESM.zip › 6D/NAT10 KO/nat ctrl 60x 3_merged_dashed cropped area.tif]

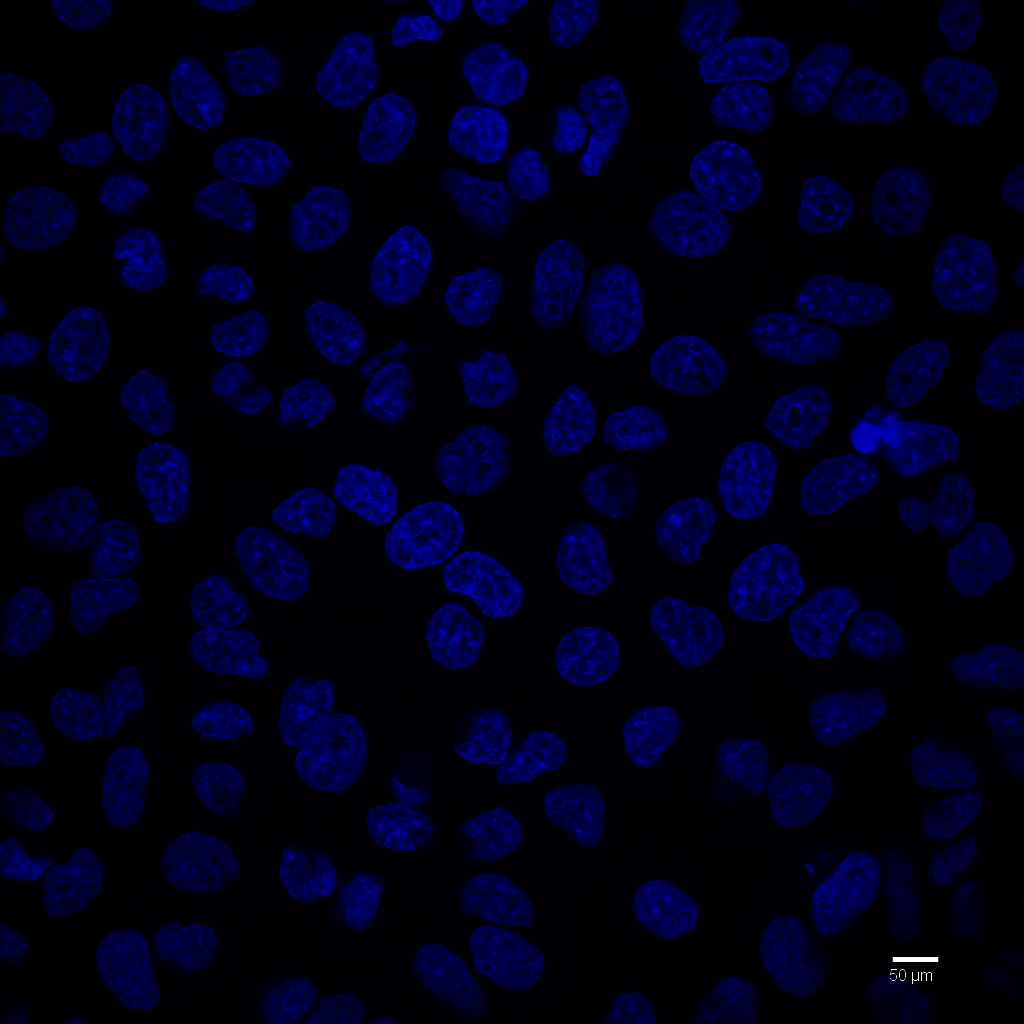

Supplement: Supplementary file 6 — Source Data Fig. 6 [file 44319_2024_98_MOESM6_ESM.zip › 6D/NAT10 KO/nat ctrl 60x 3_dapi.tif]

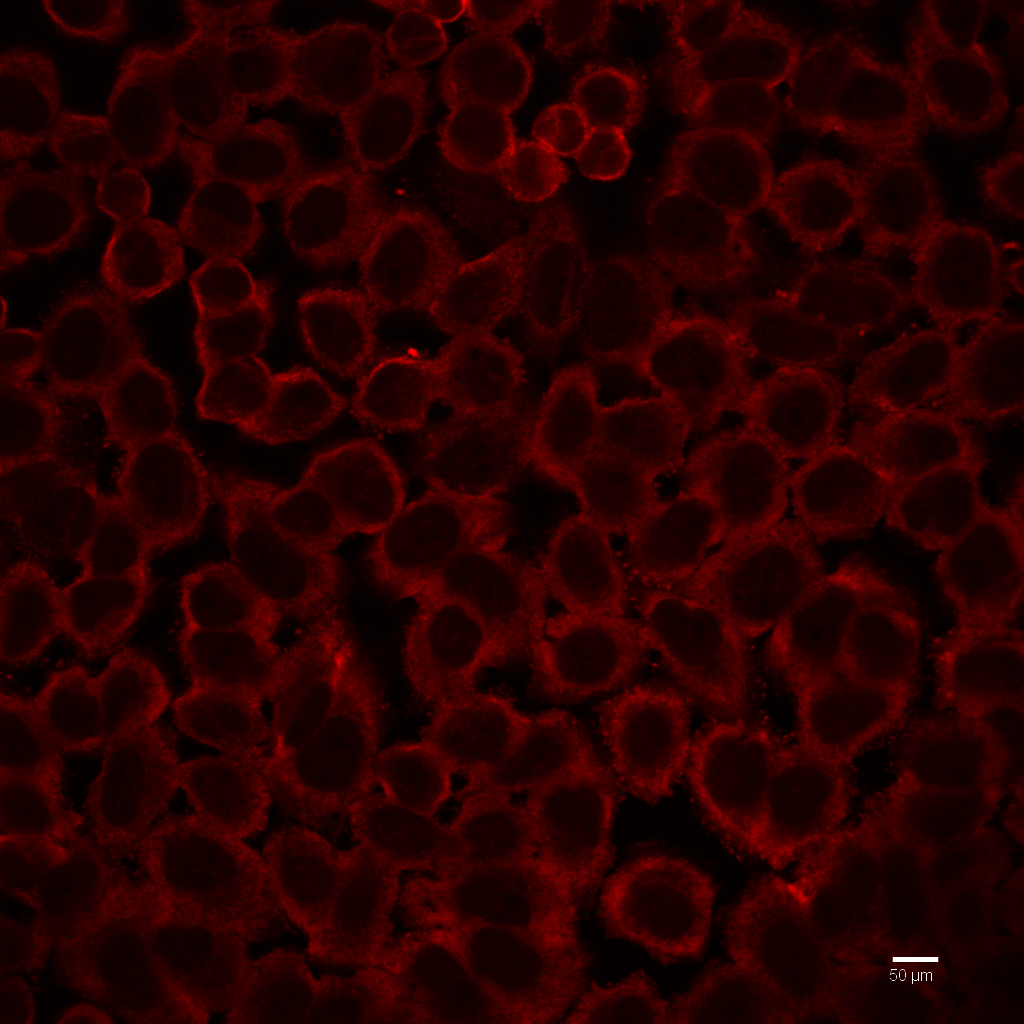

Supplement: Supplementary file 6 — Source Data Fig. 6 [file 44319_2024_98_MOESM6_ESM.zip › 6D/NAT10 KO/nat ctrl 60x 3_g3bp.tif]

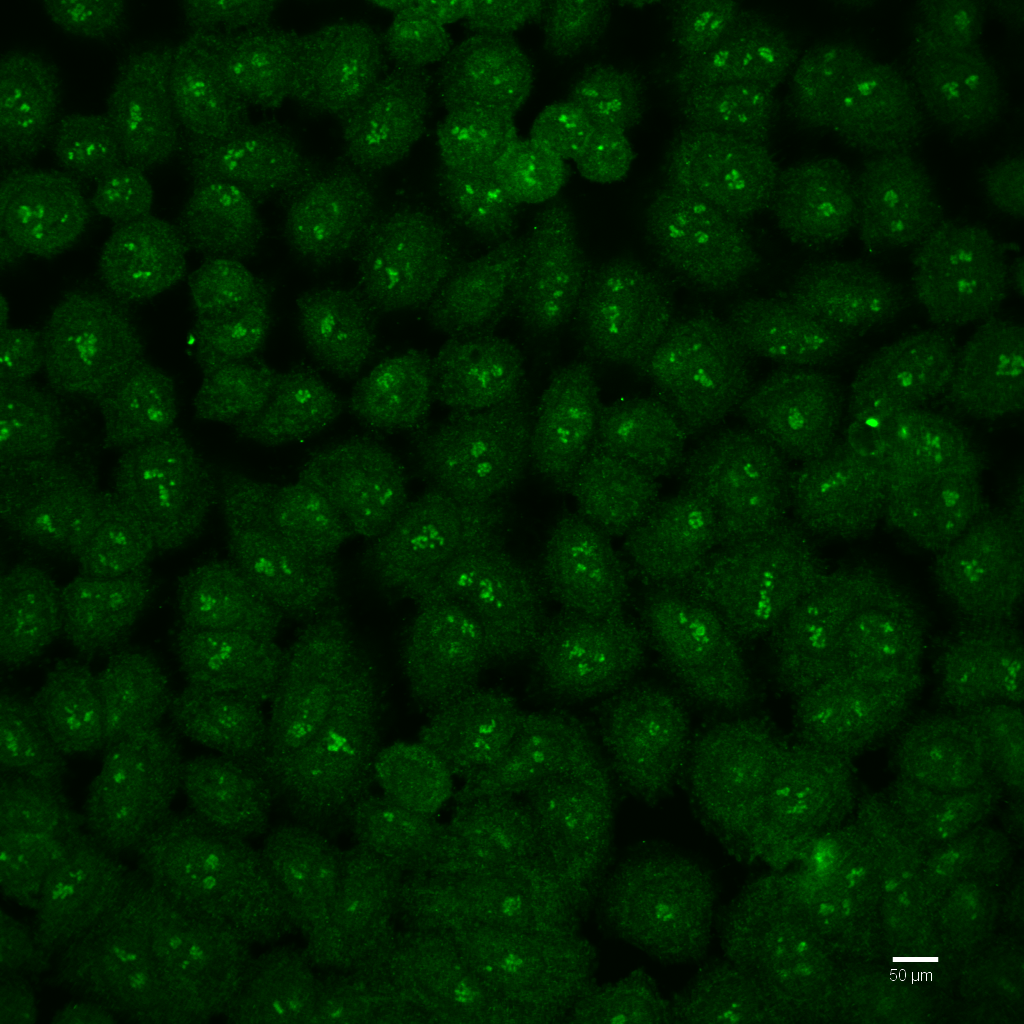

Supplement: Supplementary file 6 — Source Data Fig. 6 [file 44319_2024_98_MOESM6_ESM.zip › 6D/NAT10 KO/nat ctrl 60x 3_nop58.tif]

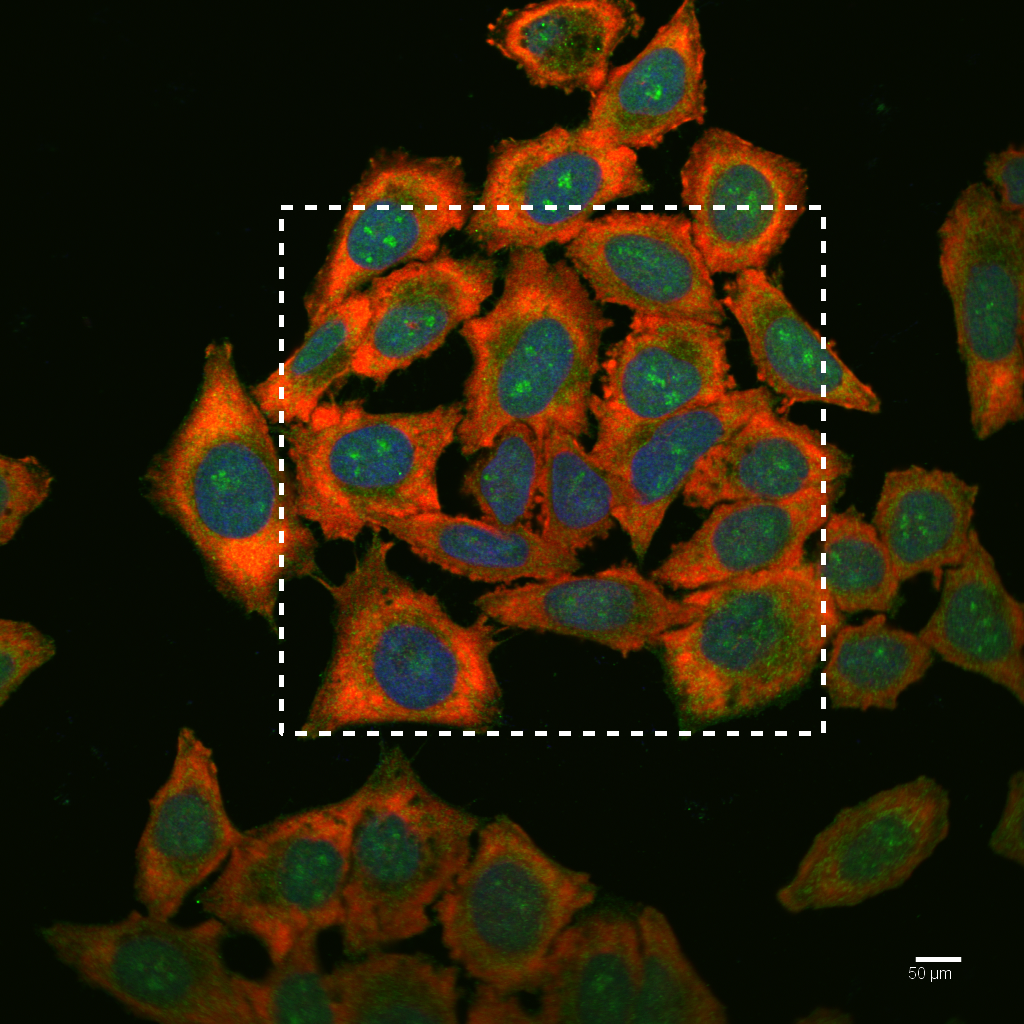

Supplement: Supplementary file 6 — Source Data Fig. 6 [file 44319_2024_98_MOESM6_ESM.zip › 6D/WT/wt ctrl 60x 3_merged_dashed cropped area.tif]

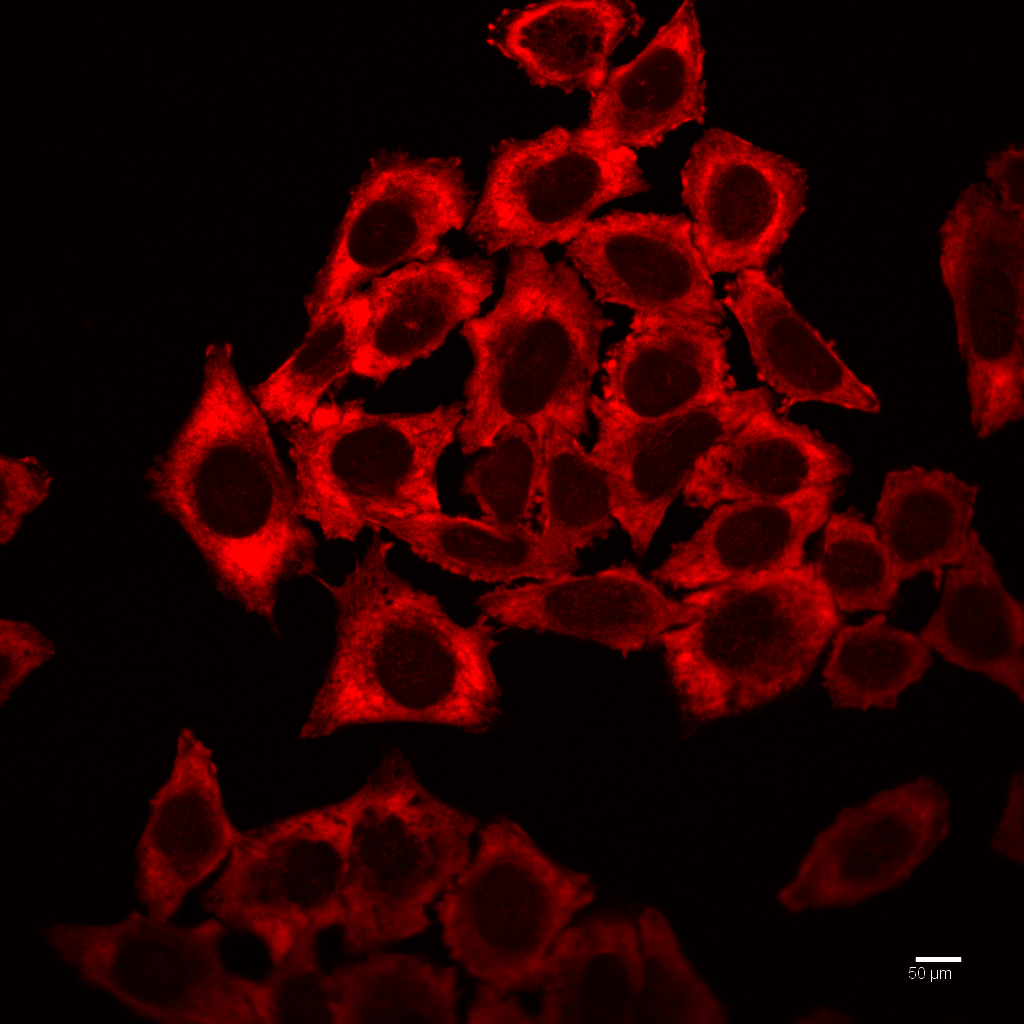

Supplement: Supplementary file 6 — Source Data Fig. 6 [file 44319_2024_98_MOESM6_ESM.zip › 6D/WT/wt ctrl 60x 3_g3bp.tif]

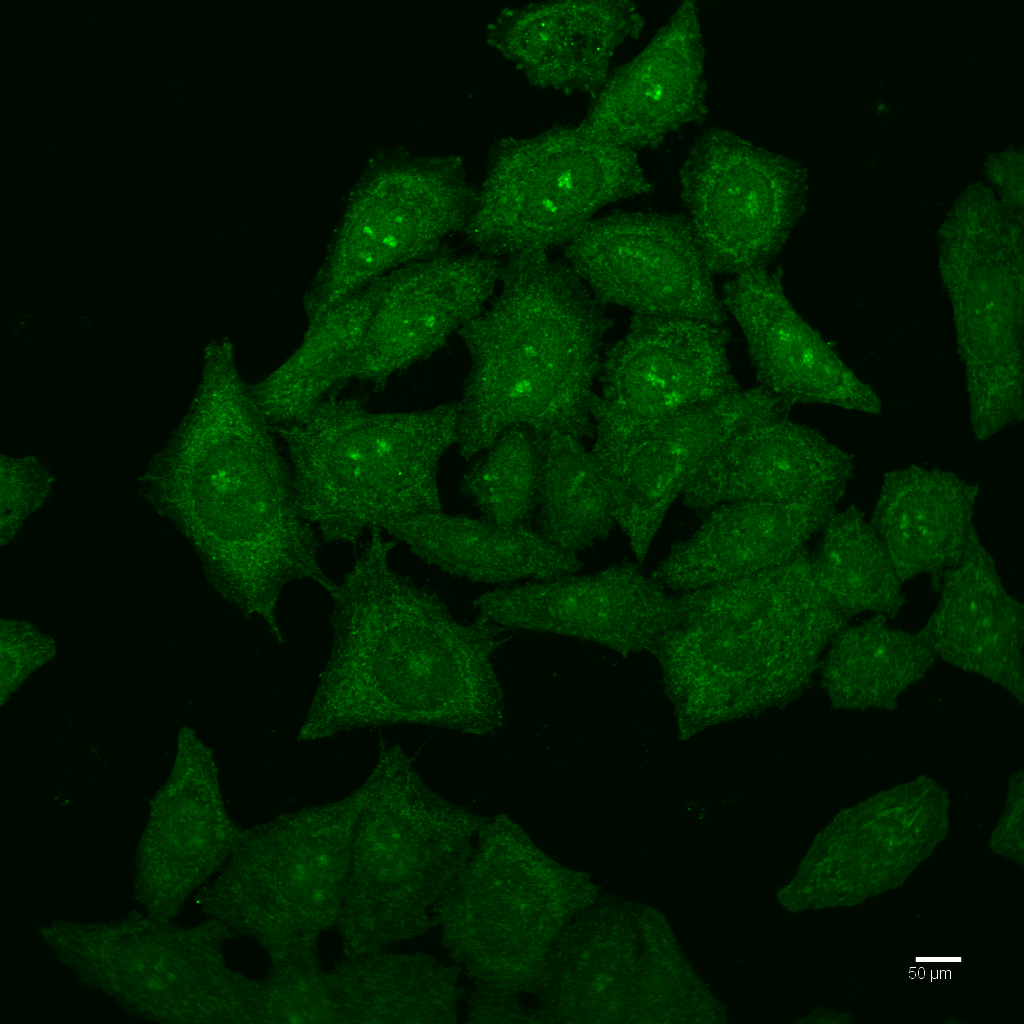

Supplement: Supplementary file 6 — Source Data Fig. 6 [file 44319_2024_98_MOESM6_ESM.zip › 6D/WT/wt ctrl 60x 3_nop58.tif]

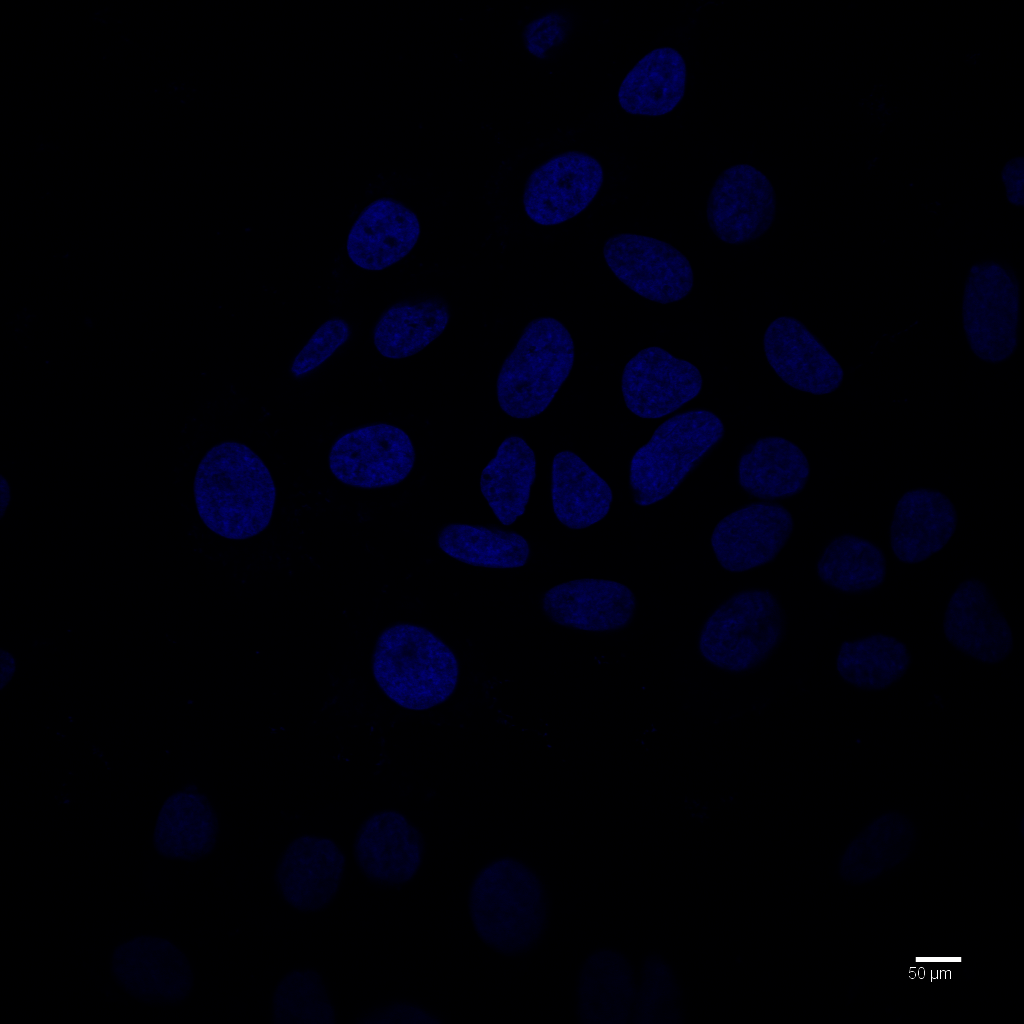

Supplement: Supplementary file 6 — Source Data Fig. 6 [file 44319_2024_98_MOESM6_ESM.zip › 6D/WT/wt ctrl 60x 3_dapi.tif]

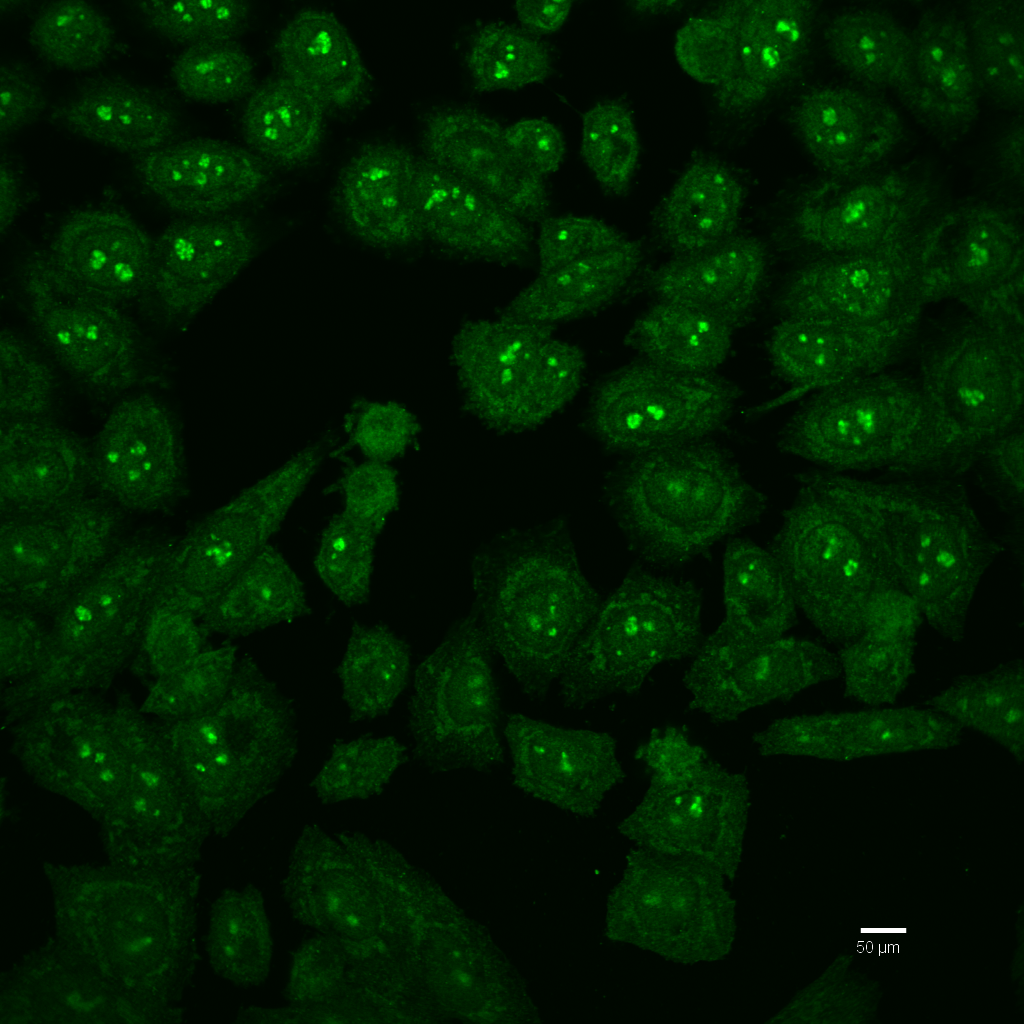

Supplement: Supplementary file 6 — Source Data Fig. 6 [file 44319_2024_98_MOESM6_ESM.zip › 6E/NAT10 KO/nat sg 60x 5_nop58.tif]

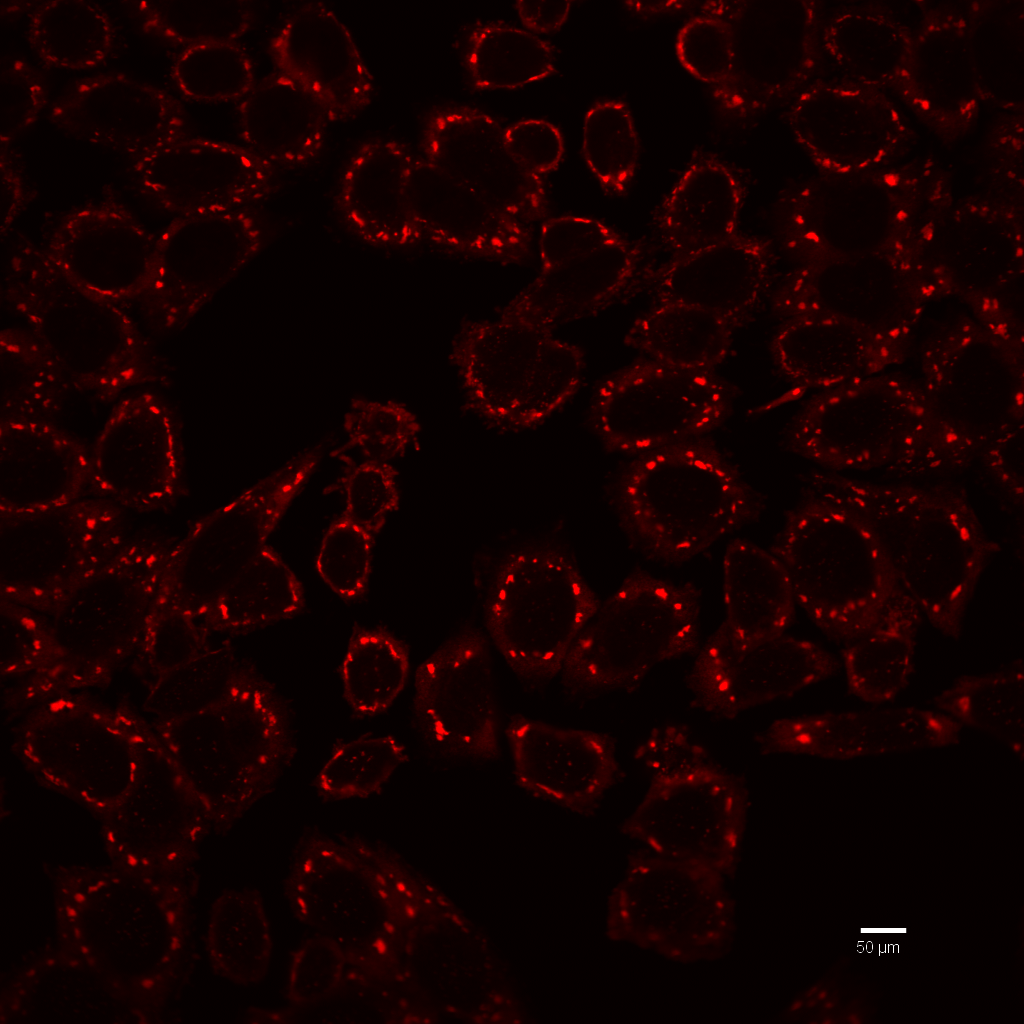

Supplement: Supplementary file 6 — Source Data Fig. 6 [file 44319_2024_98_MOESM6_ESM.zip › 6E/NAT10 KO/nat sg 60x 5_g3bp.tif]

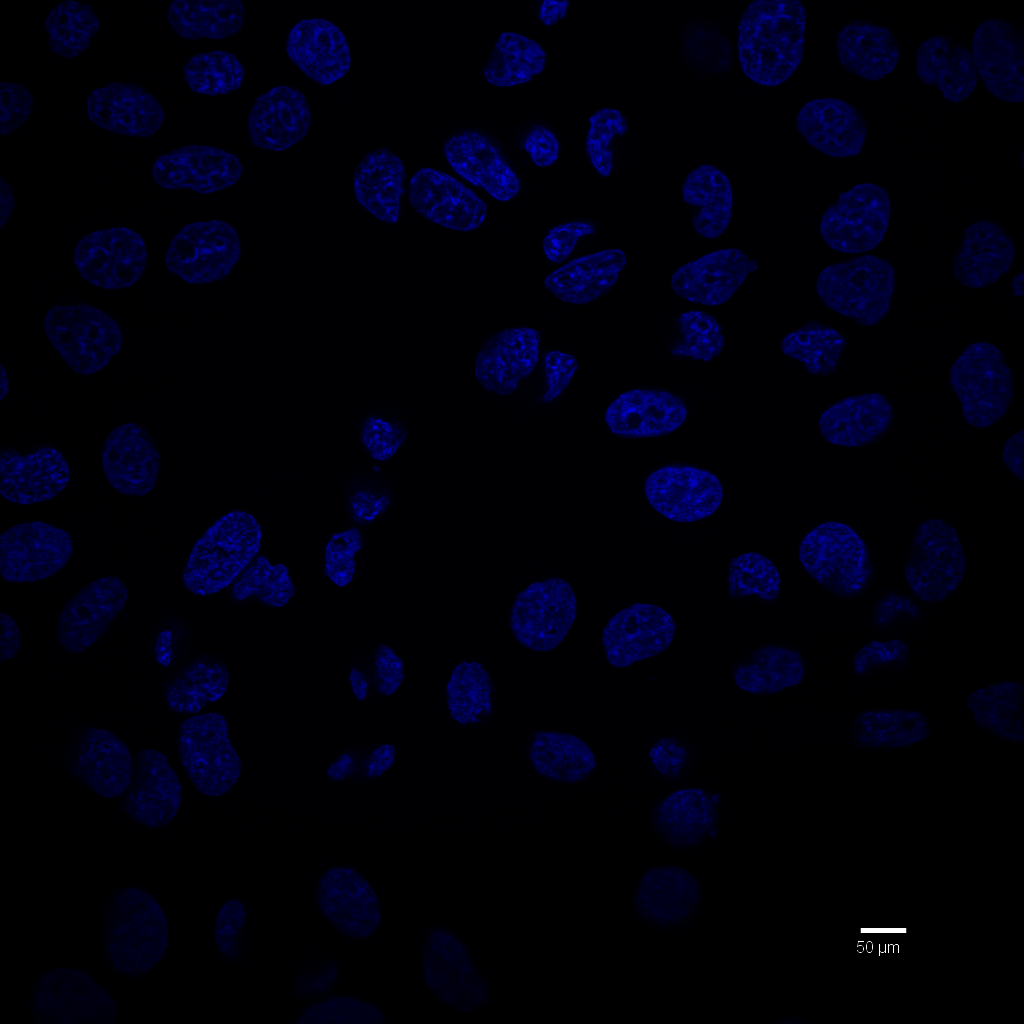

Supplement: Supplementary file 6 — Source Data Fig. 6 [file 44319_2024_98_MOESM6_ESM.zip › 6E/NAT10 KO/nat sg 60x 5_dapi.tif]

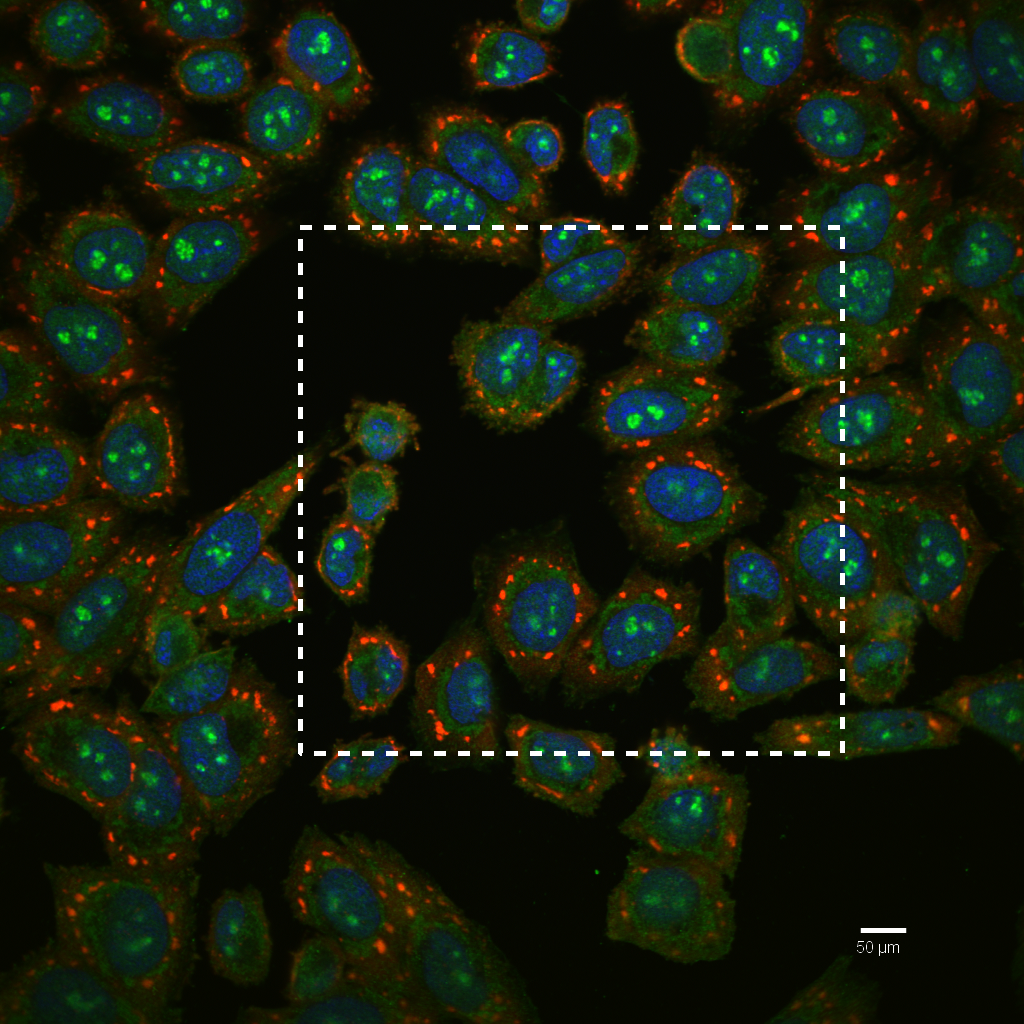

Supplement: Supplementary file 6 — Source Data Fig. 6 [file 44319_2024_98_MOESM6_ESM.zip › 6E/NAT10 KO/nat sg 60x 5_merged_dashed cropped area.tif]

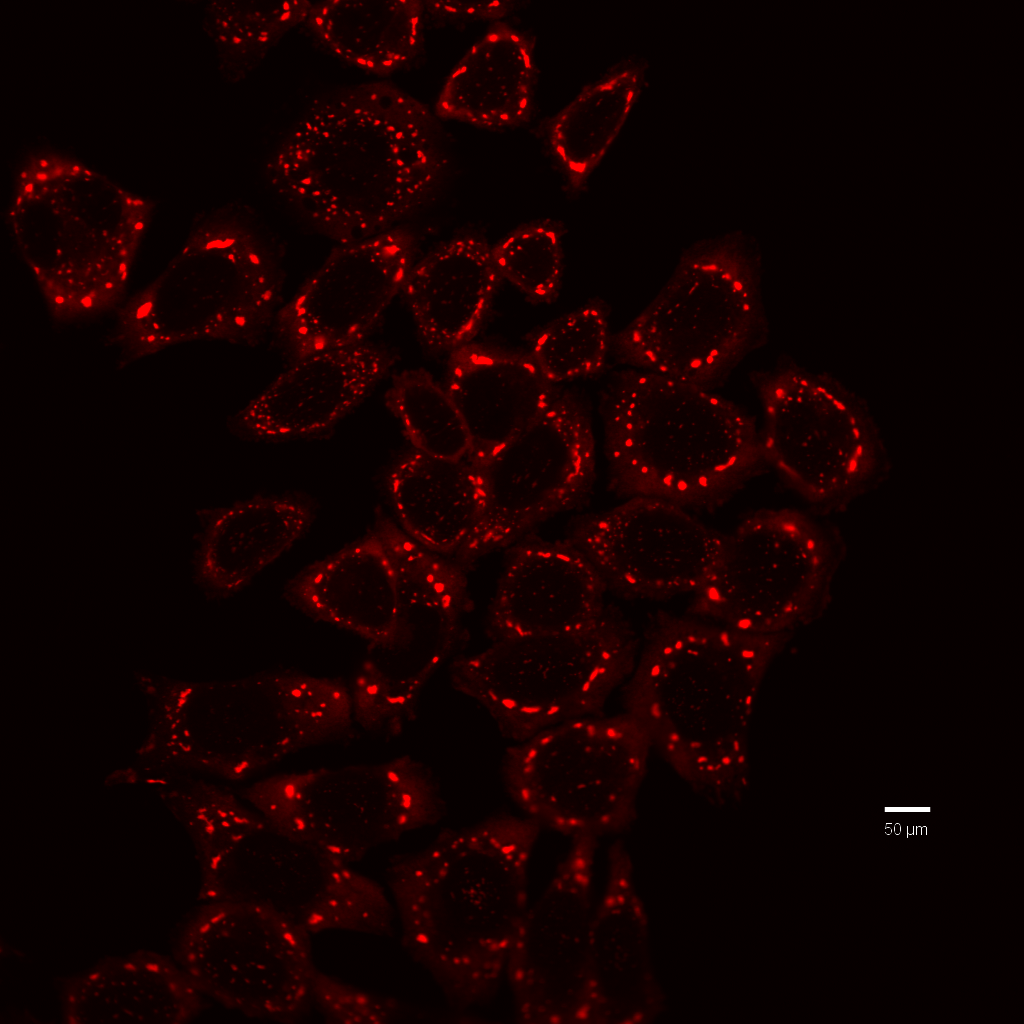

Supplement: Supplementary file 6 — Source Data Fig. 6 [file 44319_2024_98_MOESM6_ESM.zip › 6E/WT/wt sg 60x 4_g3bp.tif]

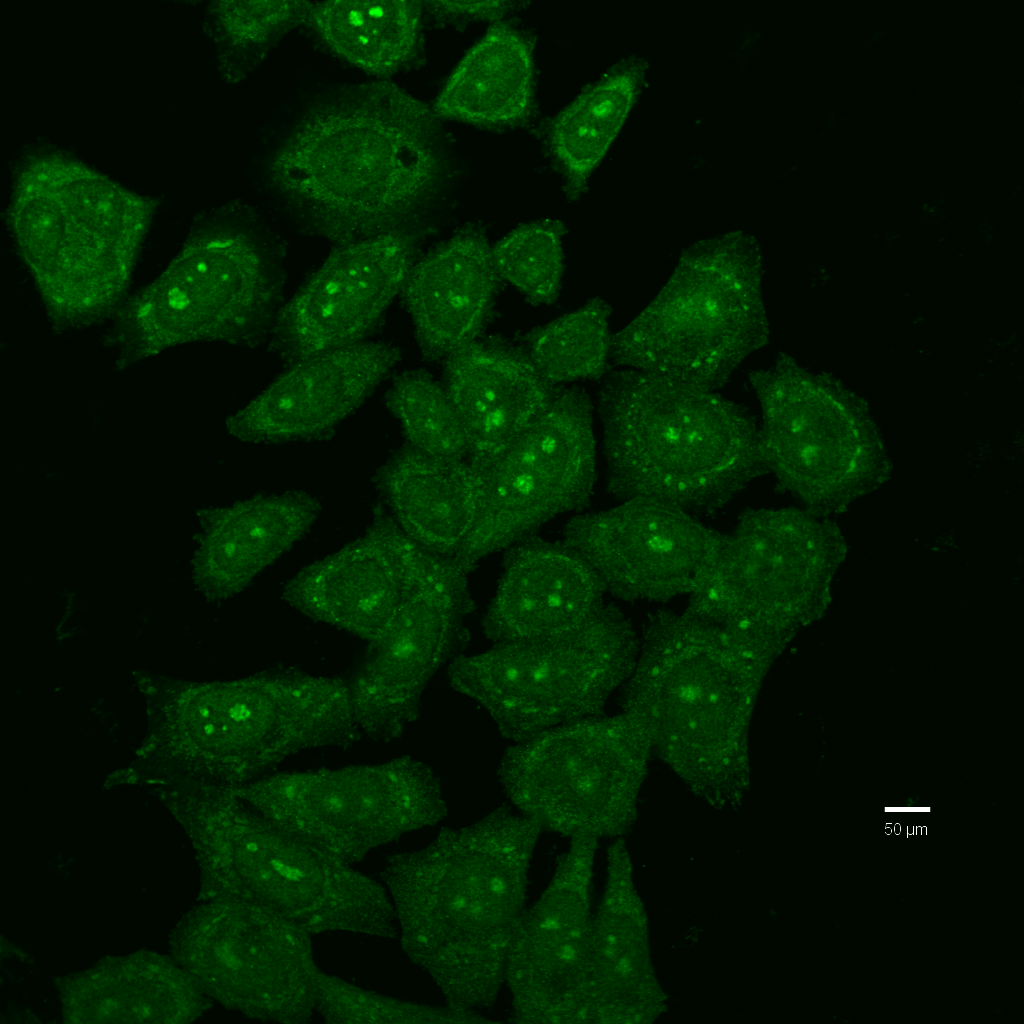

Supplement: Supplementary file 6 — Source Data Fig. 6 [file 44319_2024_98_MOESM6_ESM.zip › 6E/WT/wt sg 60x 4_nop58.tif]

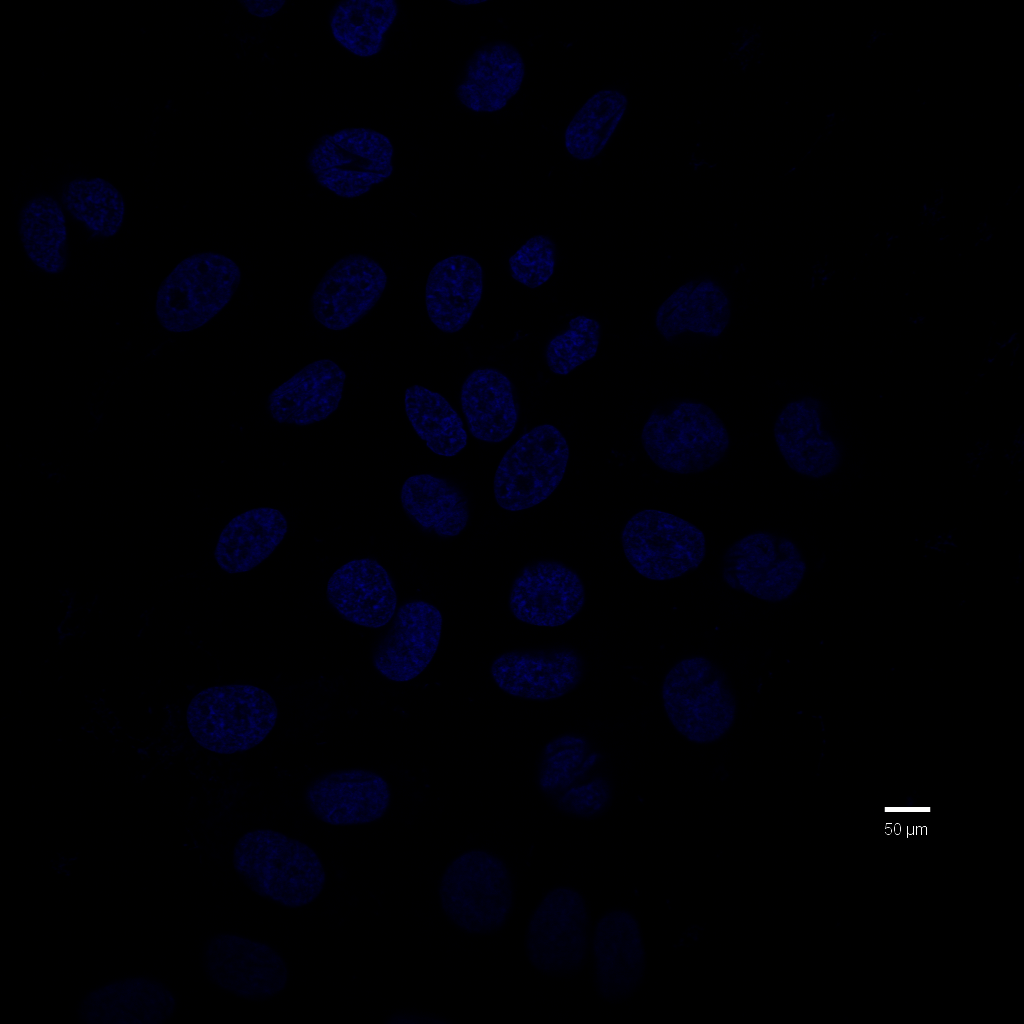

Supplement: Supplementary file 6 — Source Data Fig. 6 [file 44319_2024_98_MOESM6_ESM.zip › 6E/WT/wt sg 60x 4_dapi.tif]

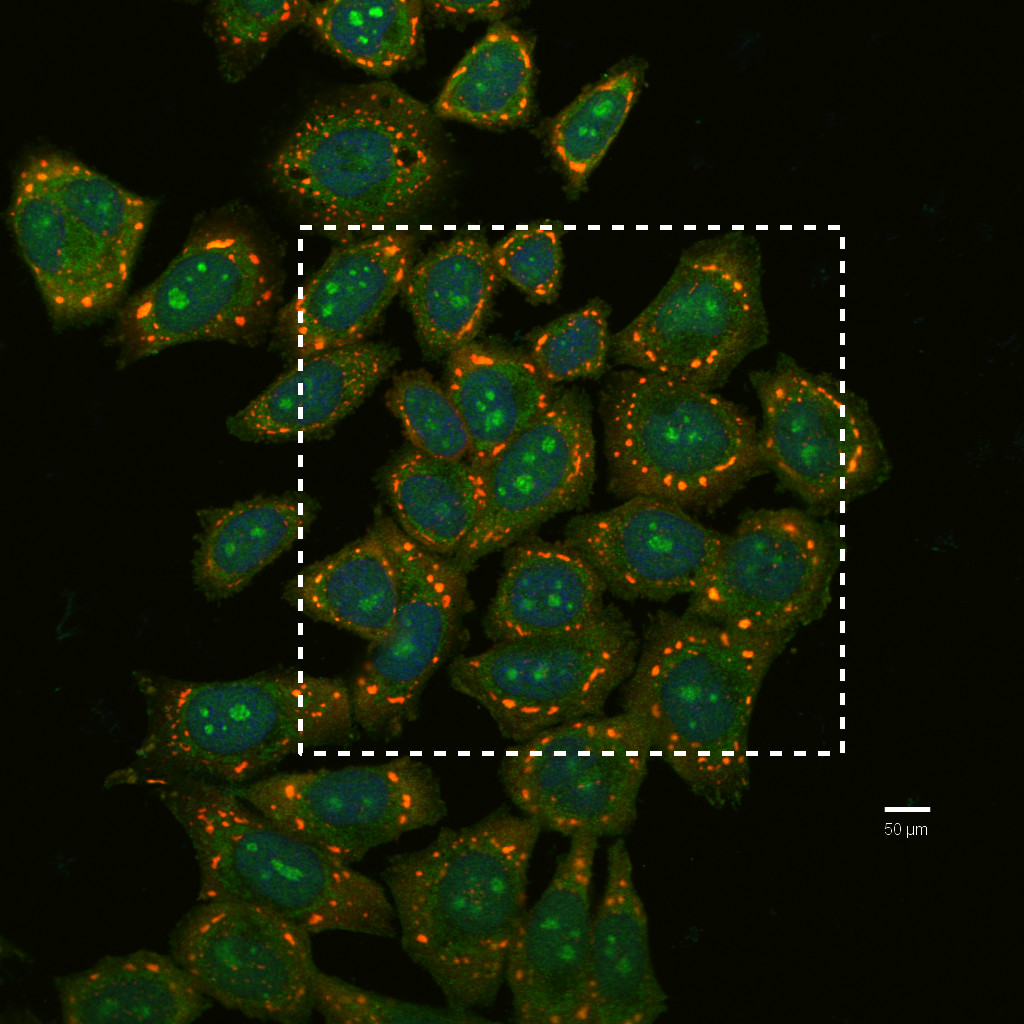

Supplement: Supplementary file 6 — Source Data Fig. 6 [file 44319_2024_98_MOESM6_ESM.zip › 6E/WT/wt sg 60x 4_merged_dashed cropped area.tif]

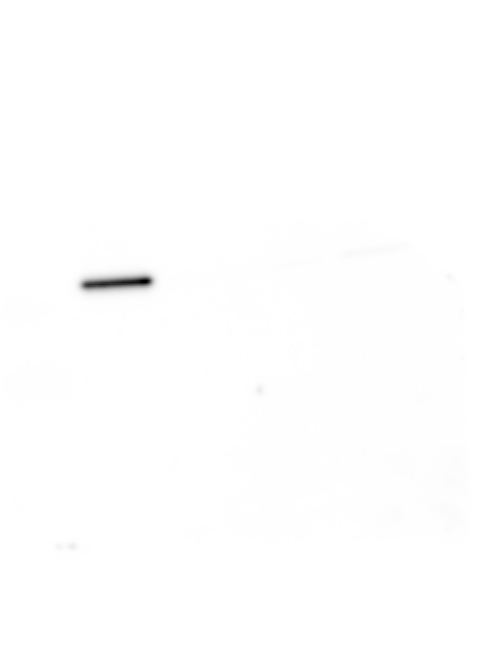

Supplement: Supplementary file 6 — Source Data Fig. 6 [file 44319_2024_98_MOESM6_ESM.zip › 6B/gapdh-2020.12.14_14.24.11_Ch.tif]

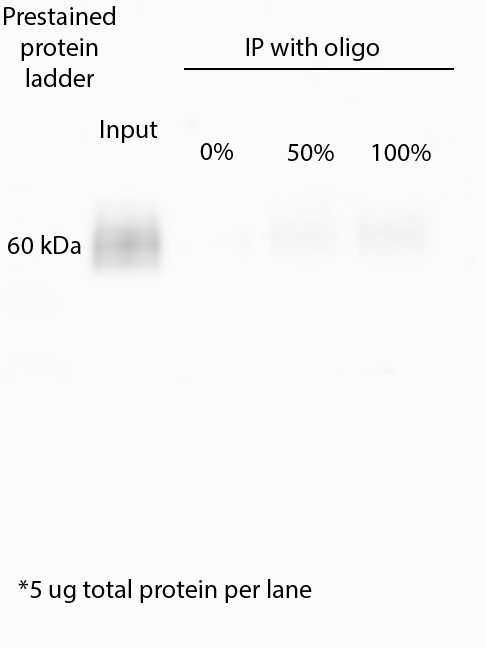

Supplement: Supplementary file 6 — Source Data Fig. 6 [file 44319_2024_98_MOESM6_ESM.zip › 6B/nop58-1 2020.12.14_14.19.28_annotated.tif]

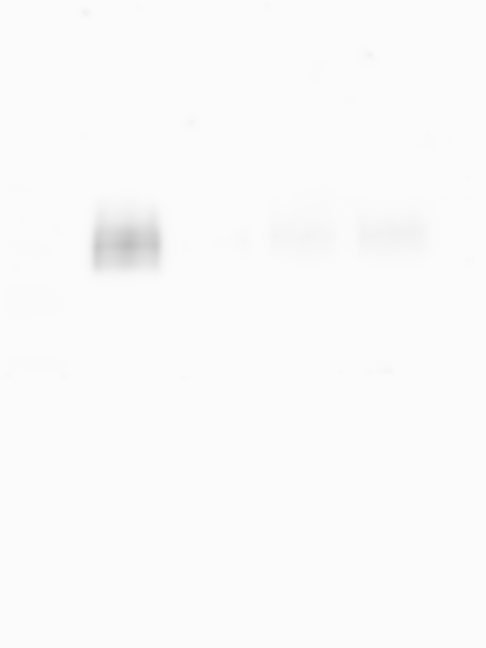

Supplement: Supplementary file 6 — Source Data Fig. 6 [file 44319_2024_98_MOESM6_ESM.zip › 6B/nop58-1 2020.12.14_14.19.28_Ch.tif]

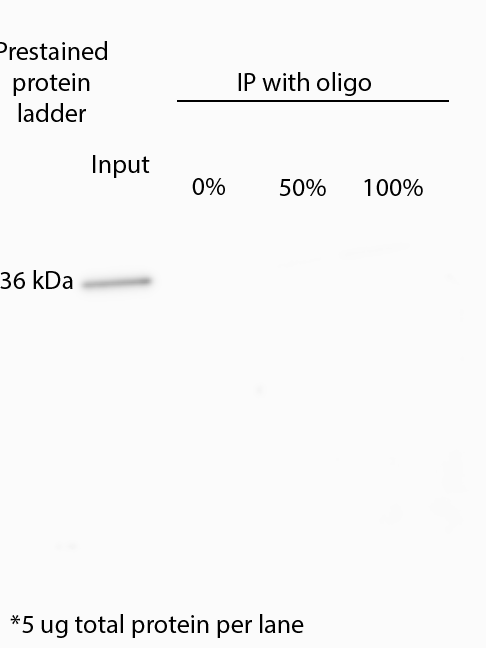

Supplement: Supplementary file 6 — Source Data Fig. 6 [file 44319_2024_98_MOESM6_ESM.zip › 6B/gapdh-2020.12.14_14.24.11_annotated.tif]
