# Supplementary material for: N4-acetylcytidine (ac4C) promotes mRNA localization to stress granules
Source: EMBO Rep. 2024 Feb 27;25(4):1814–34. doi: 10.1038/s44319-024-00098-6 (PMC11014937; doi:10.1038/s44319-024-00098-6)
Supplement: Supplementary file 8 — Expanded View Figures [file 44319_2024_98_MOESM8_ESM.pdf]

Expanded View Figures

Figure EV1. Determination of ac4C RNA modification level in stressed HeLa cells.

(**A**) ac4C-rich RNAs localize in G3BP- and PABP-containing stress granules (SG) in response to oxidative and osmotic stresses, caused by sodium arsenite and d-sorbitol treatments, respectively. Individual SG are indicated by arrows. For panels (**A**,**D**,**E**) ac4C is depicted in green, PABP, G3BP or NCL in red, DAPI staining (nuclei) is in blue, scale bar 10 µm; (**B**) RNA mass spectrometry (RNA MS) analysis shows strong decrease in ac4C level in NAT10 KO HeLa cells. $n = 3$ biological replicates, data represented as Mean ± SD. Unpaired two-tailed *t*-test, ***$p < 0.001$; (**C**) Reduction in NAT10 protein level is confirmed by western blot; (**D**) Decreased ac4C IF signal in NAT10 KO cells suggests decreased ac4C levels in nucleoli of untreated and SG of arsenite-stressed cells. Individual organelles are indicated by arrows; (**E**) ac4C IF signal disappears from SG of arsenite-stressed cells in response to RNase A treatment. The levels of RNA modifications m6a, ac4C and m7G in (**F**) SG, (**G**) poly(A) RNA, (**H**) total RNA, (**I**) 18S rRNA and (**J**) tRNA from WT HeLa cells measured by RNA MS. Experiments are done in 2 replicates for panel (**G**) and three replicates for panels (**F**, **H–J**), data represented as Mean ± SD.

▶

© The Author(s)

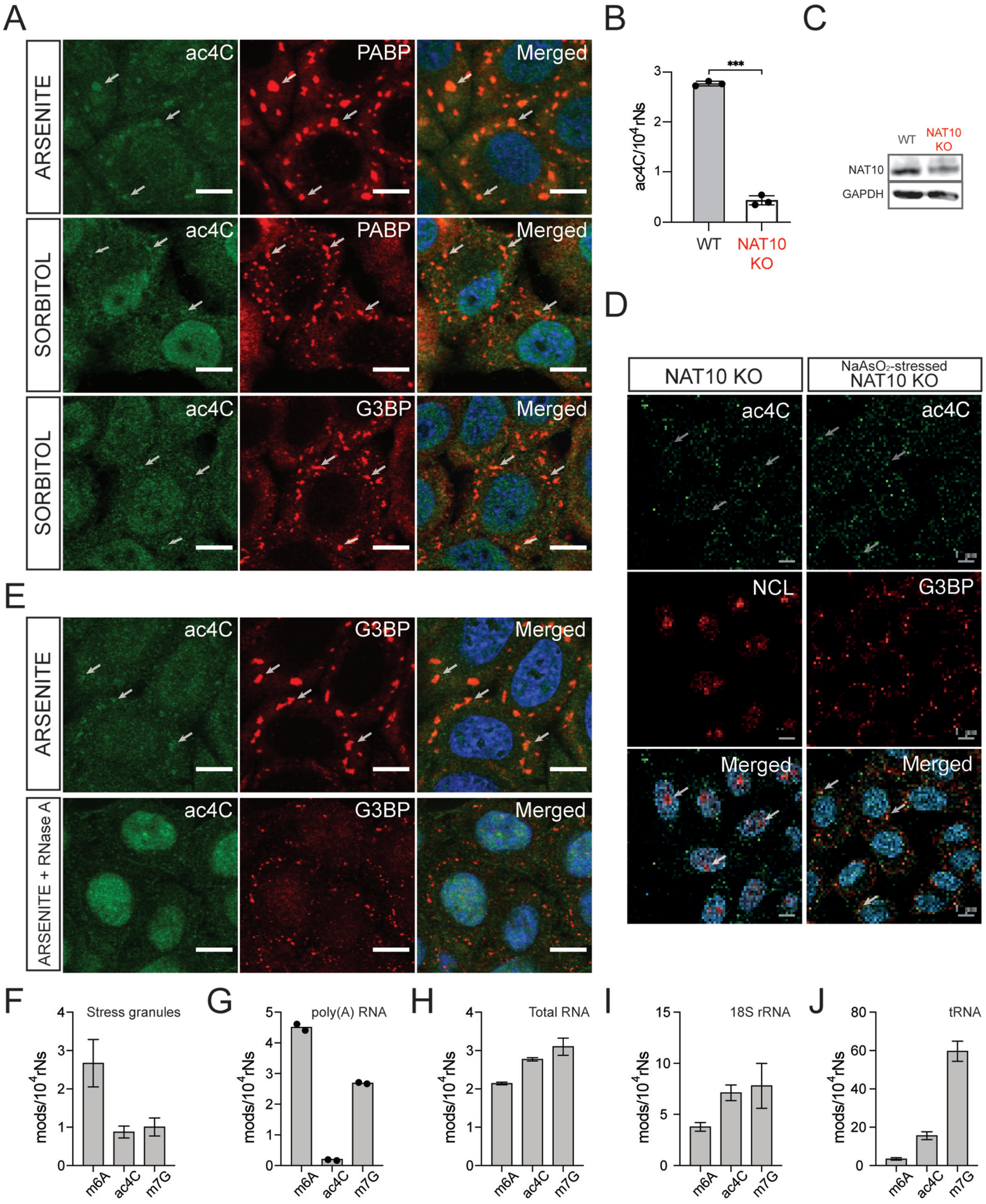
A
ARSENITE
SORBITOL
SORBITOL
ac4C
PABP
G3BP
Merged
B
ac4C/10^{4}rNs
***
WT
NAT10 KO
C
WT
NAT10 KO
NAT10
GAPDH
D
NAT10 KO
NaAsO2-stressed NAT10 KO
ac4C
NCL
G3BP
Merged
E
ARSENITE
ARSENITE + RNase A
ac4C
G3BP
Merged
F
Stress granules
G
poly(A) RNA
H
Total RNA
I
18S rRNA
J
tRNA
mods/10^{4}rNs
m6A
ac4C
m7G

 © The Author(s)

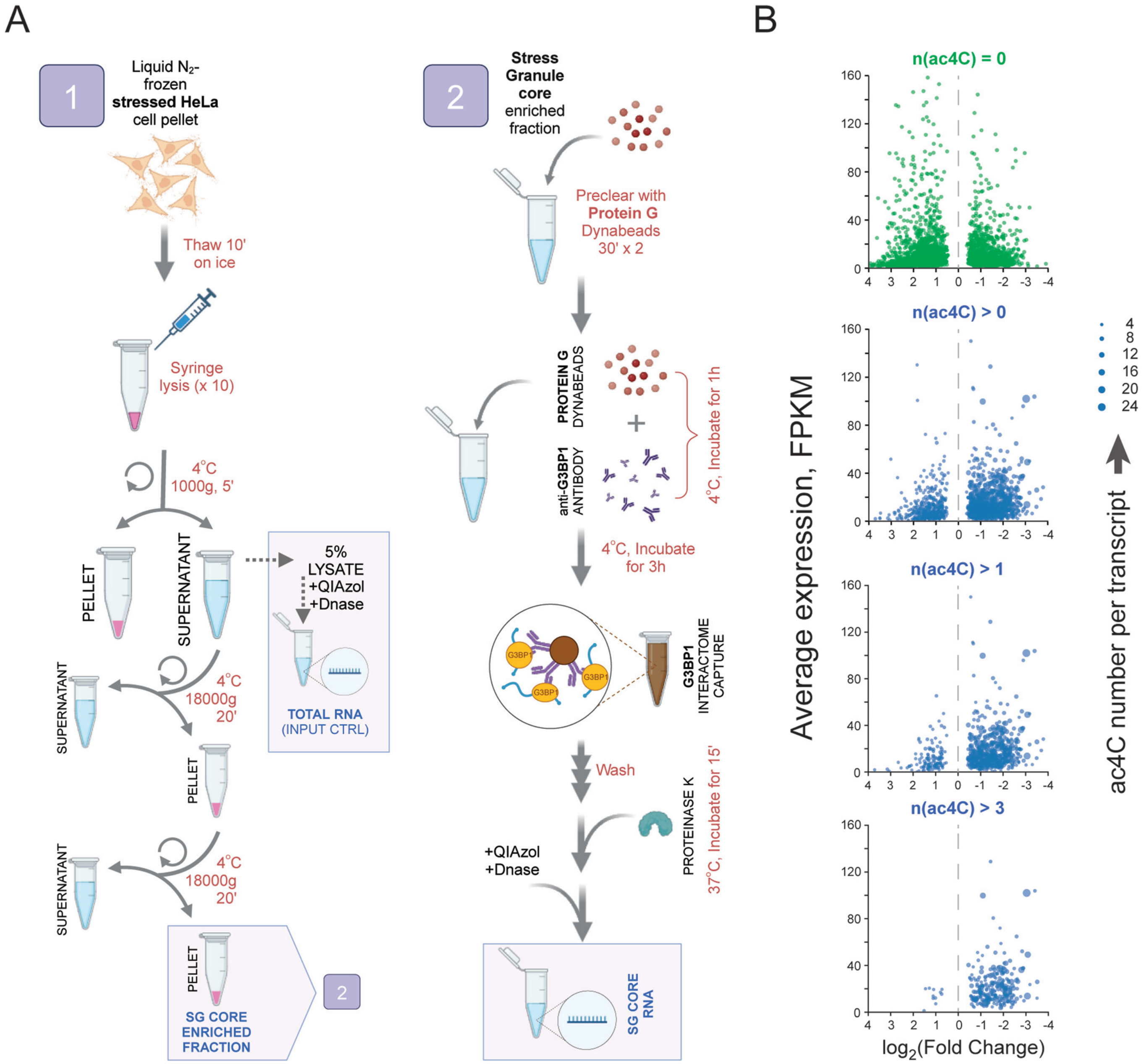

Figure EV2. Enrichment in SG, normalized expression and ac4C counts on transcripts.

(**A**) Schematic representation of SG RNA purification procedure; (**B**) The figure shows the transcripts with no ac4C sites ($n = 2983$ transcripts), more than zero ac4C sites ($n = 1792$ transcripts), more than one ac4C sites ($n = 865$ transcripts), and more than three ac4C sites ($n = 283$ transcripts) and the found log_2FC between SG and total RNA (negative FC means enriched in SG) as well as average normalized expression from RNA-seq shown as FPKM. RNA sequencing experiments were performed in 4 biological replicates.

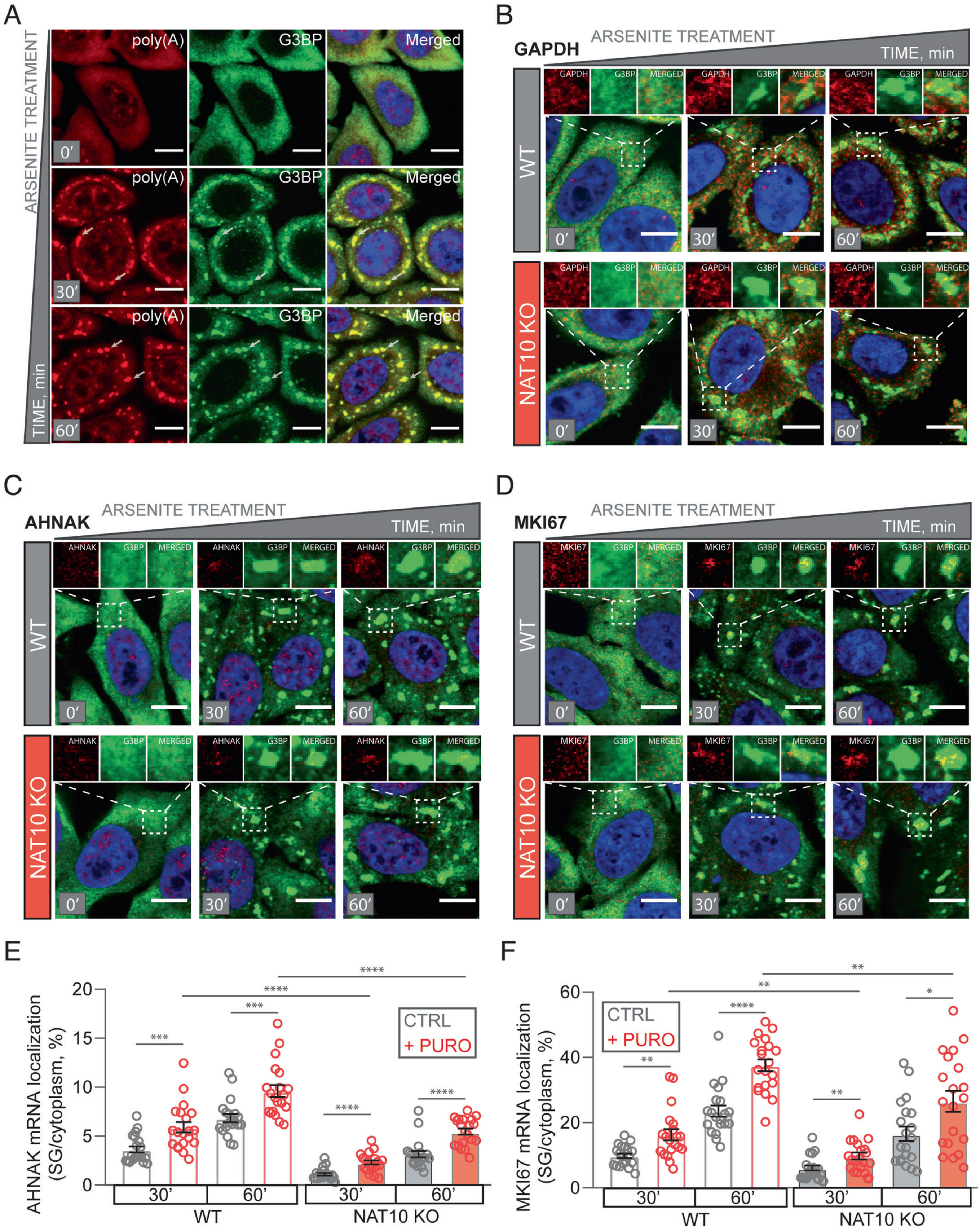
A
ARSENITE TREATMENT
TIME, min
poly(A)
G3BP
Merged
0′
30′
60′
B
GAPDH
ARSENITE TREATMENT
TIME, min
WT
NAT10 KO
GAPDH
G3BP
MERGED
C
AHNAK
ARSENITE TREATMENT
TIME, min
AHNAK
D
MKI67
ARSENITE TREATMENT
TIME, min
MKI67
E
AHNAK mRNA localization (SG/cytoplasm, %)
CTRL
+ PURO
30′
60′
WT
NAT10 KO
F
MKI67 mRNA localization (SG/cytoplasm, %)

 © The Author(s)

Figure EV3. mRNA localization to SG in WT and NAT10 KO HeLa cells in response to sodium arsenite treatment.

(**A**) smFISH-IF microscopy images of poly(A) RNA and G3BP accumulation into SG of WT HeLa cells in response to treatment with 0.5 mM $NaAsO_2$ for 0 min, 30 min or 60 min. Arrows indicate individual SG. Poly(A) RNA is depicted in red (as well as GAPDH mRNA in (**B**), AHNAK mRNA in (**C**) and MKI67 mRNA in (**D**)). For panels (**A–D**) G3BP in green, DAPI staining (nuclei) is in blue, scale bar 10 µm; smFISH-IF microscopy images of (**B**) GAPDH, (**C**) AHNAK and (**D**) MKI67 mRNA localization in response to treatment with sodium arsenite with 10 µg/ml puromycin for 0 min, 30 min or 60 min in HeLa WT and NAT10 KO cells; Quantification of the fraction of AHNAK mRNA (**E**) and MKI67 (**F**) in SG per cytoplasm in different conditions. 20 cells per each condition were counted. Data represented as Mean ± SEM. Unpaired two-tailed Student's *t*-test, $^{*}p < 0.05$, $^{**}p < 0.01$, $^{***}p < 0.001$ and $^{****}p < 0.0001$.

© The Author(s)

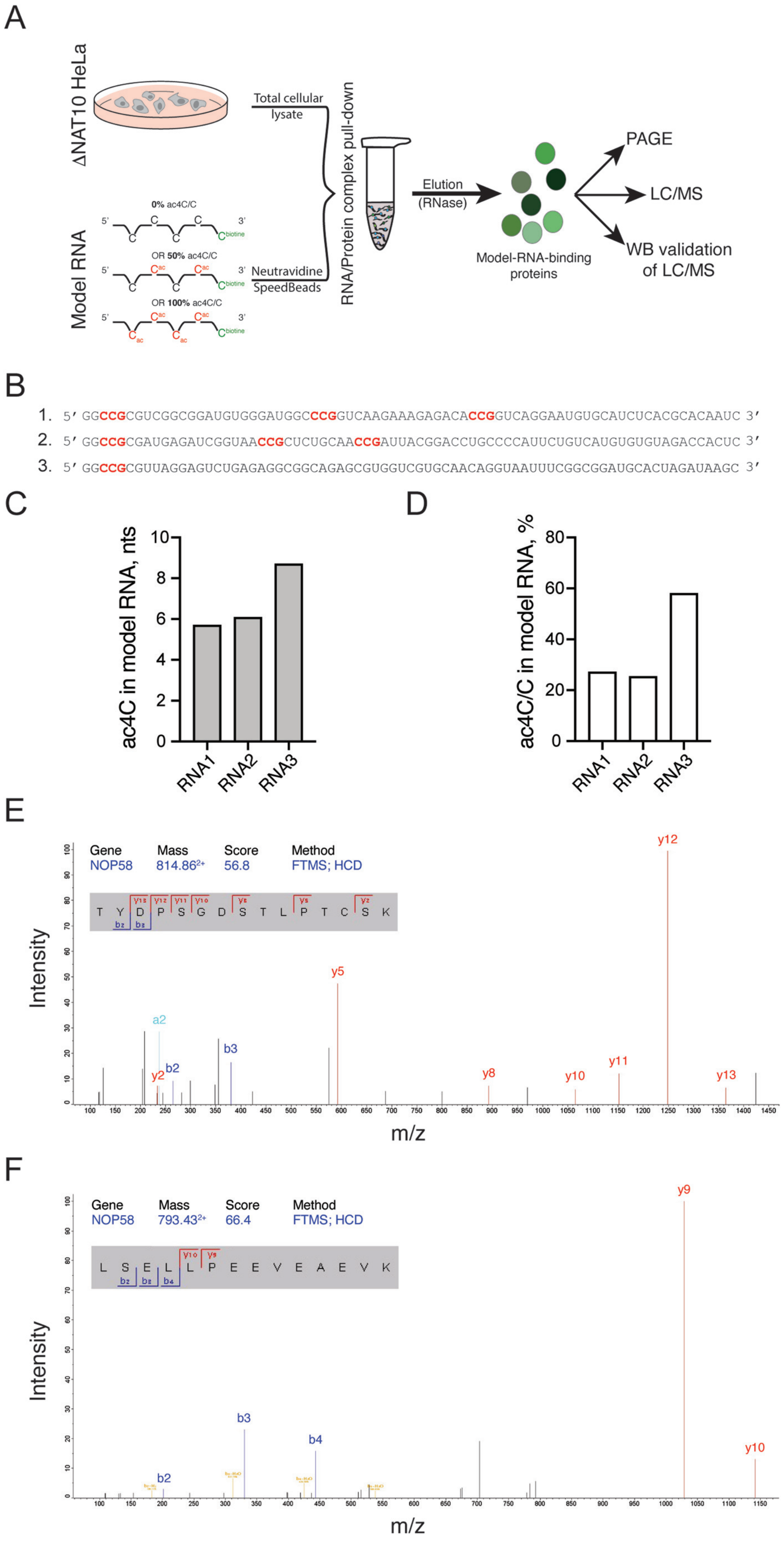
A
ΔNAT10 HeLa
Total cellular lysate
Model RNA
0% ac4C/C
OR 50% ac4C/C
OR 100% ac4C/C
5'
3'
Neutravidine SpeedBeads
RNA/Protein complex pull-down
Elution (RNase)
Model-RNA-binding proteins
PAGE
LC/MS
WB validation of LC/MS
B
1. 5′ GGCCGCGUCGGCGGAUGUGGGAUGGCCCGGUCAAGAAAGAGACACCGGUCAGGAAUGUGCAUCUCACGCACAAUC 3′
2. 5′ GGCCGCGAUGAGAUCGGUAACCGCUCUGCAACCGAUUACGGACCUGCCCCAUUCUGUCAUGUGUGUAGACCACUC 3′
3. 5′ GGCCGCGUUAGGAGUCUGAGAGGCGGCAGAGCGUGGUCGUGCAACAGGUAAUUUCGGCGGAUGCACUAGAUAAGC 3′
C
ac4C in model RNA, nts
RNA1
RNA2
RNA3
D
ac4C/C in model RNA, %
RNA1
RNA2
RNA3
E
Gene
NOP58
Mass
814.86²⁺
Score
56.8
Method
FTMS; HCD
T Y D P S G D S T L P T C S K
Intensity
m/z
F
Gene
NOP58
Mass
793.43²⁺
Score
66.4
Method
FTMS; HCD
L S E L L P E E V E A E V K
Intensity
m/z

Figure EV4. Mass spectrometry identification of NOP58 as an ac4C-binding protein.

The experimental strategy to identify ac4C-binding proteins is shown in panel (**A**). Panel (**B**) shows the sequences of three RNA oligos used in the immunoprecipitation experiments. Putative NAT10 recognition motifs (CCG) are highlighted in red. Panels (**C** and **D**) show the RNA MS analysis of oligos, where input ac4C/C ratio was 50% during IVT (in vitro transcription) and reflect on actual amount of ac4C in these oligos. (**E**) and (**F**) show peptides used to identify NOP58 in mass spectrometry experiments.

© The Author(s)
